# Environmental samples, a sensitive and practical alternative to individual bird sampling in the surveillance of H9N2 vaccinated turkey flocks

**DOI:** 10.1099/jgv.0.002279

**Published:** 2026-06-09

**Authors:** Leonard Kalvelage, Maximillian Casteel, Maria Hartmann, Timm C. Harder, Lothar Kreienbrock

**Affiliations:** 1 Department of Biometry, Epidemiology and Information Processing, WHO Collaborating Centre for Research and Training for Health at the Human-Animal-Environment Interface, University of Veterinary Medicine, Hannover, Germany; 2 WEK Veterinary Practice, Lohe 13, 49429 Visbek, Germany; 3 Institute of Diagnostic Virology, Friedrich-Loeffler-Institut, Südufer 10, D-17493 Greifswald-Insel Riems, Germany

**Keywords:** alternative monitoring, animal welfare, influenza A, poultry, practicability, safety

## Abstract

Surveillance for influenza A virus infections in vaccinated poultry flocks remains challenging due to animal welfare, logistical and financial constraints, particularly under current EU regulations governing high pathogenicity avian influenza (HPAI) vaccination. In this field study, we evaluated two environmental sampling (ES) methods – bedding boot swabs and drinker wipes – as alternatives to legally mandated individual bird testing, which involved monthly swabbing of 60 healthy birds (active surveillance, AS) and weekly swabbing of dead or sick birds (passive surveillance, PS). A total of 56 turkey flocks from 23 holdings in Lower Saxony, Germany, were monitored throughout the fattening period following single H9N2 vaccination at hatch. Semiquantitative reverse transcription polymerase chain reaction (RT-qPCR) revealed that, despite vaccination, H9N2 virus incursions occurred at least once in 76.8% (43/56) of flocks during the subsequent fattening period. Influenza A virus detection rate on the basis of individual samples was significantly higher by ES (24.1%) than through AS (10.65%; *P*<0.0001) or PS (15.6%, *P*=0.001). Overall, ES demonstrated superior performance in identifying 42/43 infected flocks (99.67%) compared with 30/43 (69.97%) by AS and 38/43 (88.37%) by PS. Heatmap and event-time analyses confirmed that ES reliably identified infection events very early and remained positive longer after initial detection. Non-invasive, animal-friendly ES was easy to implement and well accepted by farmers. Costs for ES surveillance were reduced by 73.5% compared to AS and PS. ES was found to be a sensitive, cost-effective and very practical alternative to conventional surveillance in influenza-vaccinated poultry, with direct relevance for future surveillance strategies in HPAI vaccination programmes.

**Impact Statement**

Effective surveillance is critical for evaluating and safeguarding avian influenza vaccination programmes, yet current bird-based monitoring is invasive, costly and difficult to sustain. This field study demonstrates that environmental sampling (ES) – using bedding boot swabs and drinker wipes – at least equals legally prescribed individual bird testing in detecting influenza A virus infections in vaccinated turkey flocks. ES often identified infected flocks earlier, and remained positive longer than conventional bird-based active and passive surveillance, while reducing surveillance costs by more than 70%. The approach was non-invasive, easy to implement, and well accepted by farmers, addressing major animal welfare and logistical constraints. These findings provide robust evidence that ES can be a sensitive, practical and scalable alternative for monitoring influenza virus circulation in vaccinated turkey flocks and offers a strong basis for its integration into future EU surveillance strategies, including those accompanying high pathogenicity avian influenza vaccination programmes.

## Introduction

Infections with avian influenza viruses (AIV) have a significant negative impact on poultry production worldwide, particularly in the case of high pathogenicity (HP) phenotypes of subtypes H5 and H7 [1]. The current panzootic spread of HPAIV of subtype H5N1, clade 2.3.4.4b, endangers the economic viability of poultry farms and also threatens avian biodiversity and even human health [2]. On top of that, infections of poultry farms with variants of AIV subtype H9N2 are highly prevalent, particularly in Asia and Africa, although short epizootic outbreaks have also been reported from Europe [3]. Despite the classification of these viruses as low pathogenicity (LP) AIV, they may cause clinical disease particularly in galliform poultry species resulting in impaired animal welfare and significant production losses. Co-infections with bacteria or parasites can exacerbate the course of infection, necessitating increased antimicrobial treatment [4]. Transmission and circulation of H9N2 infections between poultry holdings are currently counteracted by biosecurity and management measures developed and implemented to minimize the risk of introducing viruses into poultry farms and prevent onward transmission. Some H9N2 lineages exhibit zoonotic properties [5].

In geographical regions or production sectors where H9N2 infections have gained enzootic status in the poultry population, vaccination of poultry against H9N2 is proposed as a necessary additional pillar of prevention [6]. A similar conclusion has been reached for HPAIV H5N1 infections [7]. The benefits and risks of avian influenza vaccination in poultry, both in general and in particular for HPAIV or zoonotic AIV, have been extensively discussed [8,10]. An obvious objective is to minimize clinical disease in infected poultry, which would improve animal welfare and prevent production losses due to disease. In addition, vaccination is expected to reduce the amount of circulating virus by reducing susceptibility and limiting virus shedding in vaccinated birds following virus exposure.

It is widely acknowledged that vaccination campaigns must be meticulously planned and executed in a comprehensive manner, with due consideration given to the financial implications of vaccination and surveillance [9]. This includes the close monitoring of vaccinated flocks to determine their vaccination status and to continuously exclude the presence of field virus in vaccinated flocks [11]. The latter is a key factor ensuring the confidence of producers, trading partners and consumers in the safety and benefits of vaccination. The intensity of such surveillance, at least for notifiable (i.e. HP) AIV infections, depends on the overarching regulatory framework. The current legal requirements for HPAIV H5 vaccination in Europe call for a particularly high intensity and frequency of sampling and testing. These require, among other measures, PCR testing of individual swab samples of 60 heads of poultry per epidemiological unit every 4 weeks. This sampling can be carried out as part of the monthly clinical inspection of the animals, which must be conducted by the competent veterinary authority. This results in an extensive financial burden for a vaccination campaign, with surveillance costs easily accounting for up to 50% of the total cost of the vaccination campaign (B. Grasland, personal communication).

In response to these settings, several studies have been initiated to propose alternative surveillance strategies [12]. In addition to focusing surveillance on dead or sick birds in a vaccinated flock, samples from the immediate environment of birds in poultry houses have been proposed. These include air and dust samples, water and biofilms from drinking systems or litter. The findings, contingent on the particular poultry species, the production cycle and assorted other factors, have yielded encouraging results which suggested that environmental sampling (ES) has the potential to complement existing surveillance systems. A key benefit of this approach is the potential to reduce stress on animals, working time and costs [13]. To date, however, these systems have been evaluated mainly in non-vaccinated flocks.

In this context, an investigation was conducted in turkey fattening flocks in Germany, where vaccination against H9N2 infection had been introduced as a response to a regional epizootic situation. The data presented here demonstrate the potential advantages of ES in contrast to random sampling of individual birds in detecting the introduction and circulation of the AIV H9 virus in H9-vaccinated flocks.

## Methods

### Study collective

On the basis of interviews and production statistics, a collective of *n*=23 typical turkey fattening farms was recruited. A responsible person, usually from farm management, was appointed on each selected farm to communicate with the study group. Thus, the selection and composition of the farms represent a field-based observational study, including 23 commercial turkey holdings. A total of 56 flocks on 23 turkey fattening holdings were monitored throughout a single fattening period, which lasted from six to ∼22 weeks during July to October 2024.

### Vaccination of flocks

All birds had been vaccinated once in the hatchery subcutaneously in the neck, with 0.2 ml of the commercially obtained recombinant HVT/ND/H9 vaccine (NEWFLEND ND H9 [rHVT/ND/H9], Ceva Tiergesundheit GmbH). This was executed using an automated vaccine application system in accordance with standard operating procedures implemented in the respective hatcheries. No further booster vaccinations were carried out. Likewise, vaccination against HPAI was not performed.

### Animal sampling and arrangement of samples

We distinguished flock- and holding-levels:

- At flock level (syn. barn, stable), each flock is considered individually. If at least one sample (regardless of the sampling method) of the total number of samples tested per flock was PCR-positive, then the entire flock was considered positive.
- A holding (syn. farm) could be composed of different numbers of flocks. If at least one flock from a holding tested PCR-positive, then the entire holding was considered positive.

Swab sampling for active and passive surveillance (AS and PS) was carried out in accordance with the requirements of Delegated Regulation (EU) 2023/361 of 28 November 2022: monthly sampling of 60, randomly selected live animals per holding was carried out by obtaining combined oropharyngeal–cloacal swabs (OP/CL swabs) (Dryswab^®^, MWE). Monitoring began when the animals were moved from the rearing barn to the fattening barn at 6 weeks of age. AS was carried out every 4 weeks by the same person of the study group. Sampling comprised at least 60 swabs per holding. On holdings keeping three flocks, sampling comprised 20 swabs per flock. During sampling, the swabs were pooled into collective samples of ten swabs (Table 1).

**Table 1.** Study design and applied sampling schemes

Method	Sampling	Analysis	Result interpretation	Frequency
AS: Active surveillance* (EU Reg. 2023/361)	≥20 OP/CL per flock, 60 per holding	Pooled (*n*=10)	Ct ≤39 ->pool+	Every 4 weeks
PS: Passive surveillance (EU Reg. 2023/361)	Dead animals, OP/CL collected by staff	Pooled (*n*≤10)	Ct ≤39 ->pool+	Weekly
ES: Environmental surveillance	BS, DS, collected by staff	Individual	If either BS or DS is positive −>environmental sample +	Every 2 weeks

* Carried out by the same veterinarian from the study group.

Ct, cycle of thresholdBS, bedding boot swab; DS, drinker wipe; OP/CL, combined oropharyngeal cloacal swab.

For PS, dead animals were sampled continuously by the designated farm personnel on the respective holdings using combined OP/CL swabs. A maximum of ten samples were pooled in each flock (Table 1; see below). These pooled samples were collected every week on a flock-by-flock basis, stored in a refrigerator at 4 °C and sent for analysis weekly.

### Environmental sampling

**Fig. 1.**
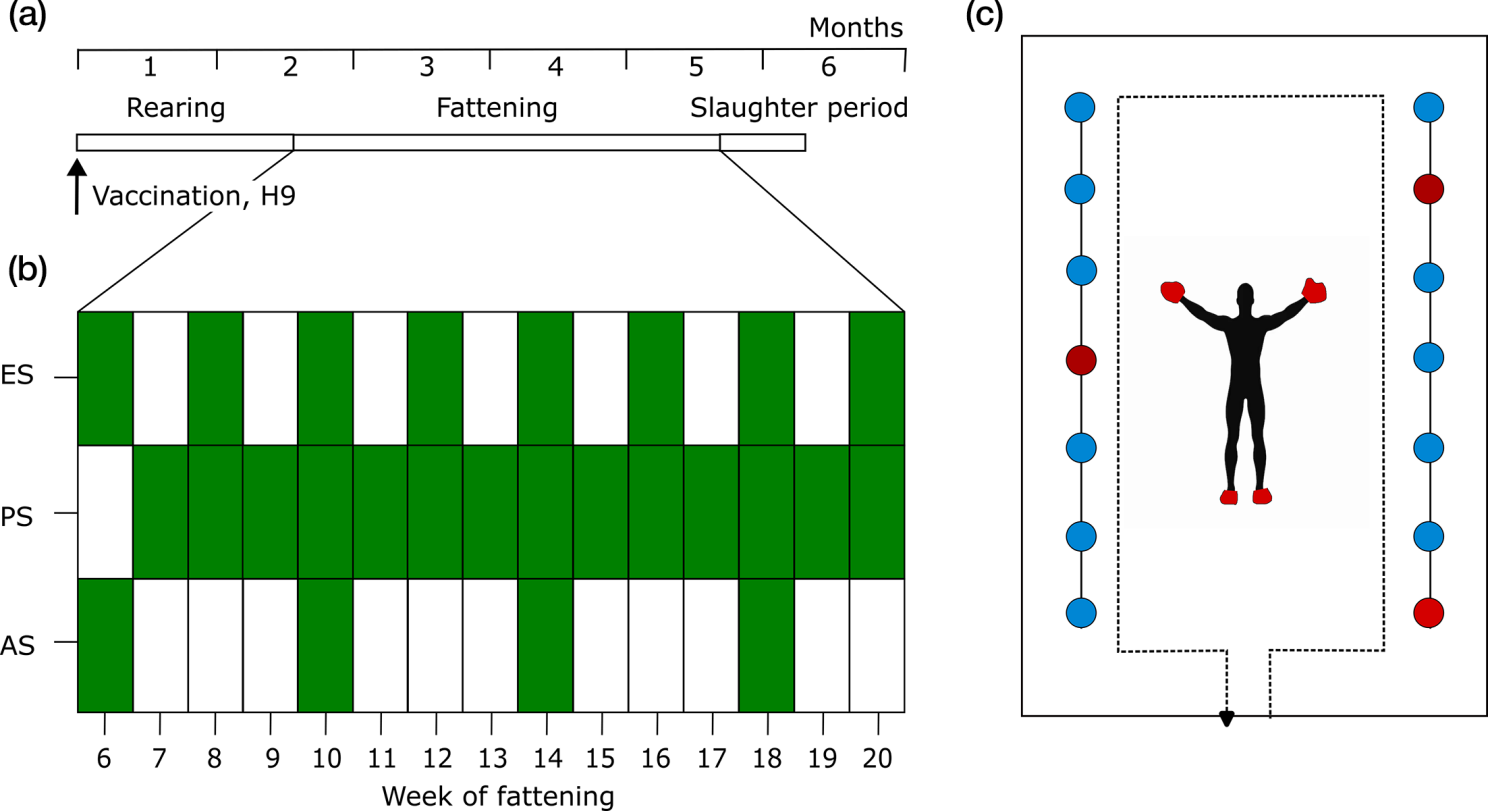
Scheme of avian influenza virus subtype H9N2 surveillance at turkey fattening holdings. (a) Temporal layout of a life cycle of fattening turkeys, including rearing and fattening phases, during up to 6 months. Turkeys received H9N2 vaccination once at the hatchery. (b) Scheme of sampling for H9N2 surveillance during the fattening phase: ES – environmental sampling by boot swabs and drinker wipes (see c), PS – passive surveillance (dead and sick birds), AS – active surveillance (60 swabs of healthy turkeys). (c) ES using bedding boot swabs (BS) and drinker wipe swabs (DS). A pathway along the outer drinker lines of both sides of a stable building was taken (dashed line) to obtain bootswab samples from the bedding. Along the drinker lines, biofilms of every fifth plate (red dots) were wiped using bootswabs fitted around the hands of the sampling person.

ES from the housing areas were obtained from bedding materials and drinking water. ES were taken using Poultry Boot Swabs MRD (Poultry Boot Swabs, TS/15-2A; Technical Service Consultants, United Kingdom) at fortnightly intervals by a trained responsible farm employee (Table 1). For this purpose, a boot-swab (syn. ‘sock’) each was pulled over the stable-specific rubber boots; in addition, a further sock each was pulled over the gloved hands of the sample collecting employee (bedding boot swab, BS; Fig. 1). This covered all the ends of the person’s limbs with boot swabs. Sampling of the bedding was carried out by walking along the two outer drinking lines in each flock (Fig. 1). One boot swab pulled over a hand was used per drinking line to systematically take wipe samples at every fifth round drinker. The wipe sample was taken from the transition area between the water surface and the inner wall of the drinker (drinker wipe swab, DS). To obtain representative biofilm samples, the entire contact area between the water and the surface of the drinker was covered as the swab-covered hand travelled completely around the body of the drinker. After sampling, the boot swabs from the drinker biofilms and the bedding along the drinker lines were placed in separate bags (bags A and B).

### Laboratory processing of animal samples

The OP/CL swab samples, which were collected from individual birds and then combined into pools of up to ten, were placed in 50 ml centrifuge tubes at the holding. In the laboratory, 1 ml of PBS was added per swab to each collection tube and the tubes were vortexed for 30 s. Afterwards, the samples were centrifuged at 3,000 r.p.m. for 5 min. Two millilitres of the supernatant were preserved at −80 °C for RNA extraction and as a reserve sample.

### Laboratory processing of environmental samples

ES boot swab samples were each filled with 100 ml of PBS (50 ml per swab) and mixed for 1 min using a BagMixer 400 homogeniser (Interscience, France). Fifty millilitres of each sample were transferred to a centrifuge tube and centrifuged at 3,000 r.p.m. for 5 min and 2 ml of the supernatant were preserved at −80 °C until RNA extraction.

### RNA extraction and real-time RT-PCR (RT-qPCR)

The extraction of viral RNA from each sample was conducted in accordance with the protocol stipulated by the manufacturer, utilising the Sample Preparation Kit IV (Hygiena) within the KingFisher Flex system (Thermo Fisher Scientific, Waltham, USA). The RNA was eluted in 100 µl of elution buffer. The eluates were divided into four aliquots of 20 µl.

Extracted RNA was analysed by a commercial Taqman-based semiquantitative reverse transcription polymerase chain reaction (RT-qPCR) targeting the influenza A virus M gene (BioFlux Avian Influenza Virus, Hangzhou, China) according to the manufacturer’s conditions on a BioRad CFX96 thermocycler. Positive samples were further analysed using a triplex RT-qPCR-Kit (BioFlux PCR/RT-PCR Kit H5 H7 H9 Subtype, Hangzhou, China) to confirm H9 infection and exclude infections with H5 or H7. In accordance with the manufacturer’s recommendations. Positive and negative RT-qPCR results were distinguished by a cycle threshold (Ct) value of 39, below which samples were defined to be positive. Pooling of up to five samples was performed to reflect routine diagnostic practice and reduce analytical costs.

### Data preparation and statistical analysis

All anamnestic information about the individual samples, along with the results of the tests, was recorded and documented in an exclusive laboratory information management system developed for the veterinary practice network (Münch IT Solutions). This data was then exported to an Excel spreadsheet (version 16.77, Microsoft). Data records were structured to enable a plausibility check and subsequent statistical analyses using SAS Studio [version 3.81 (Enterprise Edition)] (SAS Institute Inc., Cary, NC, USA).

Quantitative measurements were converted into dichotomous outcomes according to the predefined thresholds. In addition to the descriptive presentation of qualitative data, heat maps were generated using the PROC SGPLOT procedure in SAS. Positive findings were depicted in red, negative findings in green and fields without examination in white. Inductive statistical analyses included chi-square testing, survival time analyses, one-way ANOVA and Tukey–Kramer post-hoc test. Chi-square tests were performed with the PROC FREQ procedure and *P*-values <0.05 were considered statistically significant. Based on the data shown in Table 2, relative detection ratios shown in Table 3 were calculated as the ratio of total detection rates between two surveillance methods (row/column). Values >1 indicate higher detection by the method in the row; values <1 indicate lower detection. Kaplan–Meier curves were generated with the PROC LIFETEST procedure, and hazard ratios were estimated using COX regression with the PROC PHREG procedure. One-way ANOVA was performed using the PROC GLM procedure, and pairwise comparisons were performed with the Tukey–Kramer post-hoc test. *P*-values<0.05 were considered statistically significant.

**Table 2.** Detection of influenza A virus RNA M- and H9-targets by RT-qPCR in turkey flocks vaccinated against influenza A virus subtype H9

Surveillance method	Samples (n/N, %)*	Flocks (n/N, %)†	Holdings (n/N, %)‡
Active surveillance (AS)	59/554 (10.65%)	30/56 (53.57%)	15/23 (65.22%)
Passive surveillance (PS)	79/508 (15.55%)	38/56 (67.86%)	15/23 (65.22%)
Environmental surveillance (ES, combined DS+BS)	99/411 (24.09%)§	42/56 (75%)	16/23 (69.57%)
Drinker wipe swabs (DS)	95/411 (23.11%)	42/56 (75%)	16/23 (69.57%)
Bedding boot swabs (BS)	69/411 (16.79%)	39/56 (69.64%)	16/23 (69.57%)

*,†,‡n, number of samples/flocks/holdings positive by RT-qPCR for M- and H9-targets, N, total number of samples/flocks/holdings investigated.

§Samples/flocks/holdings positive by RT-qPCR for M- and H9-targets in either DS or BS combined.

**Table 3.** Detection of H9N2 incursion in vaccinated fattening turkey flocks by different surveillance strategies combined; 43 flocks were detected as infected with AIV subtype H9 at any time during the fattening phase

Detection category	N flocks (%)
All three methods positive	28 (65.12%)
AS, PS positive	1 (2.33%)
AS, ES positive	1 (2.33%)
PS, ES positive	9 (20.93%)
Only ES positive	4 (9.3%)
**Total H9-positive flocks**	43 (100%)

## Results

In the present study, the primary focus was on analysing and comparing the different surveillance strategies. The analyses were not extended to efficacy evaluation of the vaccine used or the health status of flocks. All data used for the analyses are presented in raw format in Supplement 1 (available in the online Supplementary Material).

### Demographic characteristics of the participating holdings and flocks

A total of 23 commercial turkey holdings participated in the study. The number of flocks per holding ranged from one to a maximum of five, resulting in a total of 56 flocks being included. The participating holdings were located in the districts of Oldenburg and Cloppenburg in the German federal state of Lower Saxony. The young turkeys came from various rearing houses and hatcheries. With the exception of one flock, only male turkey poults of the B.U.T. Six Aviagen Turkey breeding line were housed. On average, 3,800 turkeys per flock were kept within a range of minimum 1,450 and maximum 7,049 animals per flock.

The monitoring period covered only the fattening phase, which extends to ∼22 weeks of age. Birds within each flock were kept together in groups without any internal physical separation. Flocks were supplied with feed by different commercial feed companies. A total of 17.86% of the flocks were equipped with an integrated winter garden. All birds were housed in flocks with natural gravity ventilation. In 55.36% of the flocks, air inlets were controlled externally by shutters, whereas 44.64% used flaps. Straw was used as bedding material in 62.5% of the flocks, while 37.5% used sunflower meal. Litter management practices differed: In 42.86% of the flocks bedding was regularly topped up, while in 57.14% it was loosened by mechanical tilling.

Since all turkey flocks were located in the same geographic region and run under very similar conditions, they were considered subject to comparable levels of infection pressure and risk. The study did not include systematic monitoring of the animal health status, or therapeutic interventions administered during the observation period. Consequently, such measures were not considered in the analyses.

### Detection of H9N2 infections in vaccinated turkey flocks

As a general measure at flock-level, regardless of the surveillance method, a total of 43 out of 56 flocks (76.79%) were detected as infected with AIV subtype H9 at any time during the fattening phase. At holding level, this translates into 16 out of 23 holdings (69.6%). Details of the RT-qPCR analyses of a total of 1,884 samples tested for influenza A virus RNA M- and H9-targets is presented in Table 2.

Overall, detection rates differed considerably between monitoring approaches. AS showed the lowest proportion of positive individual samples (10.65%), while PS detected 15.55% positives. Combined ES (DS+BS) yielded the highest detection rate (24.09%), with DS (23.11%) detecting a larger number of positives than BS (16.8%). At flock and holding level, these differences in individual sample analysis were reflected consistently: ES identified four more positive flocks and one holding compared to AS and PS; ES failed to detect infection in only one case, which tested positive by both AS and PS (Table 3). At flock level, ES achieved a detection rate of 97.67% (42/43 infected flocks). In comparison, the detection rates of AS and PS were 69.76 and 88.37%, respectively.

Detection rates differed significantly between surveillance methods (Table 4). ES showed higher detection rates than both AS and PS (*P*<0.0001 and *P*=0.0011, respectively). Within ES, DS showed higher detection rates than BS (*P*=0.0233). AS consistently yielded lower detection events compared to ES combined (*P*<0.0001), as well as compared to DS (*P*<0.0001) or BS (*P*=0.0054). PS detected more positives than AS (*P*=0.0176) but did not differ significantly from BS (*P*=0.6119). Relative detection ratios further emphasize these findings: Combined ES more than doubled the likelihood of detection compared to AS, while PS was ∼1.5-fold more likely to detect H9 incursions compared to AS.

**Table 4.** Statistical comparison of Influenza A virus detection rates for individual samples stratified by surveillance approaches

Above diagonal: Chi-square *P*-values.

Below diagonal: Relative detection ratio (row/column).

Calculations are based on values shown in Table 2.

**Fig. 2.**
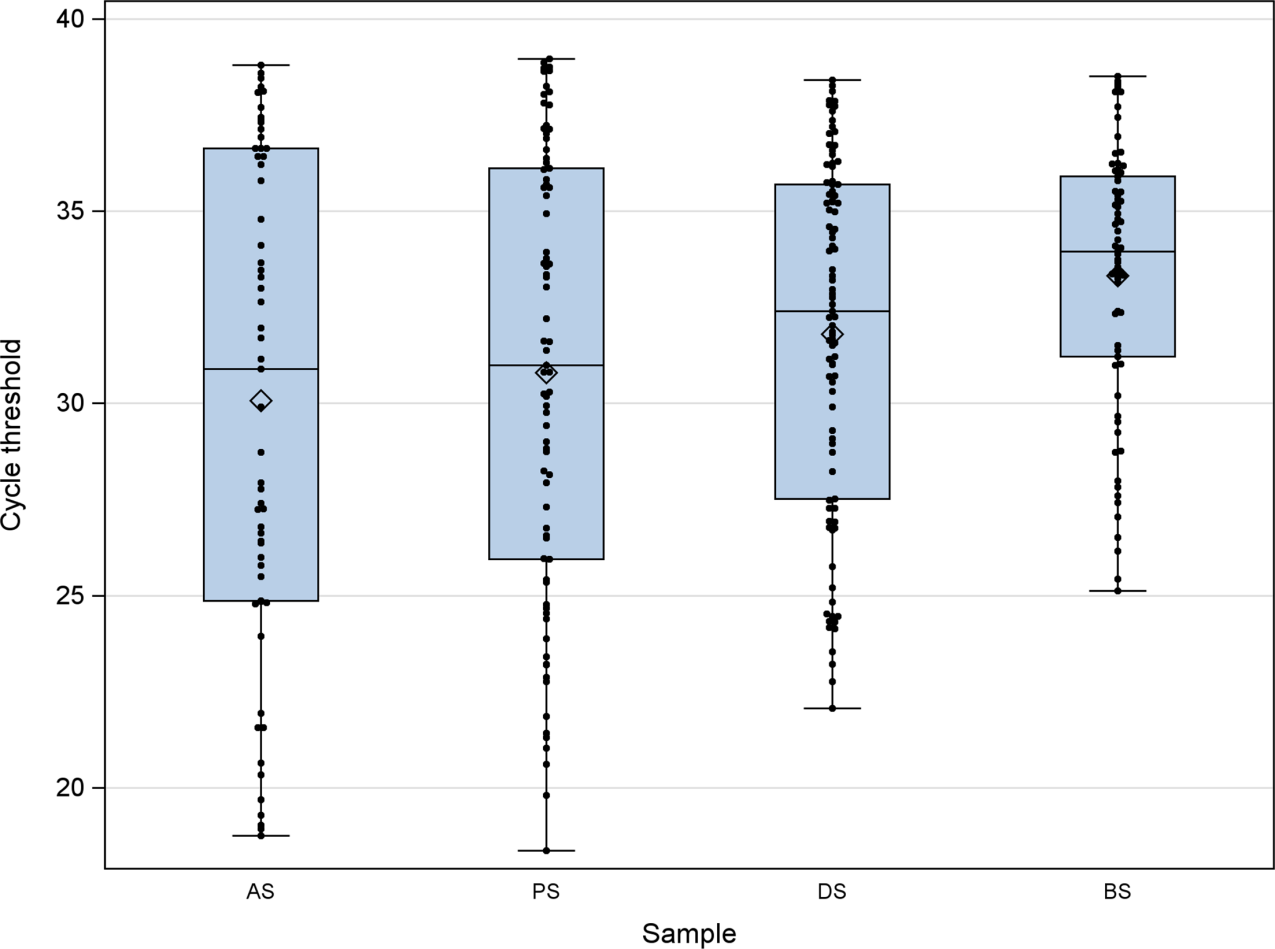
Comparison of viral loads detected by ES, AS and PS in various matrices by RT-qPCR. Samples with Ct≤39 in RT-qPCRs targeting the M gene were compared.

The detectability of viral RNA is determined by both the analytical sensitivity of the assay and the viral load present within a given sample matrix. Low viral loads are more likely to escape detection, even when highly sensitive methods, such as RT-qPCR, are employed. To assess potential differences in viral loads associated with the sample matrices used in the three surveillance strategies, RT-qPCR Ct values <39 were quantitatively compared. As illustrated in Fig. 2, there are statistically significant differences in viral load among the matrices (ANOVA *P*=0.0021), indicating that the probability of detecting H9N2 viral RNA differs significantly between BS, DS, AS and PS sampling approaches. The Tukey–Kramer test revealed that BS samples had statistically significant higher Ct-values, i.e. lower viral loads, than AS (*P*=0.0023) and PS (*P*=0.0159). No other pairwise comparison reached statistical significance.

### Efficacy evaluation of different surveillance techniques during the fattening period

**Fig. 3.**
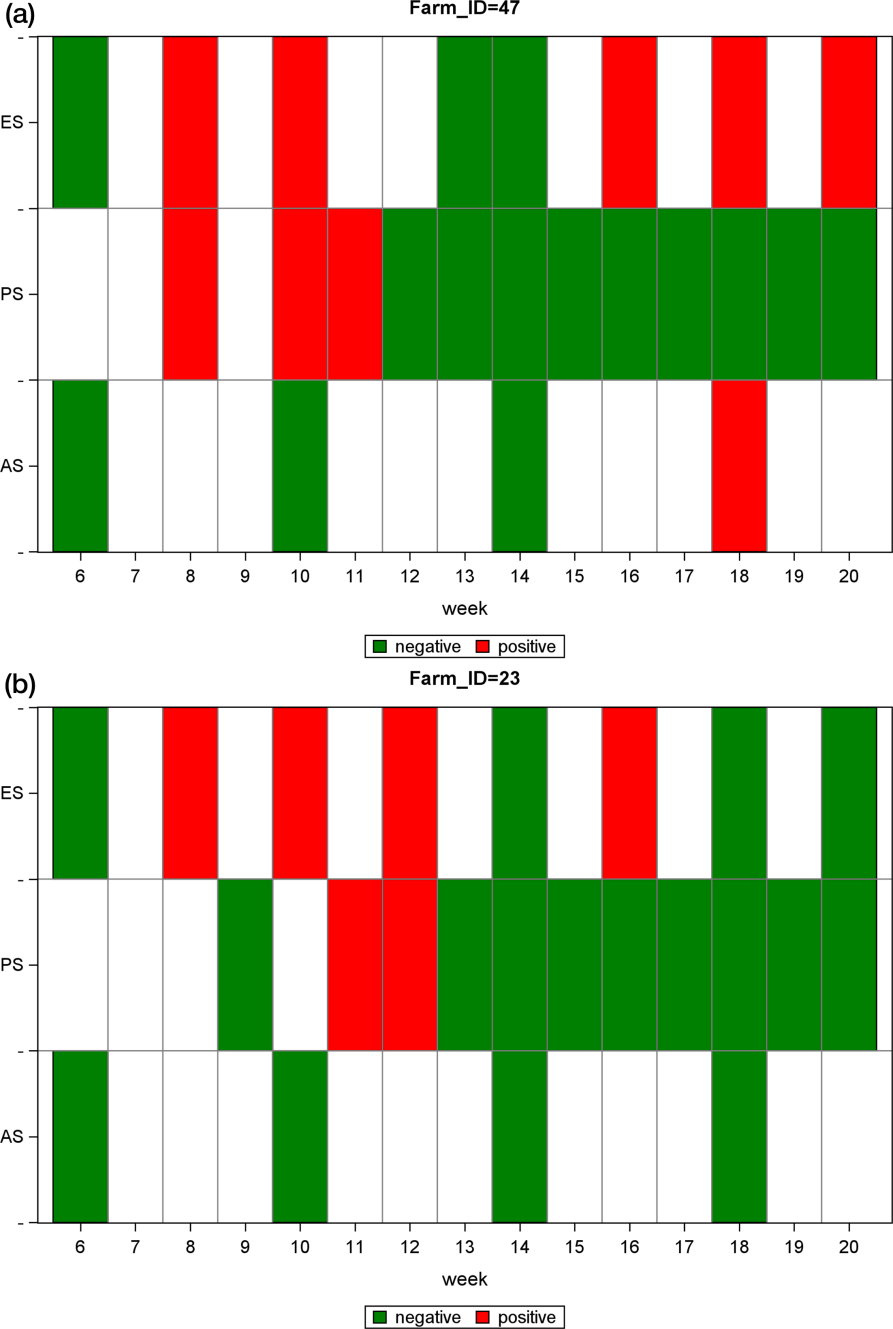
Heatmap depicting the performance of different surveillance systems stratified by fattening week for two selected farms (white boxes indicate weeks in which no samples were collected).

To check the practical performance of the different surveillance concepts, AS, PS and ES were compared during the fattening period within a longitudinal approach. Heatmaps on flock-level and statistical analysis of the overall data were carried out to assess the earliest time point of detection of an incursion and to follow-up infection progression. Fig. 3 displays two typical examples selected for illustration (similar heatmaps for all flocks included in this study are presented as Supplement 2).

For farm_ID 47 (Fig. 3a), ES and PS samples tested positive for influenza A virus RNA simultaneously for the first time at week 8 of the fattening period. In contrast, AS remained negative until week 18. Notably, both ES and PS samples showed sustained positivity over an extended period. A second flare-up of H9 infections in farm_ID 47 was detected by ES and AS, but not PS, after week 16. A comparable pattern was observed in farm_ID 23 (Fig. 3b), where ES facilitated earlier detection of Influenza A virus RNA at week 8 in contrast to PS testing positive not before week 11, while AS consistently tested negative.

**Fig. 4.**
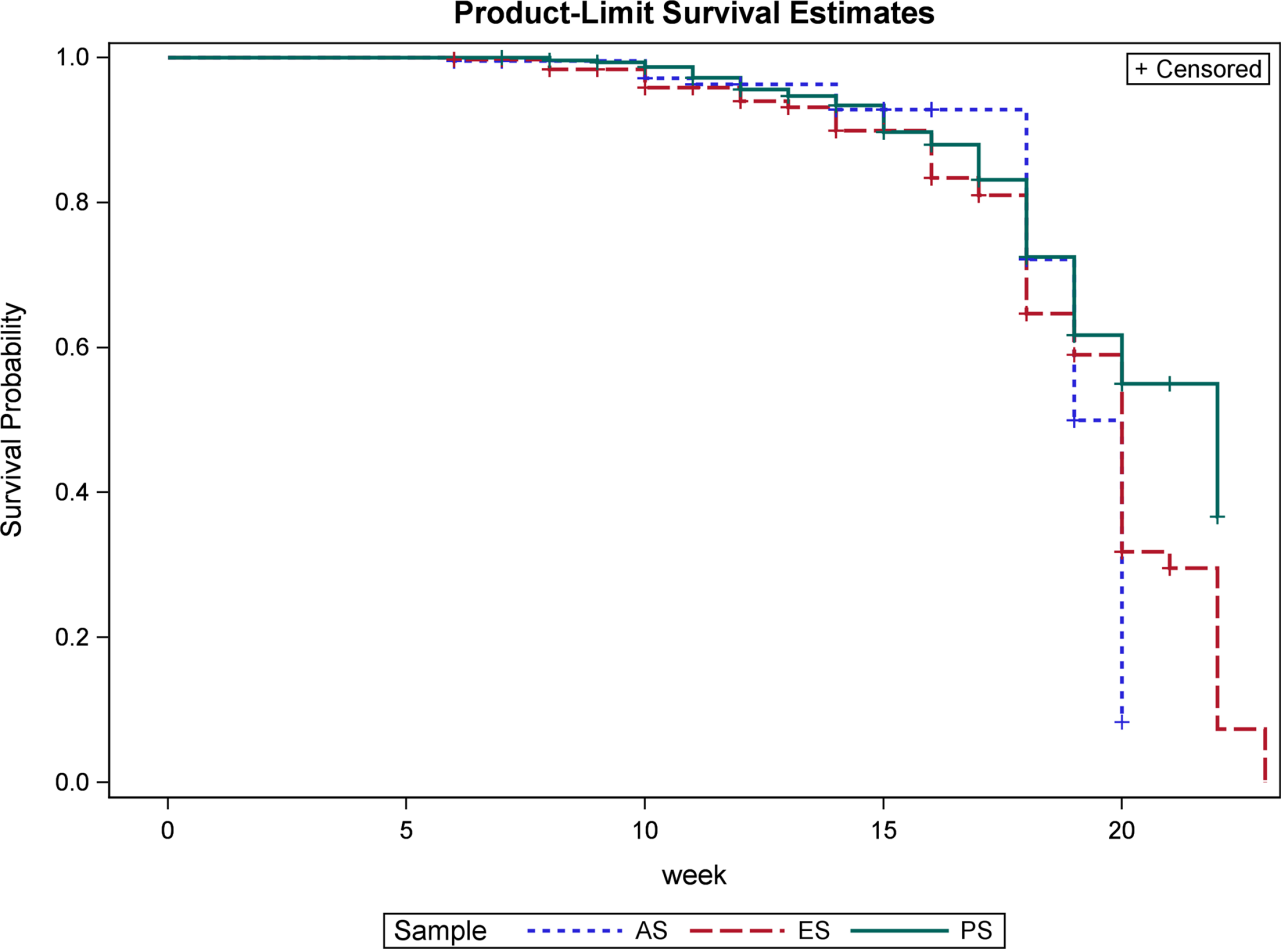
Kaplan–Meier-survival analysis comparing time to first detection of Influenza A virus infection across three surveillance strategies: active surveillance (AS), passive surveillance (PS) and environmental sampling (ES).

The descriptive observations derived from the heatmaps were further analysed by a survival time analysis using a Cox regression model to assess whether the timing of first detection of influenza A virus varied systematically between the different surveillance strategies (AS, PS and ES). Compared to AS, which should represent a reference system for surveillance, the hazard ratio for PS was 0.798 (95% confidence interval: 0.525–1.212; *P*=0.280) and for ES it was 1.139 (95% confidence interval: 0.0754–1.721; *P*=0.553), i.e. neither PS nor ES showed a statistically significant difference in the first identification of Influenza A virus RNA of subtype H9. Yet, the Kaplan–Meier curves nevertheless suggested that positive results tended to be found earlier by ES (Fig. 4).

### Cost evaluation: advantages of ES in reducing surveillance costs

A flat rate of 30€ per RT-qPCR analysis was assumed to represent current average laboratory costs, although variation may exist. We assumed costs for a surveillance period of 4 months, i.e. 16 weeks, covering the entire fattening period between week 6 and week 22. For ES, total costs were estimated at ∼600€ per fattening cycle. This comprises eight sampling points resulting in eight pool PCRs for each DS and BS. This amounts to each 480 € = 2×8×30€/PCR. An additional 60€ is assumed for laboratory and material expenses for each matrix (DS and BS), resulting in a total ES cost of 600€ per fattening period. According to EU delegated regulation 2023/361, both AS and PS are required: For AS and PS, analytical costs were calculated as 24 PCRs for AS (24×30€ = 720€) and 16 PCRs for PS (16×30€ = 480€). For PS, an additional 60€ is assumed for laboratory and material expenses per fattening period, resulting in 540€. Combined analytical costs for AS and PS, therefore, amounted to 1,260 € in total. For swabbing live birds veterinary skills are required; in addition regulation 23/361 requires monthly visits for clinical inspection by the veterinary authority. Four veterinary visits were added at 250€ each (travel and sampling time, according to the German Veterinary Fee Regulation, [14]), resulting in overall estimated costs of ∼2,260€ per fattening cycle for the legally prescribed surveillance versus 600€ for ES as practised here.

## Discussion

Over the past 10 years, there have been repeated outbreaks of H9N2 infections in fattening turkeys in Germany, resulting in considerable animal welfare problems and economic losses. Currently, the region is plagued by incursions of HPAIV H5N1, leading to frequent losses of flocks through culling orders [15]. In these situations, sanitary and biosecurity measures alone have been shown not to be able to fully prevent incursions or contain infections; vaccination campaigns, therefore, came into focus as a further pillar of preventive measures. For H9, the authorisation of an H9-specific vaccine for turkeys in Europe in 2023 enabled the field use of AIV-specific vaccines at a wider range in turkey production centres in Germany. This enabled to conduct the investigation presented here as all of the flocks included in this study made use of the H9 vaccination. Although there was uncertainty about the efficacy of this H9 vaccination campaign, the present study was not intended to evaluate the vaccinations' efficacy in principle. Instead, the main focus was on comparing various surveillance strategies, at least partially based on legally regulated EU measures in HPAIV-vaccinated poultry flocks, to characterize their practical efficacy to detect H9N2 viruses circulating in vaccinated flocks.

To date, only a few studies have described the methodology of ES for surveillance of influenza A vaccinated poultry flocks in detail [16]. Here, we monitored a total of 56 vaccinated turkey flocks by RT-qPCR for H9N2 virus incursions over the course of one fattening period. An unexpectedly high percentage (76.79%) of vaccinated flocks had at least one outbreak of H9N2, as measured by various surveillance techniques, despite a single vaccination with a recombinant H9-HVT vaccine. An evaluation of the vaccination campaign in terms of its efficiency, which would also have to include an examination of the health status of the flocks, was explicitly excluded here. In a previous study, experimental H9N2 exposures of chickens vaccinated with the vaccine used here also showed continued susceptibility, replication and shedding of the H9N2 challenge virus, albeit in smaller amounts and for a shorter time compared to unvaccinated controls; moreover, clinical protection was achieved [17].

As demonstrated in Table 2, in 42 of the H9N2-positive flocks, at least one ES sampling event produced a positive result for Influenza A Virus, whereas AS yielded a positive result in only 30 flocks. Consequently, infections within 12 flocks would not have been detected by AS alone. The PS approach resulted in at least one positive finding in 38 of the flocks, indicating that, in comparison with the ES method, four infections would also have gone undetected using solely the PS approach. ES missed out one flock, which tested virus-positive by AS and PS (Table 3). With the exception of this flock, ES produced positive results when at least one of the conventional surveillance methods also yielded positive results. As demonstrated in Table 4, the benefits of ES over AS and PS in identifying incursion events are statistically significant. The ES comprised two types of ES, i.e. drinker wipes and bedding swabs. For the purpose of qualitative analysis, both sample matrices were combined. However, it was observed that drinker wipes demonstrated a higher rate of detection in comparison to bedding swabs which might be due to matrix effects (Fig. 2). Importantly, the observed differences in analytical sensitivity did not compromise the diagnostic performance of ES. Although BS showed higher Ct values (i.e. lower viral loads) than AS, ES still detected 97.67% of all H9N2-positive flocks, clearly outperforming AS (69.97%) and PS (88.37%). Even BS alone, despite its lower viral loads, achieved a higher case detection (92.86%) than either AS and PS. These results demonstrate that ES provides a robust and highly sensitive approach for identifying infection events, even under conditions of low or heterogeneous viral loads. ES likely increases detection probability because viral material accumulates in the environment, integrating shedding from multiple animals over time, whereas individual sampling provides only a single time-point snapshot. Environmental contamination from outside, e.g. wild birds, potentially giving rise to false-positive ES results are considered highly unlikely. In most commercial turkey fattening, and thus also in this study, free-range flocks are virtually non-existing. That renders detectable contamination from wild birds near nil unless an infected wild bird would be found within the stable (which was never detected). However, once minute contamination with infectious virus took place and turkeys picked it up, infection and virus spread would be expected to commence which then would become detectable with much higher likelihood by ES.

It is important to note that different drinker systems are often used in turkey fattening farms, and difficulties may arise in achieving representative sampling of biofilms with certain types of drinkers, such as nipple drinkers without drip trays. The combination of drinker wipes and bedding swabs, therefore, is intended to compensate for such inconsistencies and is believed to increase the overall diagnostic consistency. In addition, the selection of ES matrices to be sampled, the sampling frequencies, and other such measures may necessitate further optimization in surveillance management. Consequently, the transfer of findings of this study to other poultry farming practices and, in particular, poultry species without the support of additional research should be done with caution only.

In comparison with direct animal sampling, earlier detection of H9N2 virus incursion by ES was observed in several turkey flocks (see Fig. 3 and supplementary heatmaps). Moreover, in certain instances, the virus remained detectable by ES for protracted periods following the initial diagnosis when direct animal sampling no longer indicated virus presence (Fig. 3). However, the results of the survival time analysis using Kaplan–Meier curves, the Cox regression model and pairwise comparisons did not confirm a significant difference in the time to first positive detection between ES and AS or PS. Conversely, there is no indication that ES would delay the detection of incursions. In contrast, the inherent dependency of PS on bird mortality necessitates mention, as it affected the consistency of sample collection. As demonstrated in the examples presented in Fig. 3 and in the supplementary material, the collection of PS samples proved inconsistent on a weekly basis, owing to the scarcity and even absence of deceased or sick birds. In contrast to HPAIV, the mortality rates of LPAIV infections may be very low, especially during the initial phase of an incursion. Consequently, early infection events may go undetected if surveillance is based on PS. However, the ES method, predicated on the detection of virus excreted from infected poultry, may already capture the presence of the virus, independent of mortality events, thereby enabling earlier detection. In this respect using a highly sensitive detection technique, such as RT-qPCR, is recommended; quantitative comparisons showed that equivalent viral loads were present in ES, AS and PS samples. This is expected to shift in favour of PS in the case of HPAIV-related surveillance.

Besides these diagnostic features, a significant benefit of ES is the non-invasive nature of the sampling procedure, which does not cause discomfort to the animals and can be performed by a person designated by the official authority without the need for specialised veterinary expertise. It is anticipated that this will be met with a high level of acceptance in practice, as it has the potential to reduce logistical requirements and financial burden fundamentally. Indeed, the economic analysis demonstrated the cost-effectiveness of ES. The key features that have been identified as being instrumental in the reduction of costs are a reduction in logistical complexity regarding veterinary visits and the option to integrate ES into the routine operational processes of the holding. Based on the average costs for sampling and laboratory work in Germany, this results in savings of around 73.5% for the ES. The calculation is exclusively based on the fattening period; hence, it does not encompass the potential additional expenses associated with the rearing phase during the first 6 weeks after hatching.

## Conclusion

The present results demonstrated that in turkey fattening holdings ES combining drinker wipes and bedding swabs is a practical, cost-effective and animal-welfare-compliant recommendable alternative to the current legally mandated surveillance of influenza A virus as prescribed for HPAI-vaccinated poultry in accordance with Delegated Regulation (EU) 2023/361. ES facilitated a timely and precise identification of AIV H9N2 in instances of incursion into H9N2 vaccinated turkey flocks. The fact that sampling is technically undemanding (and could even be carried out by farm staff), as well as the option of combined analysis of drinker and bedding samples, further highlights the potential of this method to simplify and optimize monitoring strategies. In comparison with instances of HPAI virus incursions into HPAI vaccinated flocks, it is to be expected that there will be a lower mortality rate for H9-vaccinated flocks where LPAI H9 viruses are circulating. This places a significant burden on PS, as the method is predicated on the occurrence of mortality. The results of the study demonstrated that, in fact, PS failed to identify four farms with incursions, which were detected by ES. ES, conversely, is predicated on the excretion of viruses by infected birds. It is hypothesised that excretion amplitudes for HPAIV and LPAIV, in particular in vaccinated flocks, exhibit a larger degree of similarity than mortality rates. Consequently, ES also emerges as a potential surveillance strategy for HPAI-vaccinated turkey flocks. Furthermore, ES has previously been shown to be highly informative at live poultry markets [16]. However, further studies are required to systematically evaluate the validity and applicability of this method under different practical conditions, different poultry species, rearing systems and influenza A virus sub- and pathotypes before adaptations of legal frameworks can be mandated.

## Supplementary material

10.1099/jgv.0.002279 Supplementary Material 1.

10.1099/jgv.0.002279 Supplementary Material 2.

## Abbreviations

AIV: avian influenza viruses
AS: active surveillance
BS: bedding boot swab
ct: cycle threshold
DS: drinker wipe swab
ES: environmental sampling
HP: high pathogenicity
HPAI: high pathogenicity avian influenza
LP: low pathogenicity
LPAI: low pathogenicity avian influenza
OP/CL: oropharyngeal–cloacal swabs
PS: passive surveillance
RT-qPCR: semiquantitative reverse transcriptase polymerase chain reaction
