# Supplementary material for: Environmental samples, a sensitive and practical alternative to individual bird sampling in the surveillance of H9N2 vaccinated turkey flocks
Source: J Gen Virol. 2026 Jun 9;107(6):002279. doi: 10.1099/jgv.0.002279 (PMC13249264; doi:10.1099/jgv.0.002279)
Supplement: Supplementary Material 2. [file jgv-107-02279-s002.pdf]

Supplement 2: Heatmaps depicting the performance of different surveillance systems stratified by fattening week for two selected farms (white boxes indicate weeks in which no samples were collected). The ID of each farm is indicated on the top of each subfigure. Summarized results are color-coded and are presented at the beginning of the file.

**Detection category**	**N flocks (%)**
All three methods positive	28 (65.12%)
AS, PS positive	1 (2.33%)
AS, ES positive	1 (2.33%)
PS, ES positive	9 (20.93%)
Only ES positive	4 (9.3%)
**Total H9-positive flocks**	43 (100%)

**Farm_ID**	**AS**	**PS**	**ES**
1	positive	positive	positive
2	negative	positive	positive
3	negative	negative	positive
4	negative	negative	positive
5	negative	negative	positive
6	negative	negative	negative
7	negative	negative	negative
8	positive	positive	positive
9	positive	positive	positive
10	positive	positive	positive
11	positive	positive	positive
12	positive	positive	positive
13	positive	positive	positive
14	positive	positive	positive
15	positive	positive	positive
16	positive	negative	positive
17	negative	negative	negative
18	negative	negative	negative
19	positive	positive	positive
20	positive	positive	positive
21	positive	positive	positive
22	positive	positive	positive
23	negative	positive	positive
24	negative	positive	positive
25	negative	negative	negative
26	negative	negative	negative
27	negative	negative	negative
28	negative	negative	negative

29	negative	negative	negative
30	negative	negative	negative
31	negative	negative	negative
32	negative	negative	negative
33	negative	negative	negative
34	negative	positive	positive
35	positive	positive	negative
36	positive	positive	positive
37	positive	positive	positive
38	positive	positive	positive
39	negative	positive	positive
40	positive	positive	positive
41	positive	positive	positive
42	positive	positive	positive
43	positive	positive	positive
44	negative	positive	positive
45	negative	positive	positive
46	negative	negative	positive
47	positive	positive	positive
48	positive	positive	positive
49	positive	positive	positive
50	positive	positive	positive
51	positive	positive	positive
52	negative	positive	positive
53	positive	positive	positive
54	positive	positive	positive
55	negative	positive	positive
56	positive	positive	positive

Info: folgende heatmaps mit der Zielvariable result_ct39

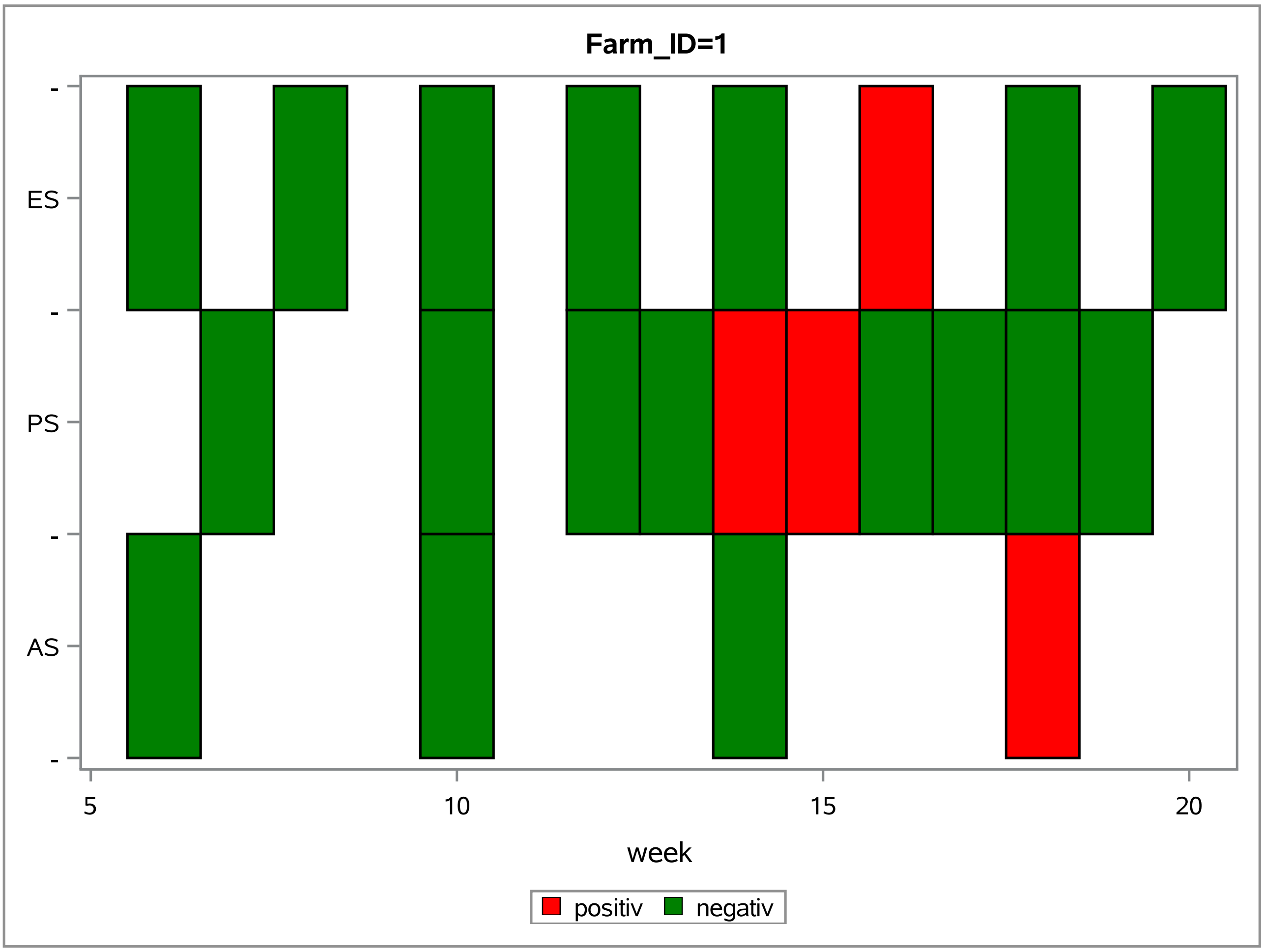
Farm_ID=1
ES
PS
AS
5
10
15
20
week
positiv
negativ

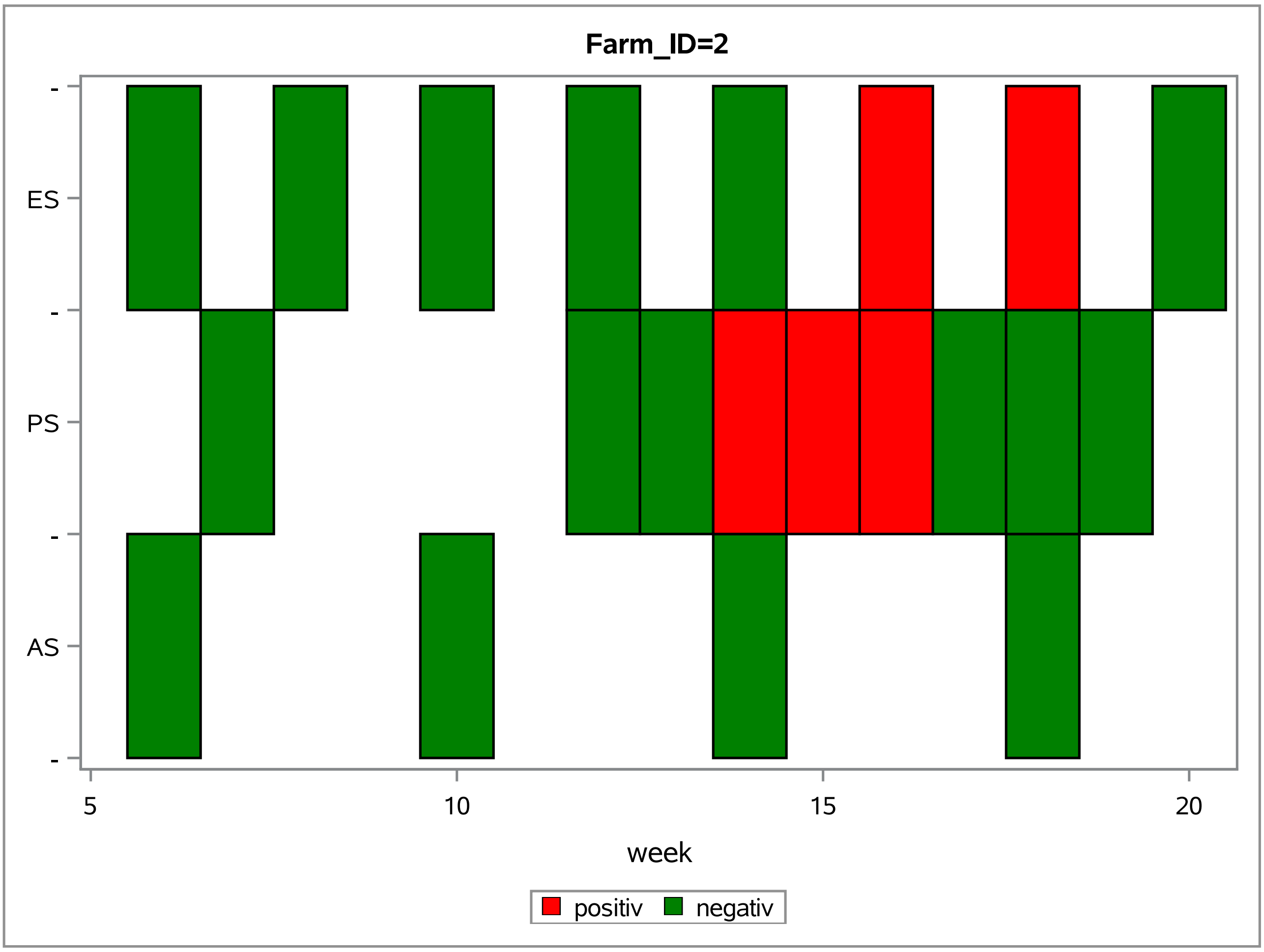
Farm_ID=2
ES
PS
AS
5
10
15
20
week
positiv
negativ

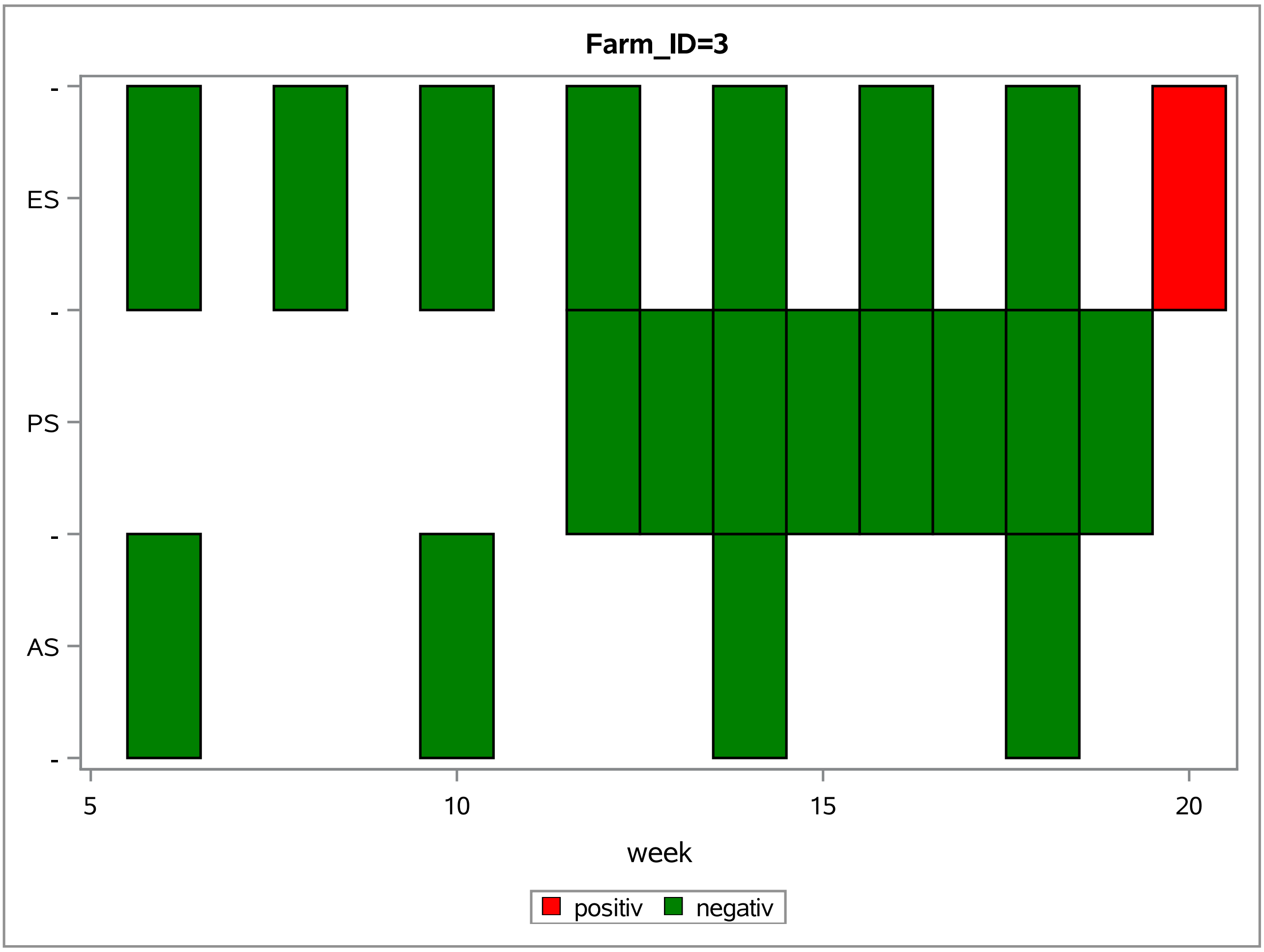
Farm_ID=3
ES
PS
AS
5
10
15
20
week
positiv
negativ

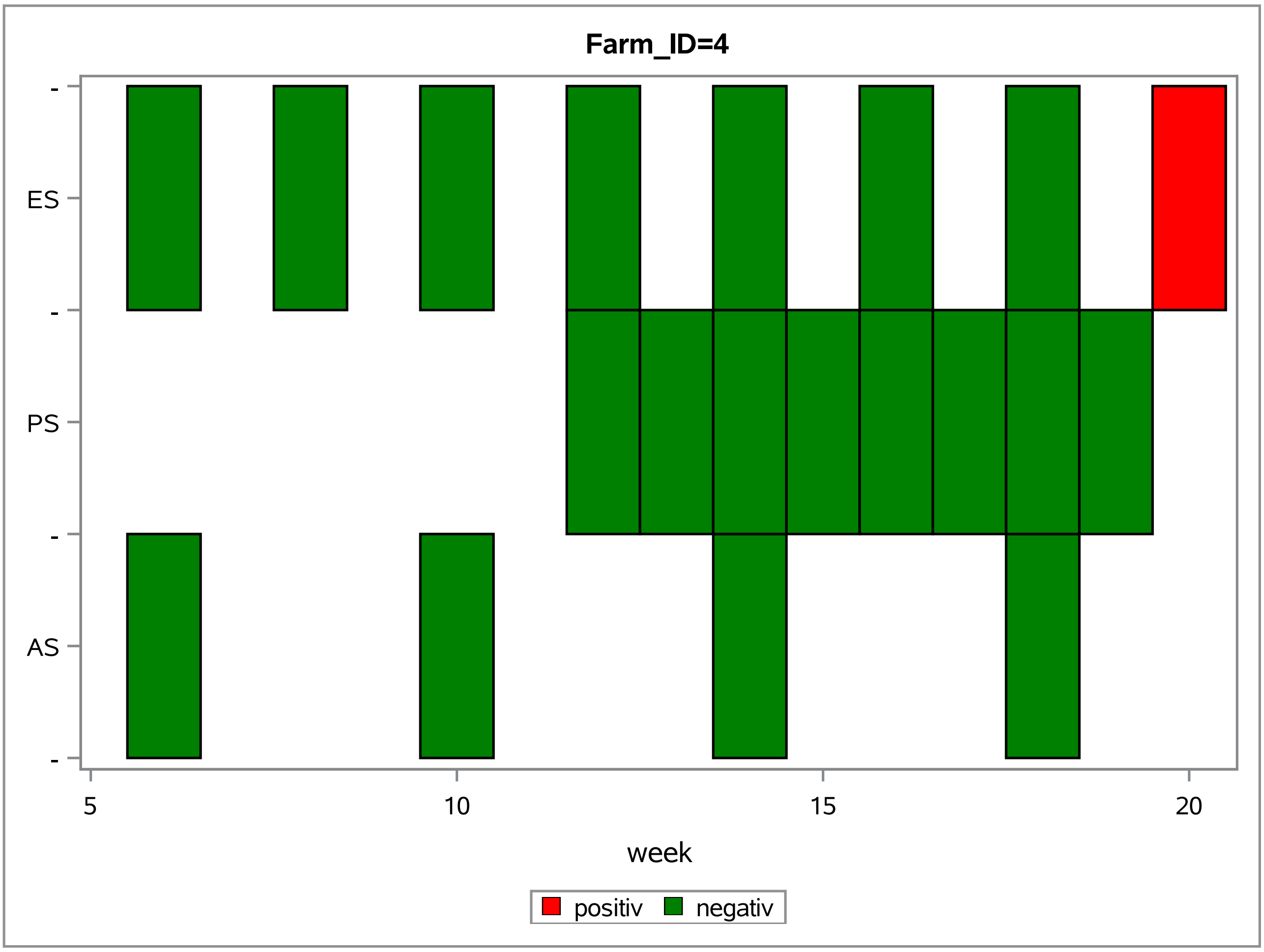

Farm_ID=4
-
ES
-
PS
-
AS
-
5
10
15
20
week
positiv
negativ

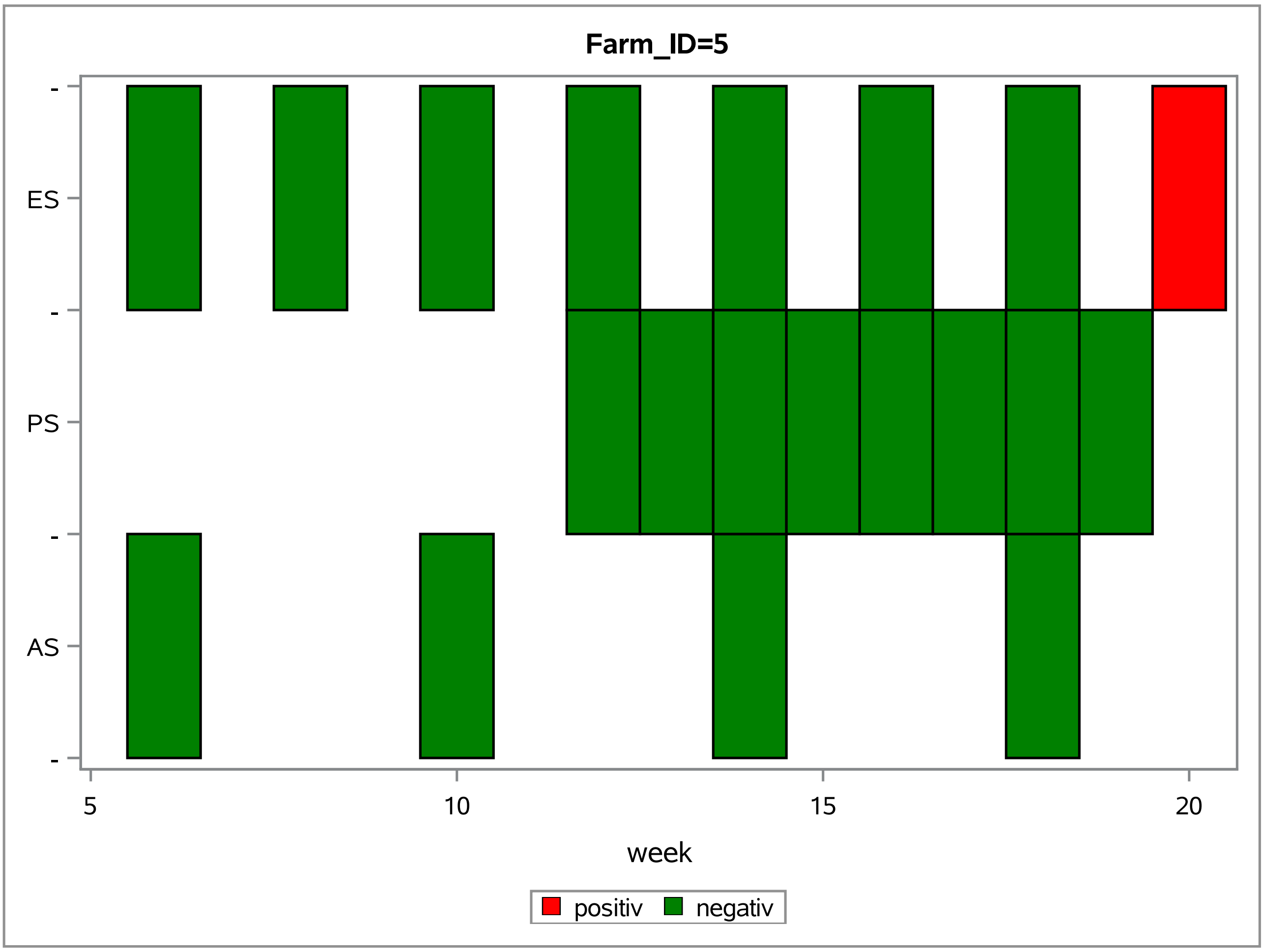
Farm_ID=5
ES
PS
AS
-
5
10
15
20
week
positiv
negativ

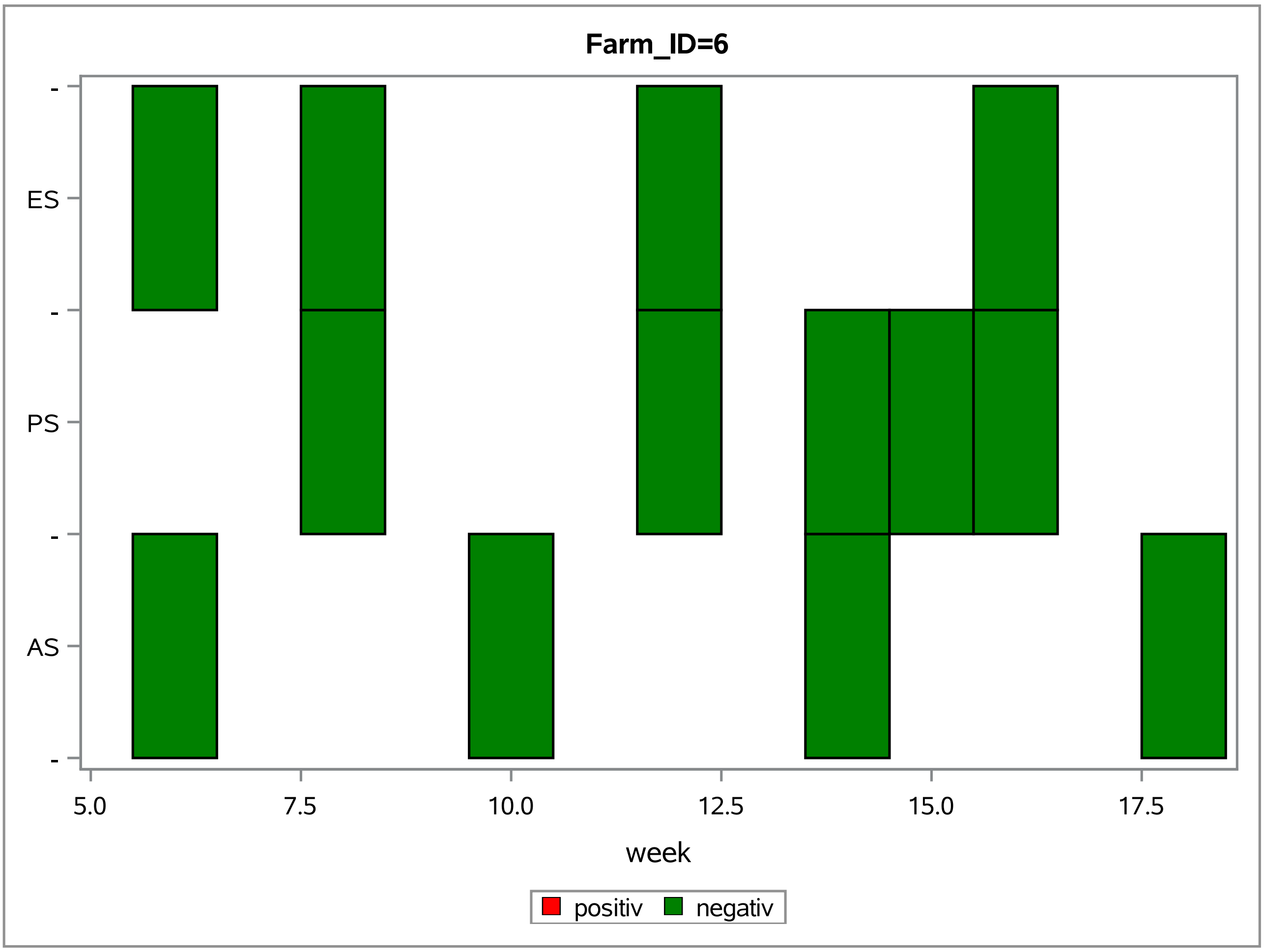
Farm_ID=6
ES
PS
AS
5.0
7.5
10.0
12.5
15.0
17.5
week
positiv
negativ

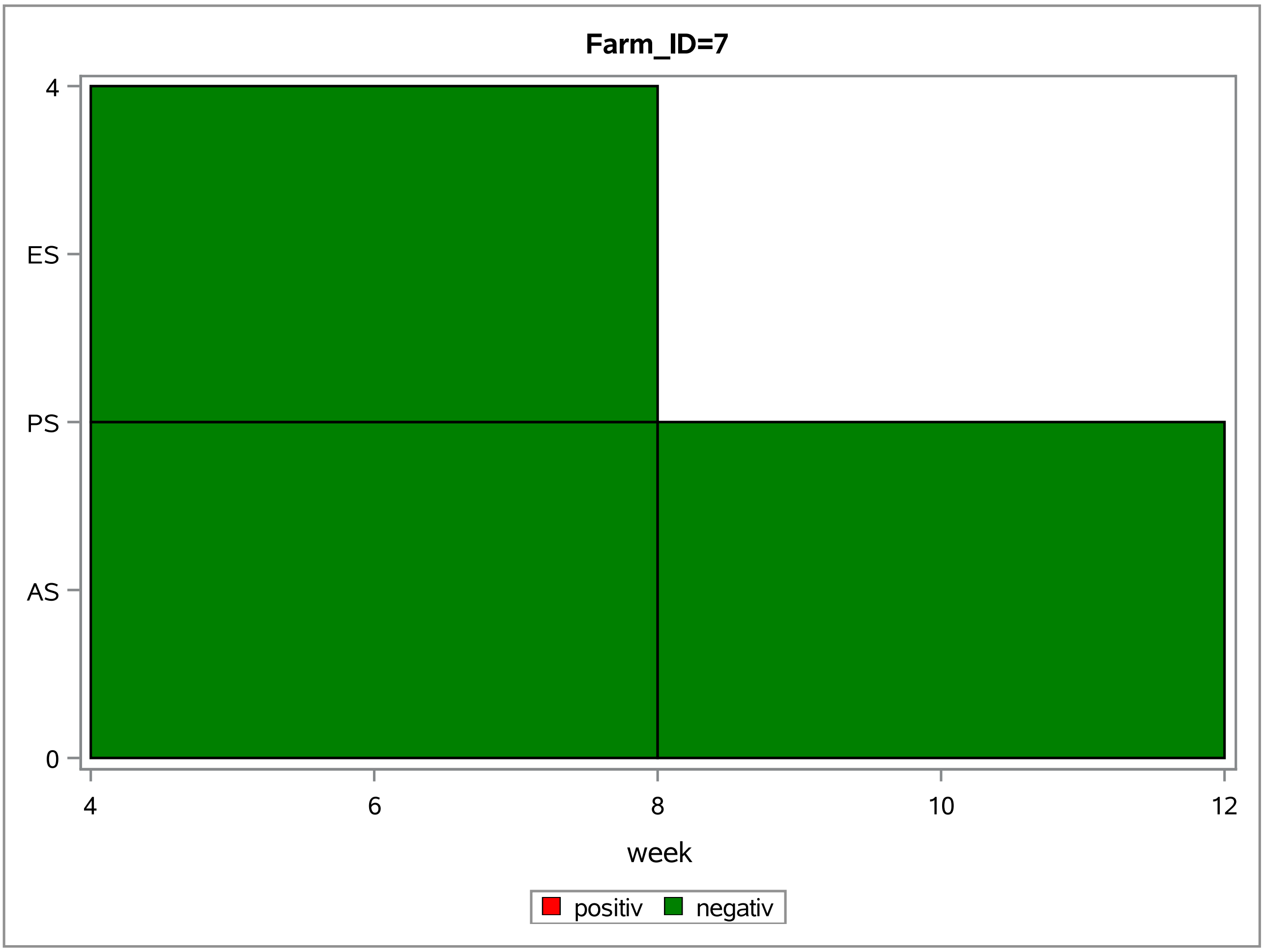
Farm_ID=7
4
ES
PS
AS
0
4
6
8
10
12
week
positiv
negativ

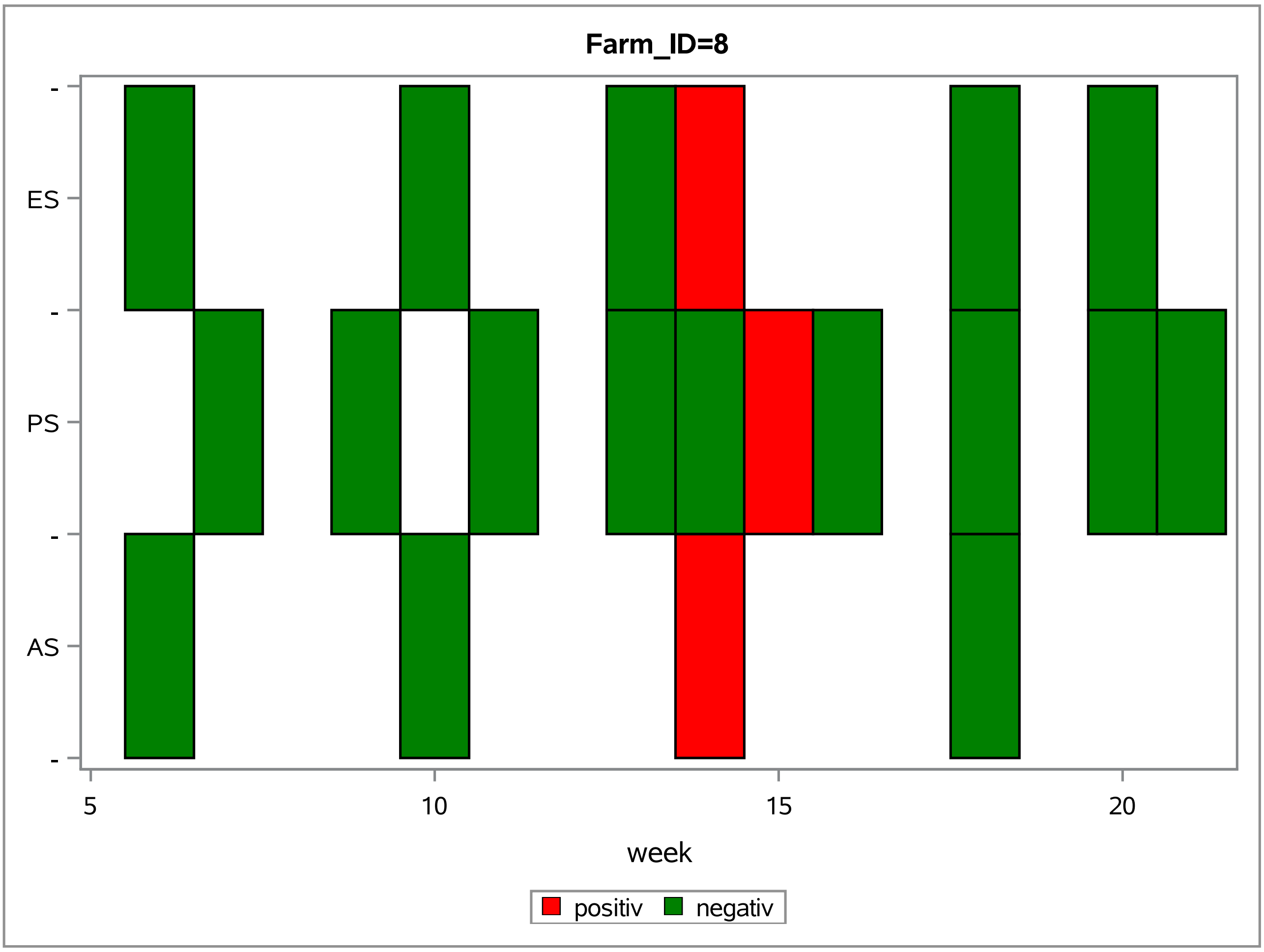
Farm_ID=8
ES
PS
AS
5
10
15
20
week
positiv
negativ

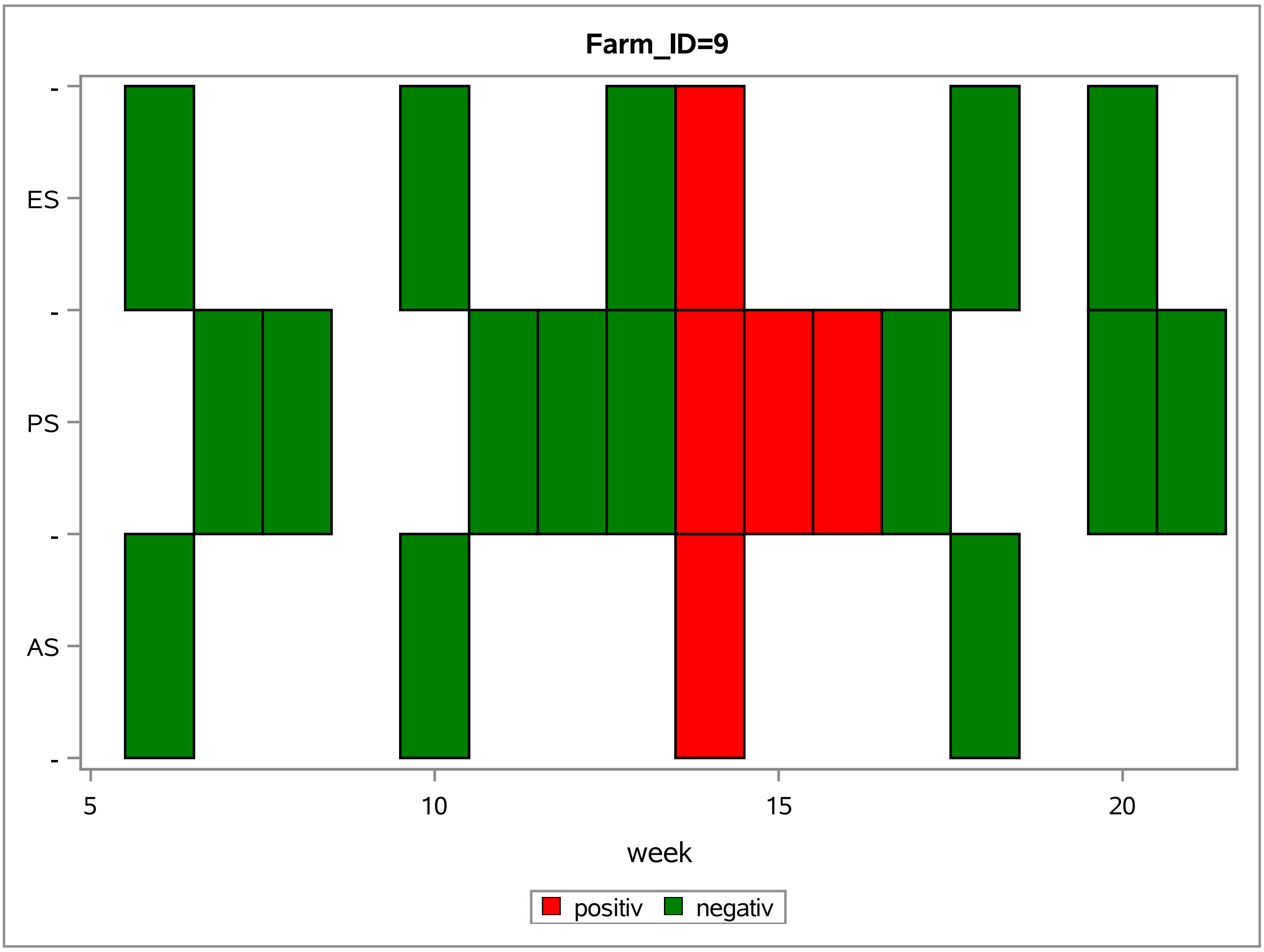

Farm_ID=9
ES
PS
AS
5
10
15
20
week
positiv
negativ

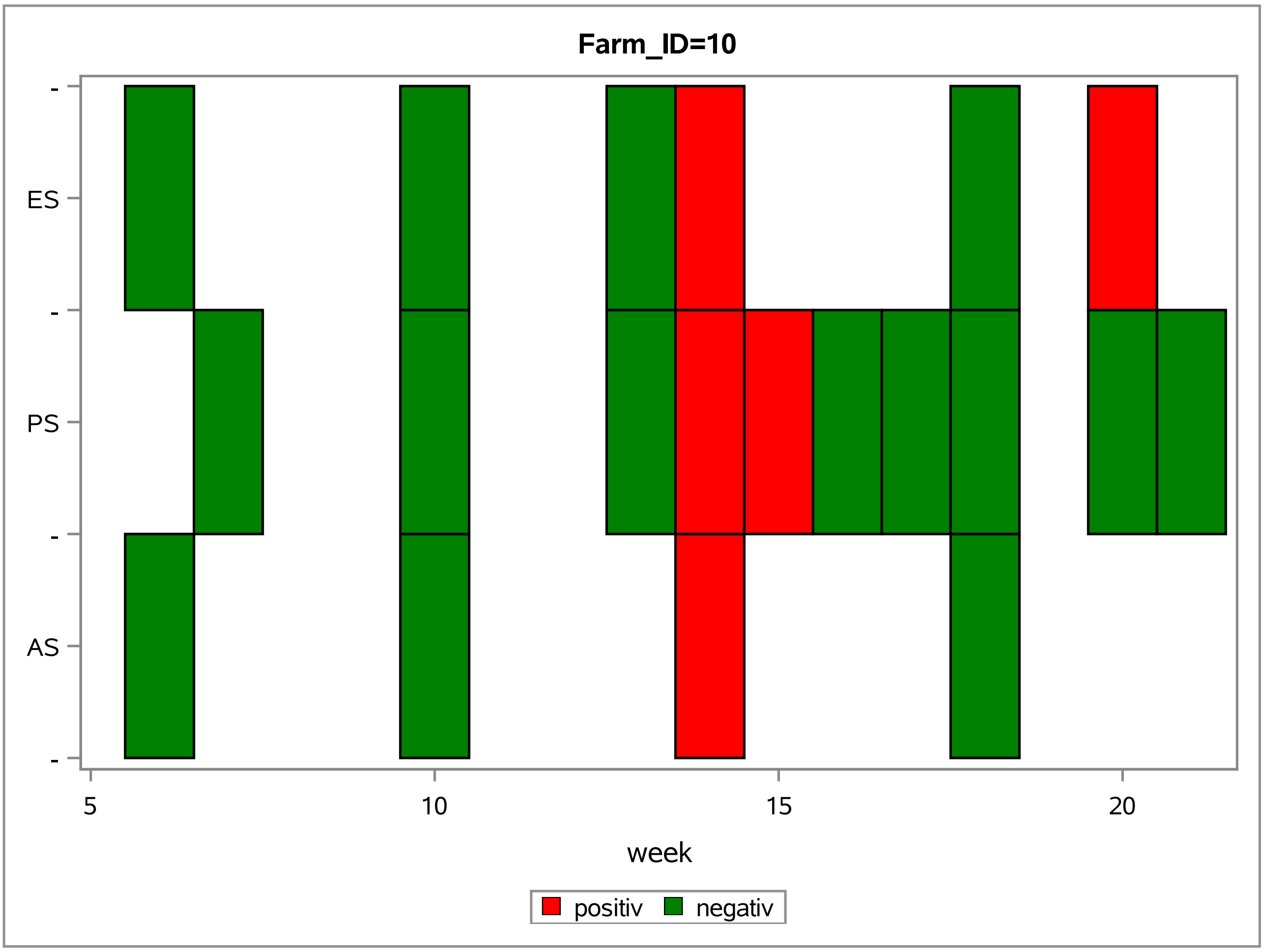
Farm_ID=10
-
ES
-
PS
-
AS
-
5
10
15
20
week
positiv
negativ

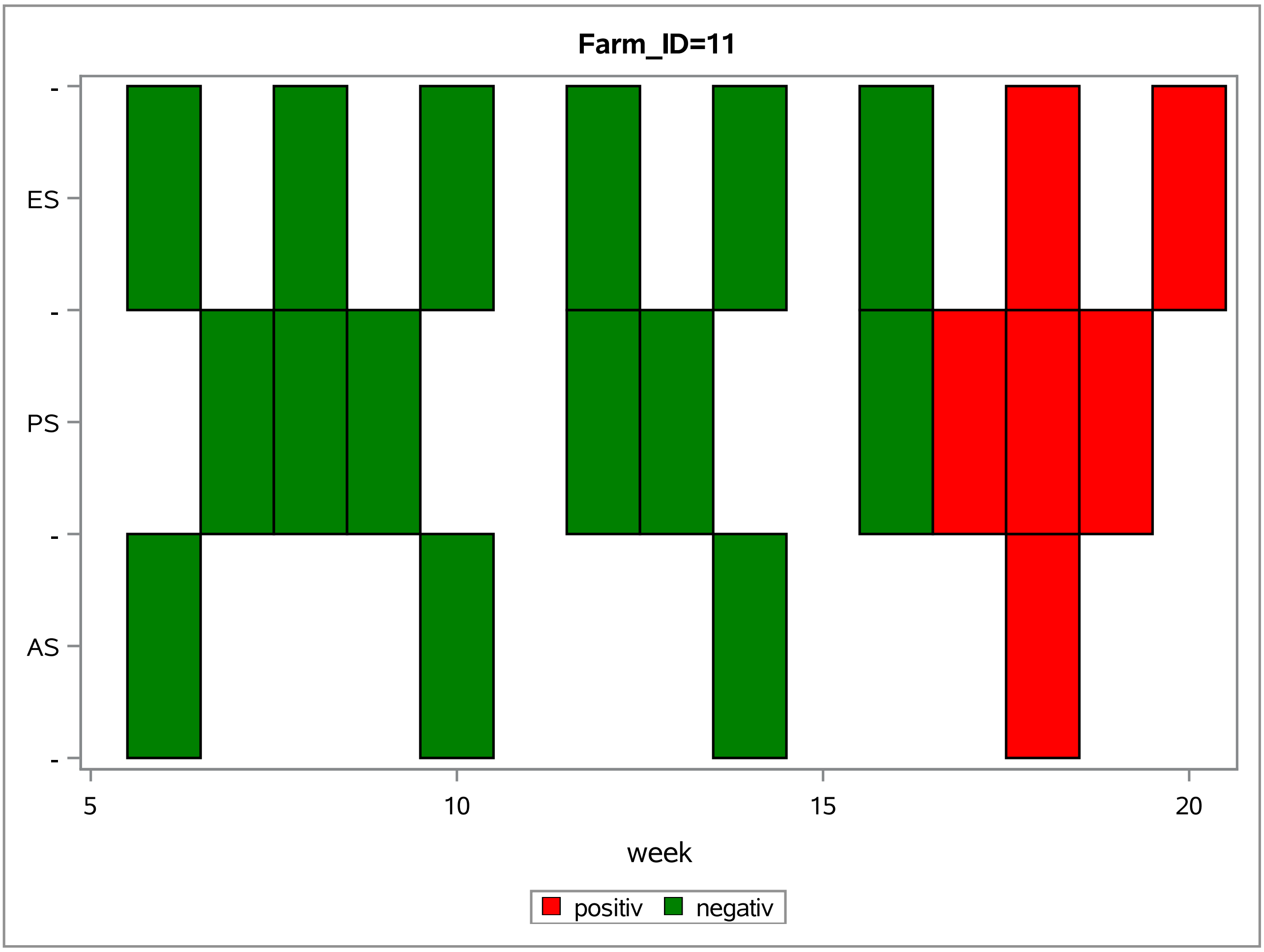
Farm_ID=11
ES
PS
AS
5
10
15
20
week
positiv
negativ

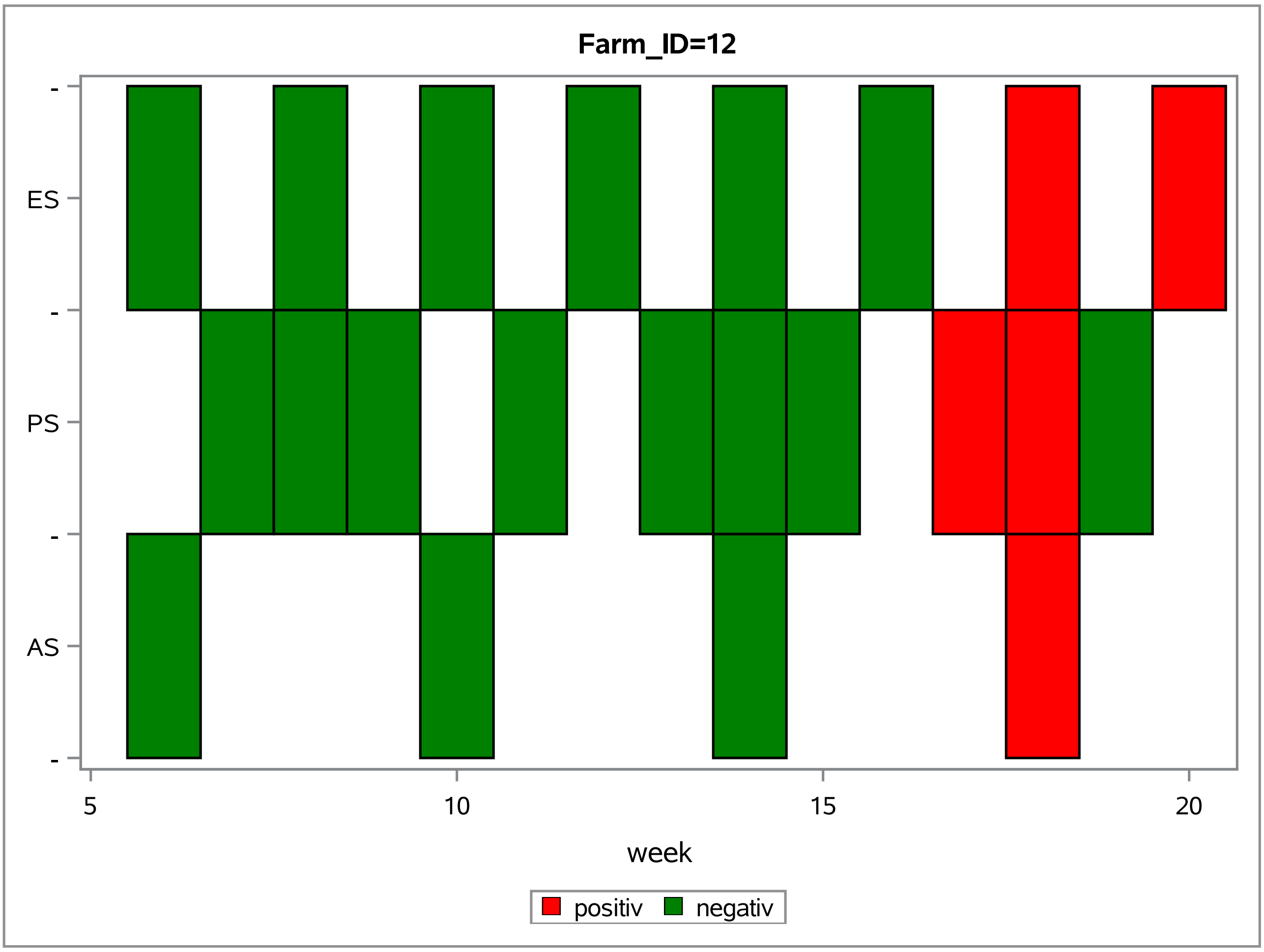
Farm_ID=12
ES
PS
AS
5
10
15
20
week
positiv
negativ

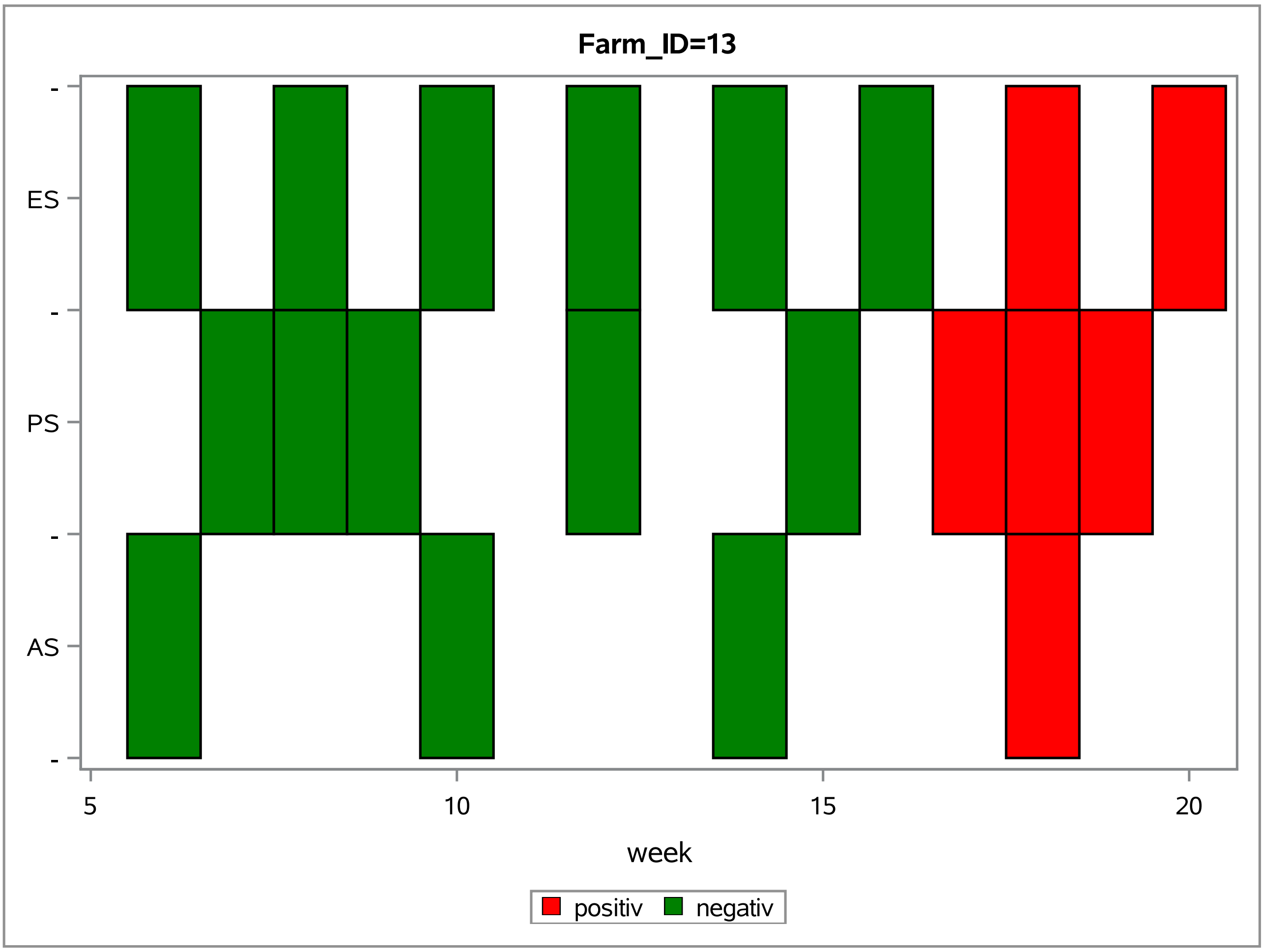
Farm_ID=13
ES
PS
AS
5
10
15
20
week
positiv
negativ

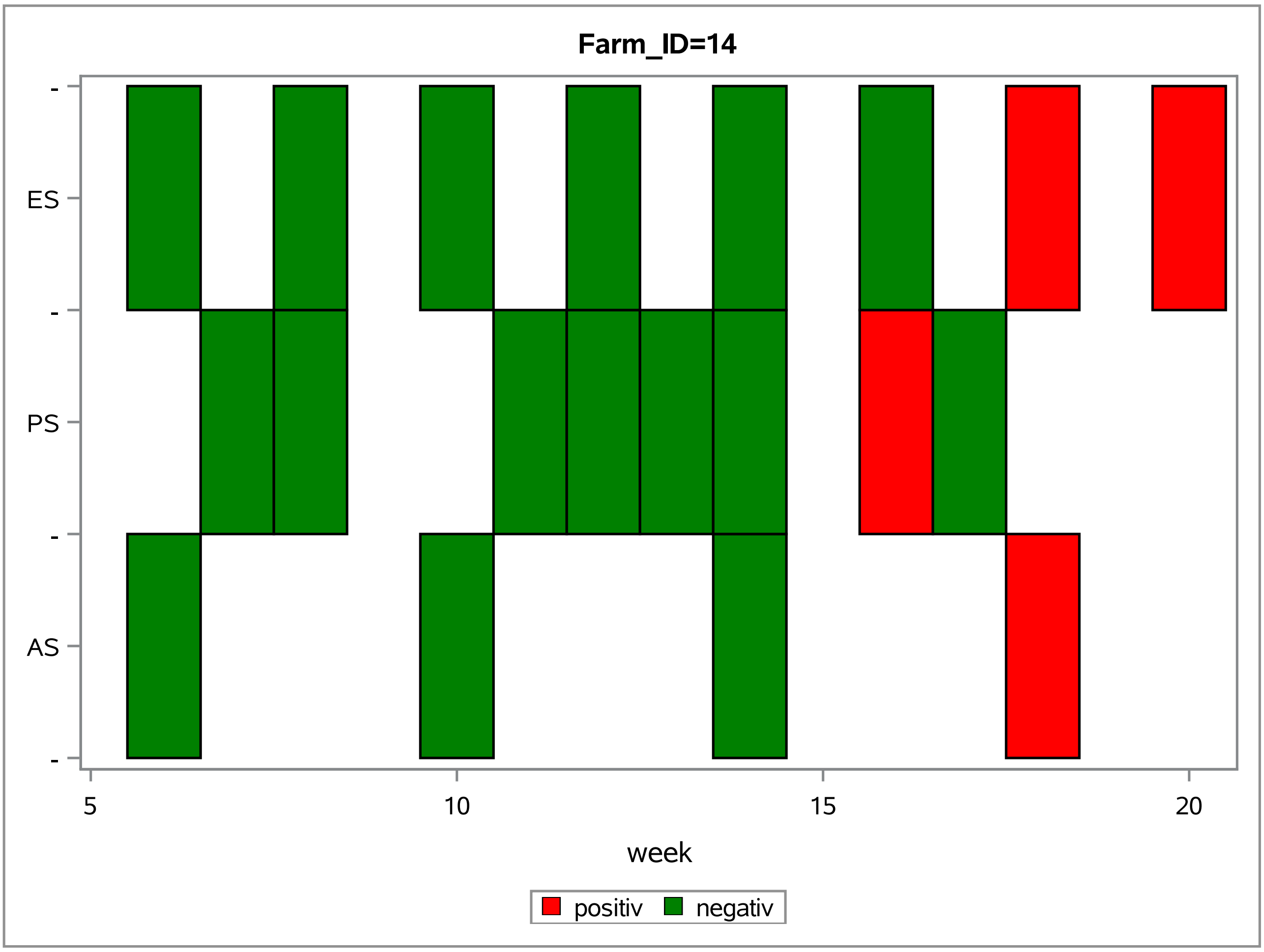
Farm_ID=14
ES
PS
AS
5
10
15
20
week
positiv
negativ

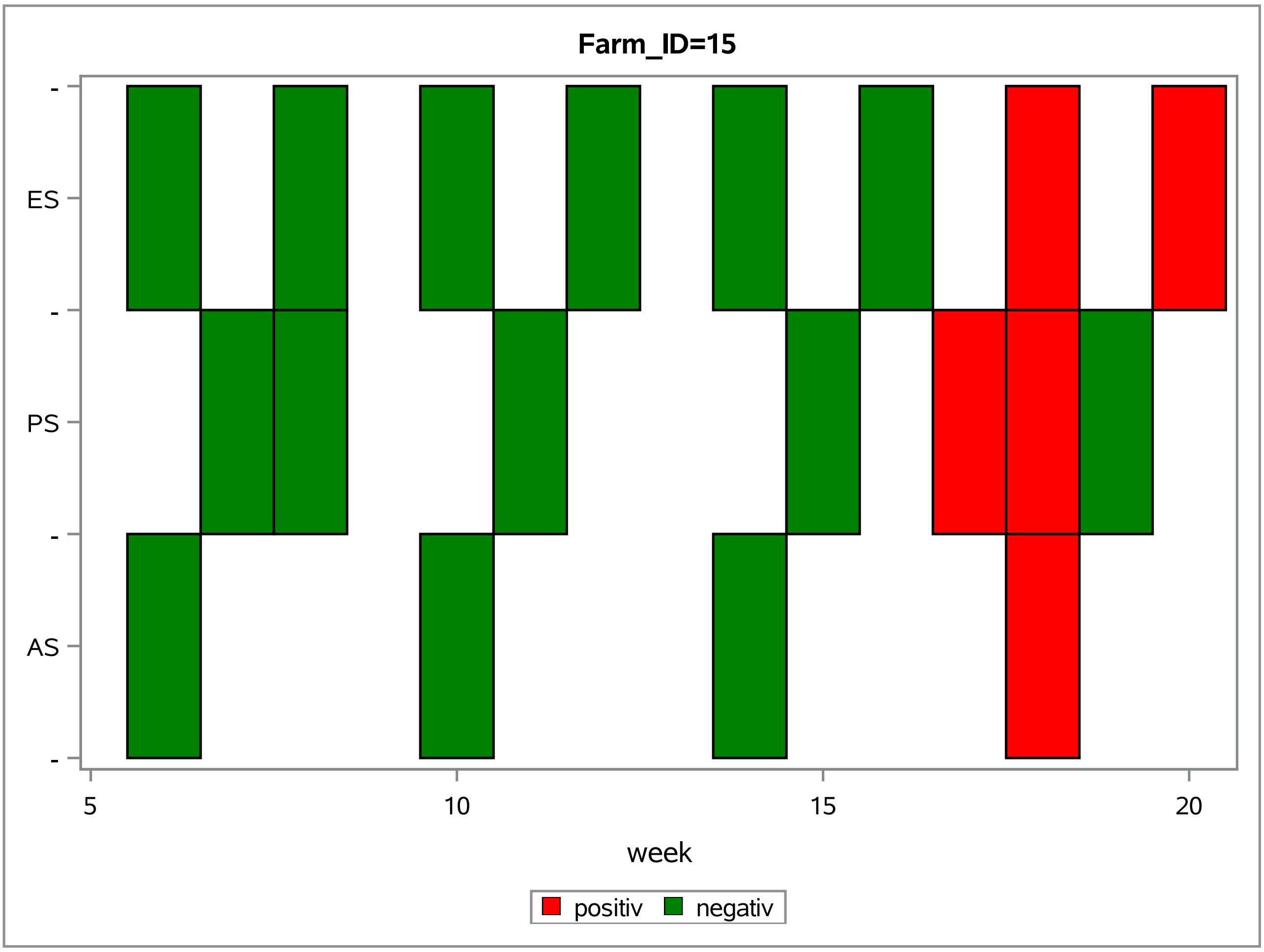
Farm_ID=15
ES
PS
AS
5
10
15
20
week
positiv
negativ

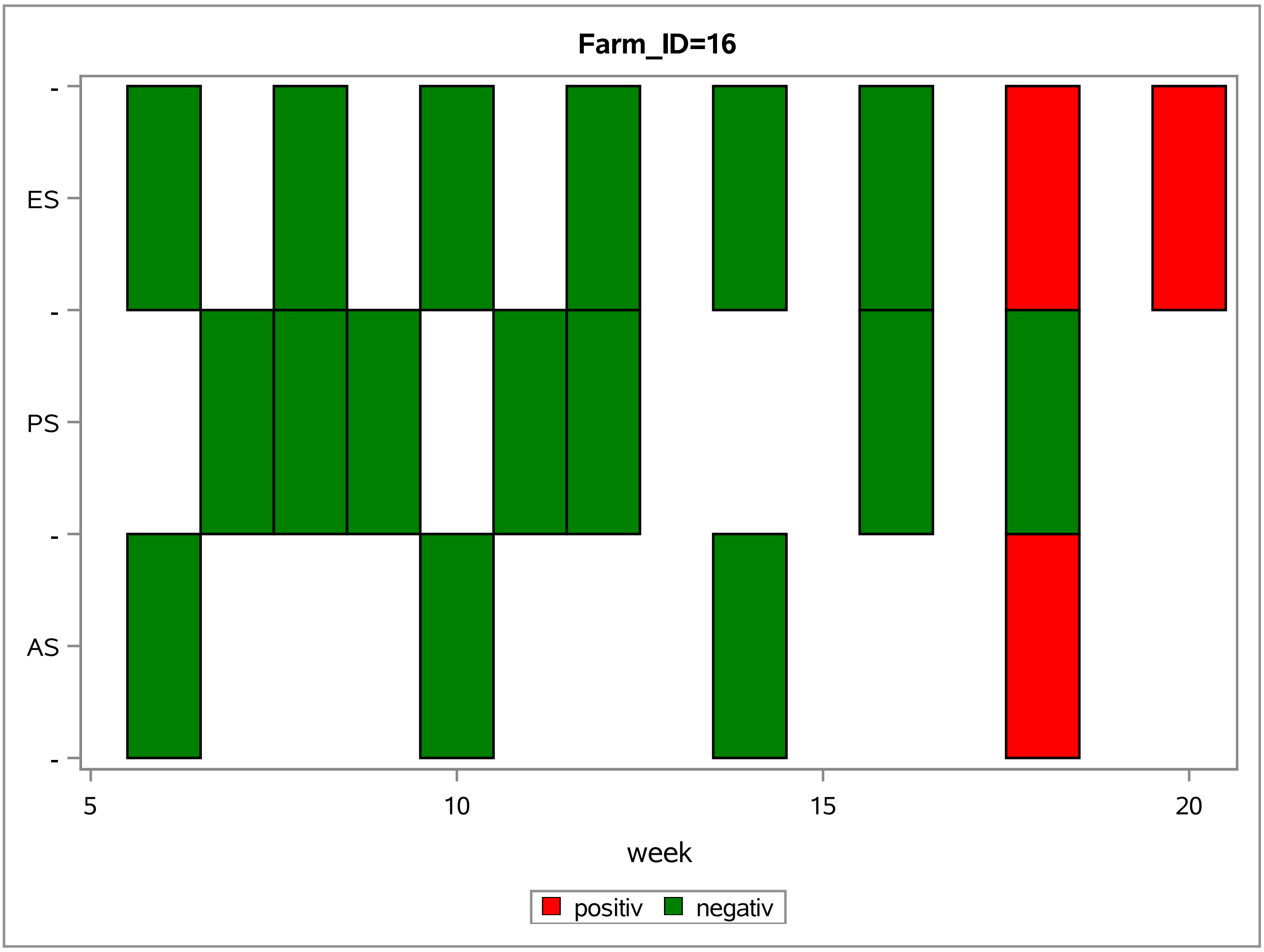
Farm_ID=16
ES
PS
AS
5
10
15
20
week
positiv
negativ

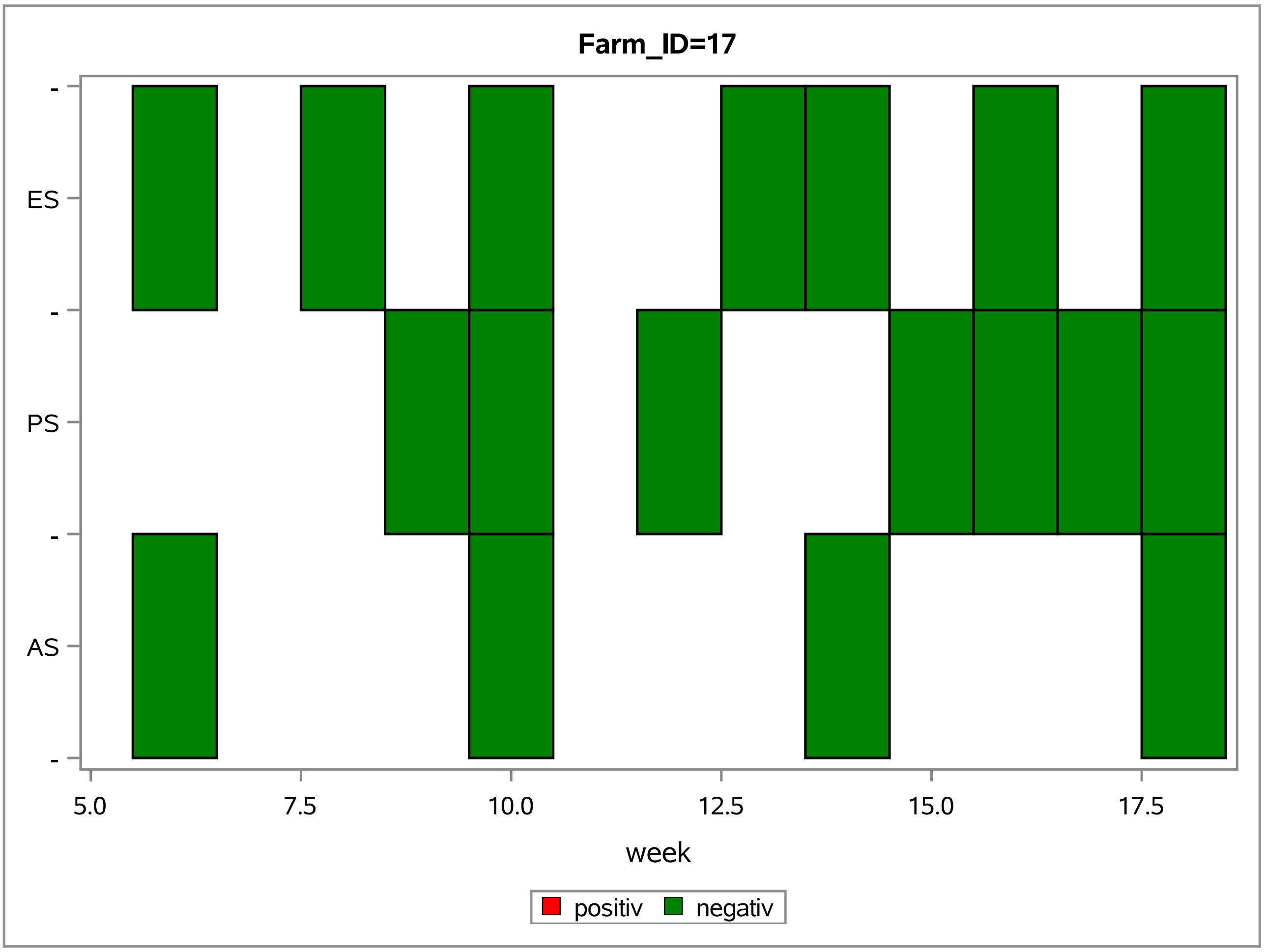
Farm_ID=17
-
ES
-
PS
-
AS
-
5.0
7.5
10.0
12.5
15.0
17.5
week
positiv
negativ

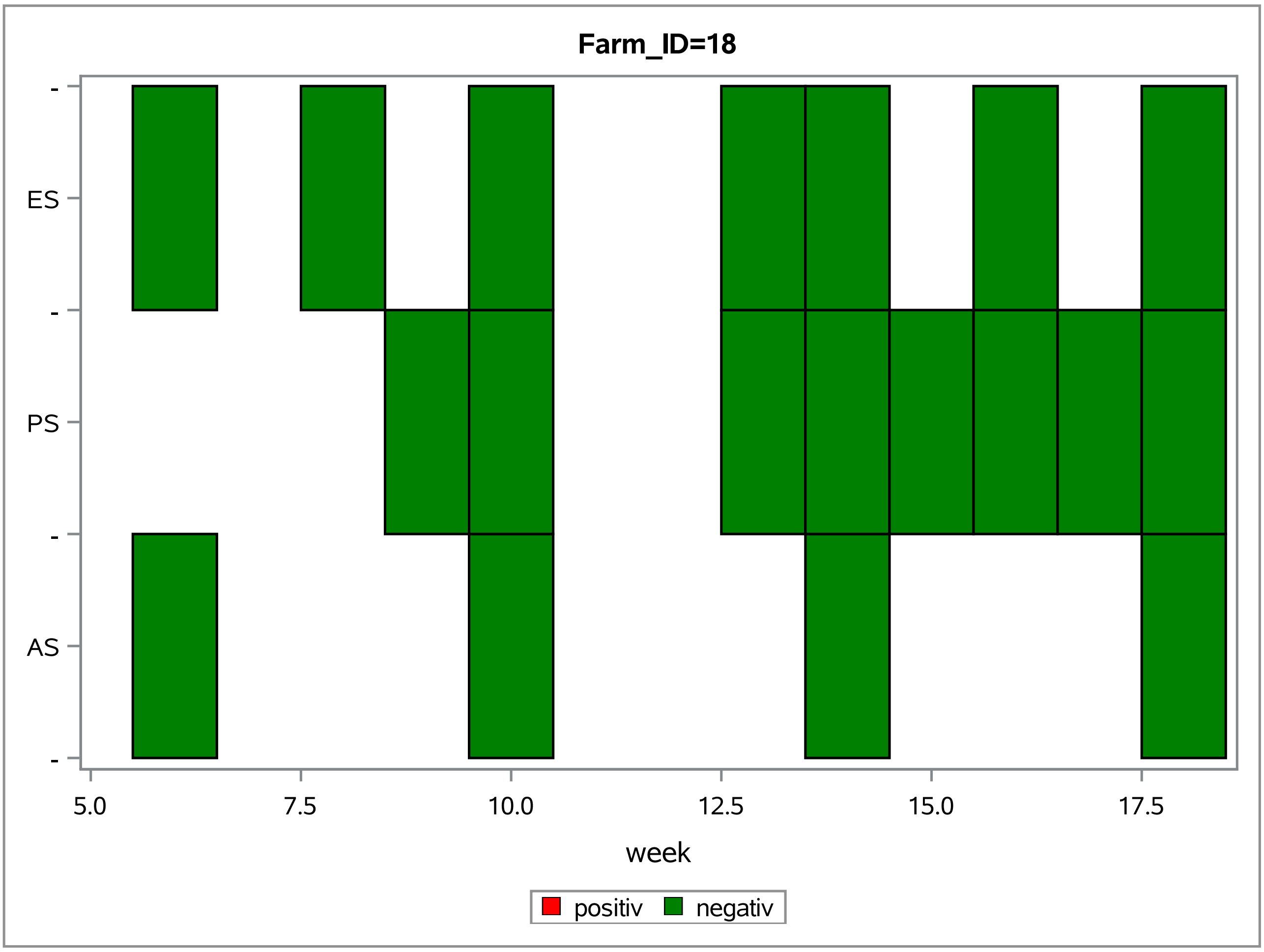
Farm_ID=18
ES
PS
AS
5.0
7.5
10.0
12.5
15.0
17.5
week
positiv
negativ

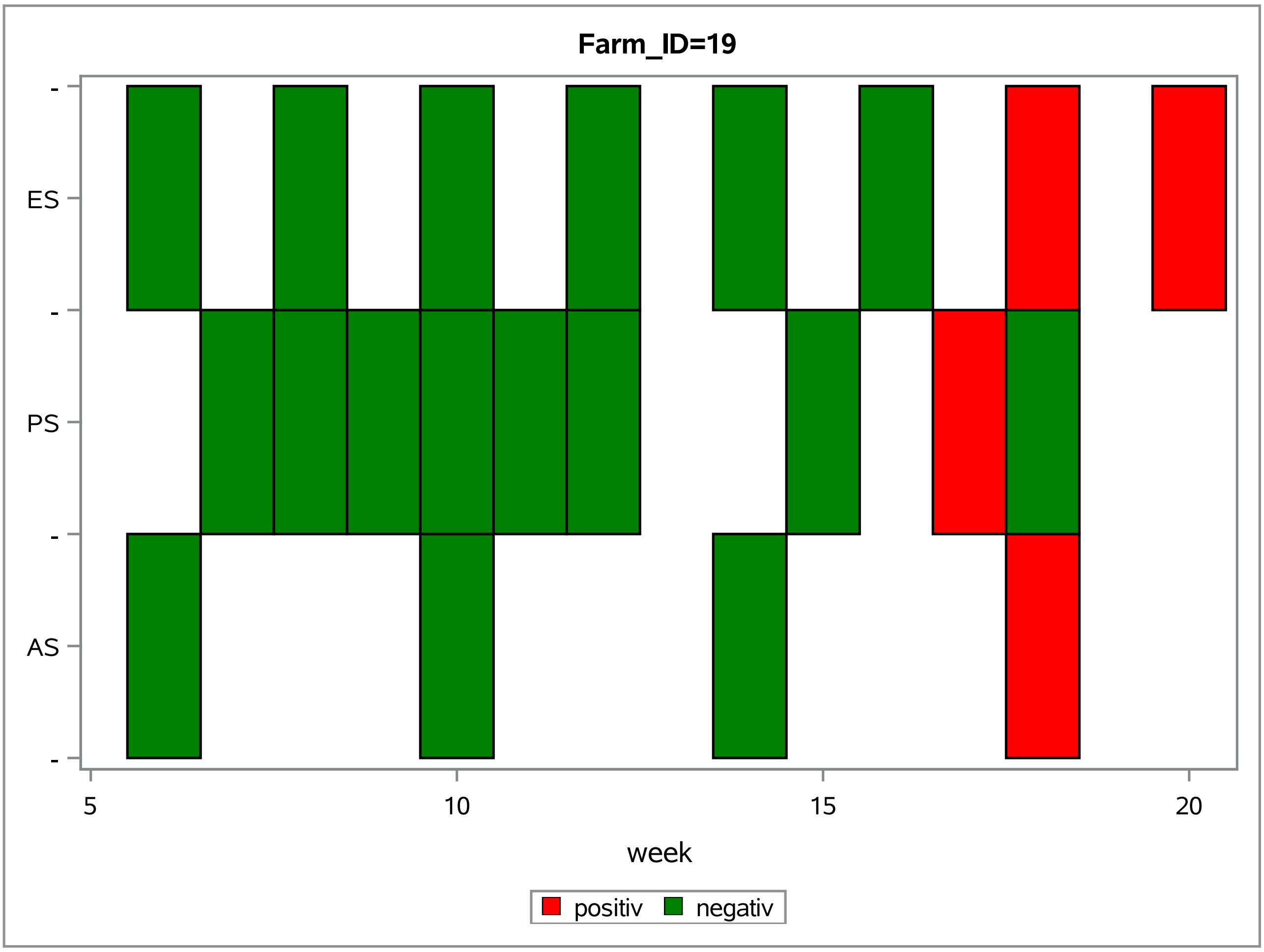
Farm_ID=19
ES
PS
AS
5
10
15
20
week
positiv
negativ

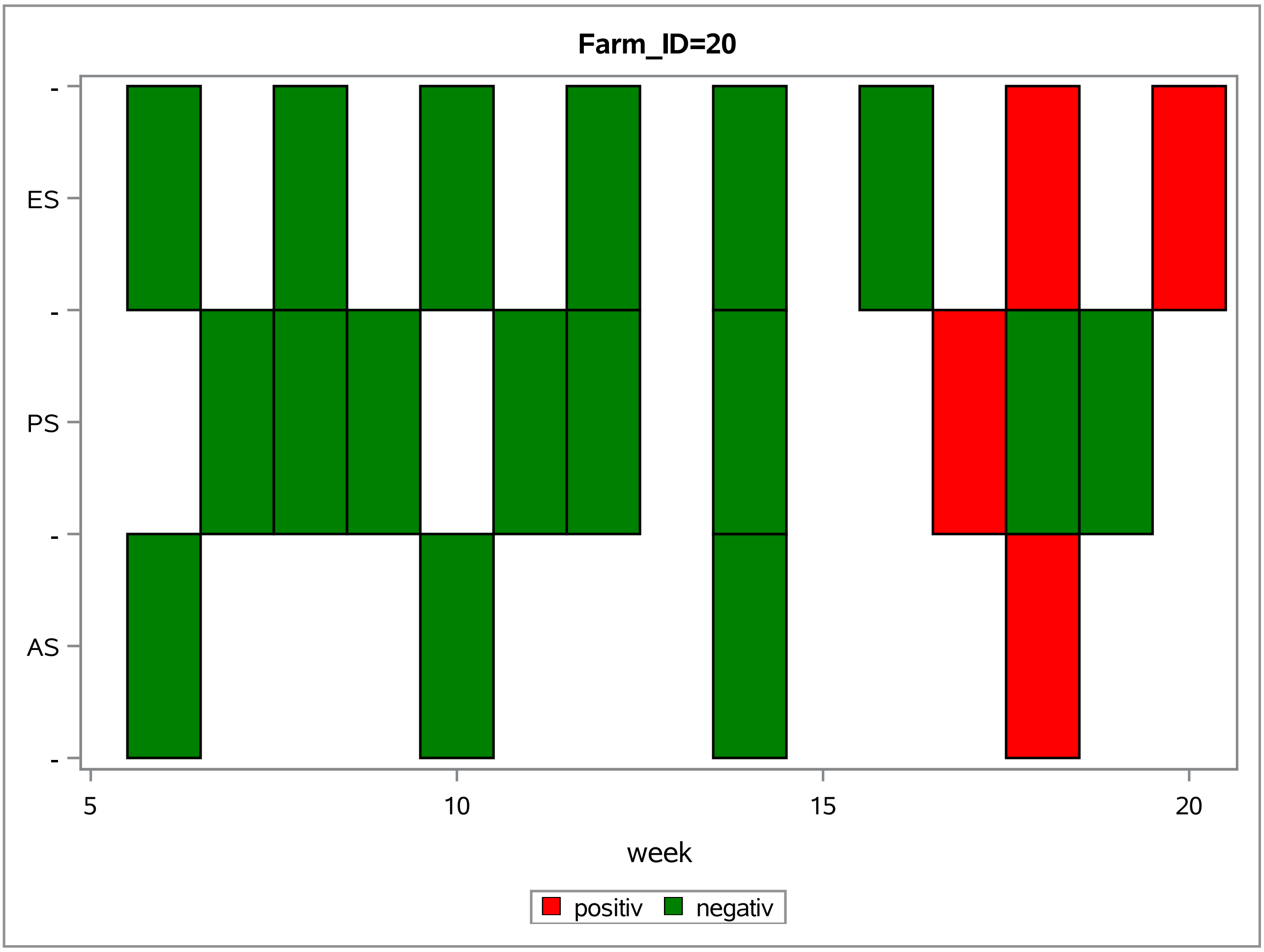
Farm_ID=20
ES
PS
AS
5
10
15
20
week
positiv
negativ

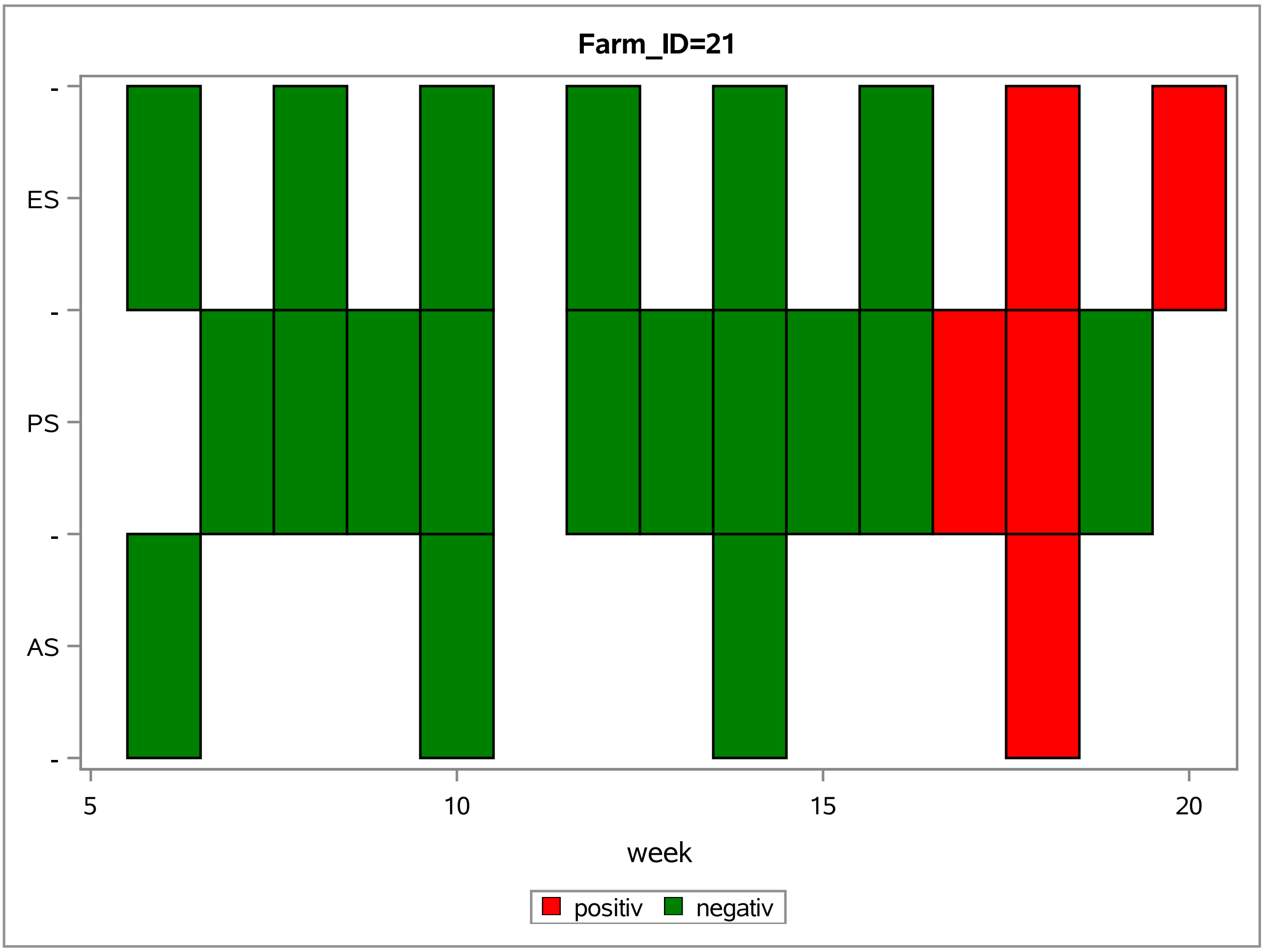
Farm_ID=21
ES
PS
AS
5
10
15
20
week
positiv
negativ

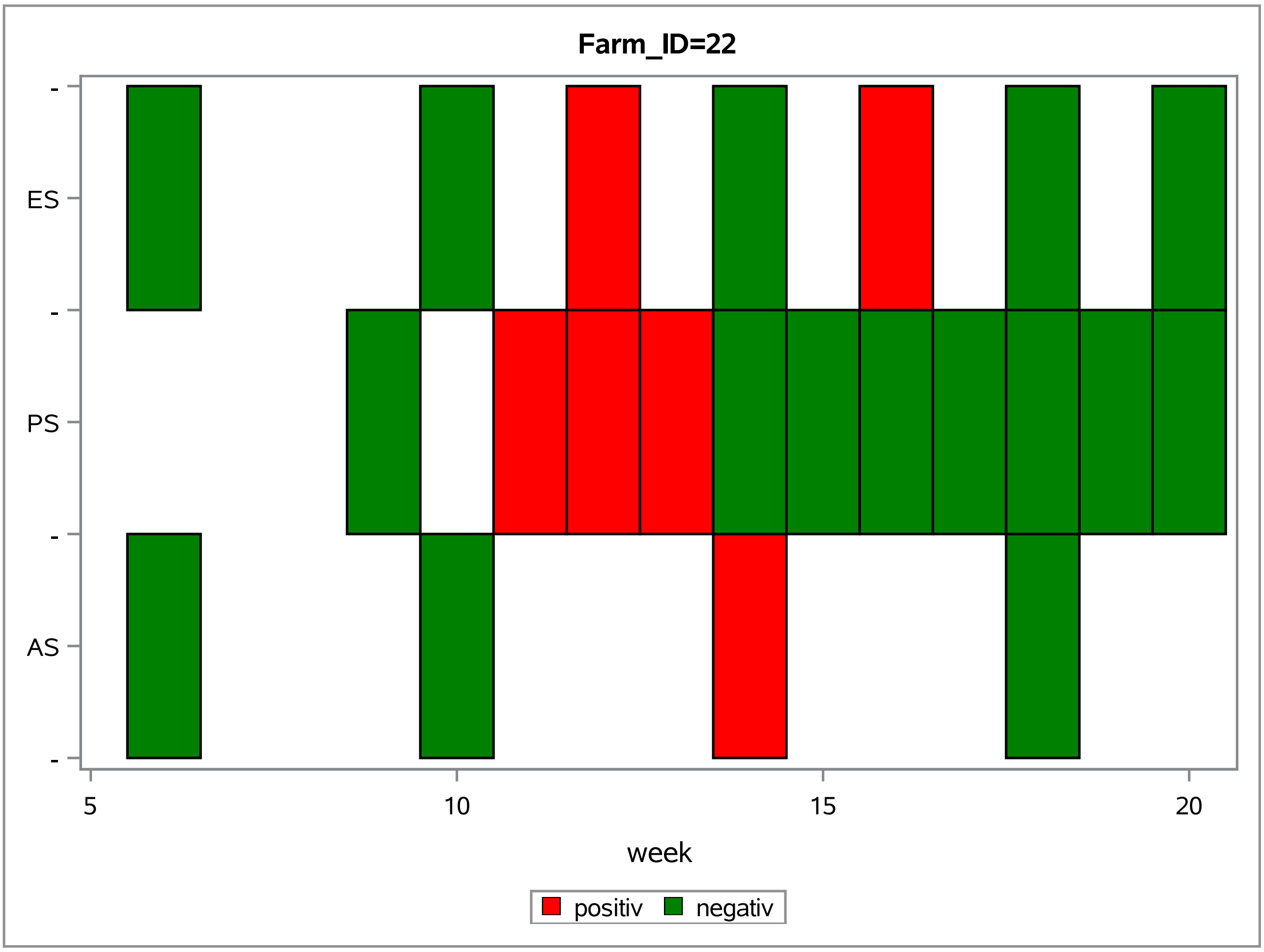
Farm_ID=22
ES
PS
AS
5
10
15
20
week
positiv
negativ

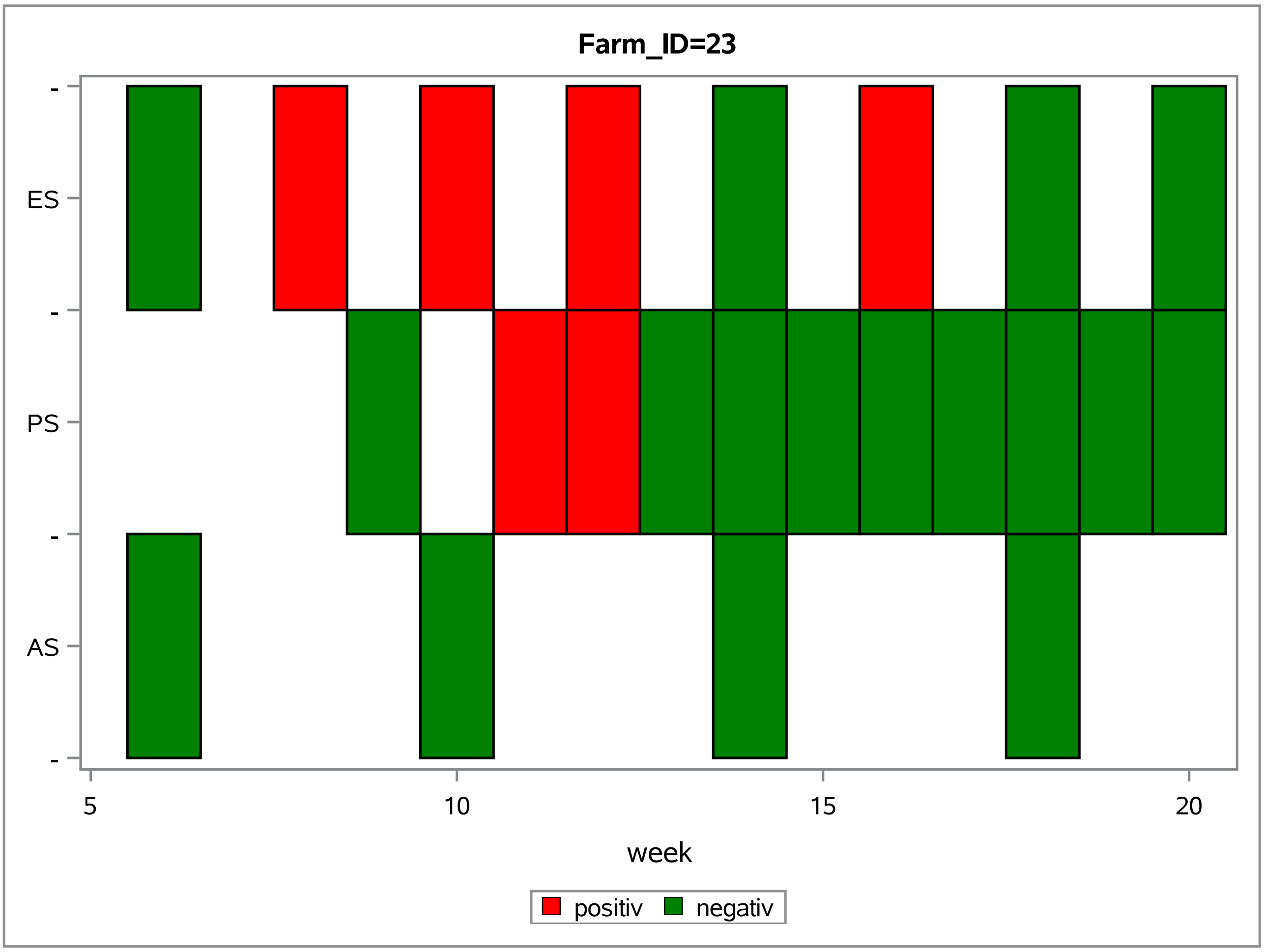
Farm_ID=23
ES
PS
AS
5
10
15
20
week
positiv
negativ

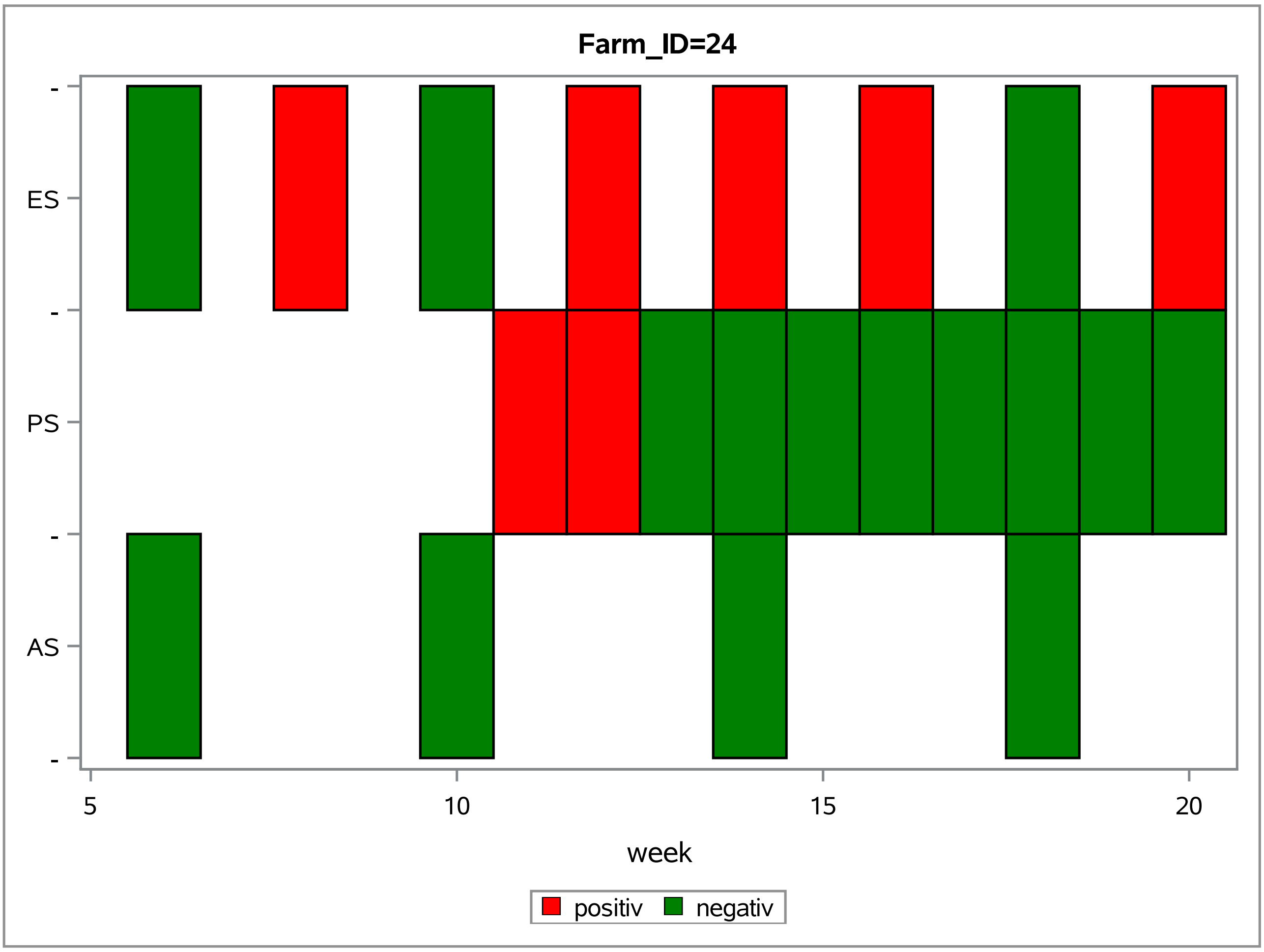
Farm_ID=24
ES
PS
AS
5
10
15
20
week
positiv
negativ

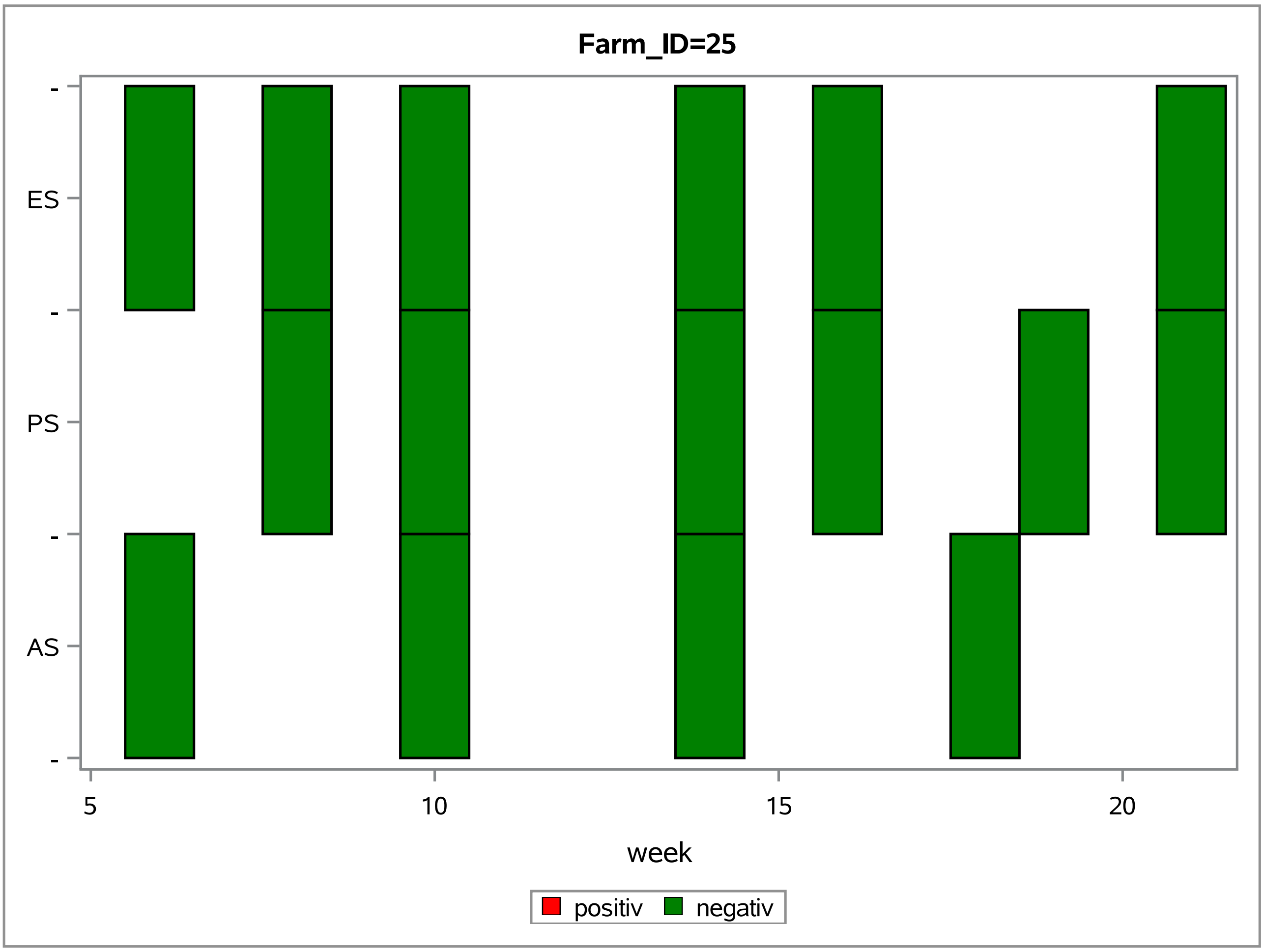
Farm_ID=25
ES
PS
AS
5
10
15
20
week
positiv
negativ

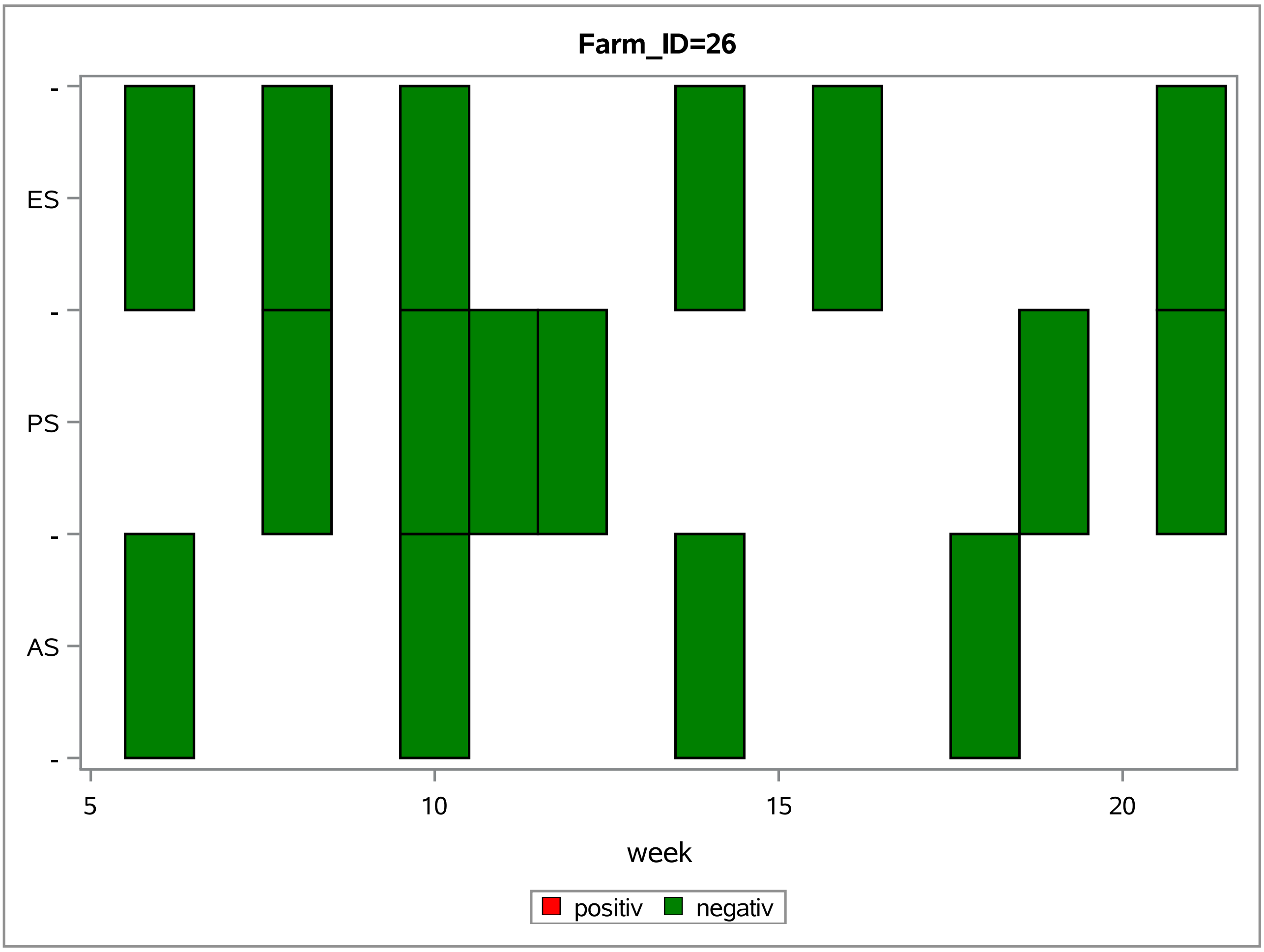
Farm_ID=26
ES
PS
AS
5
10
15
20
week
positiv
negativ

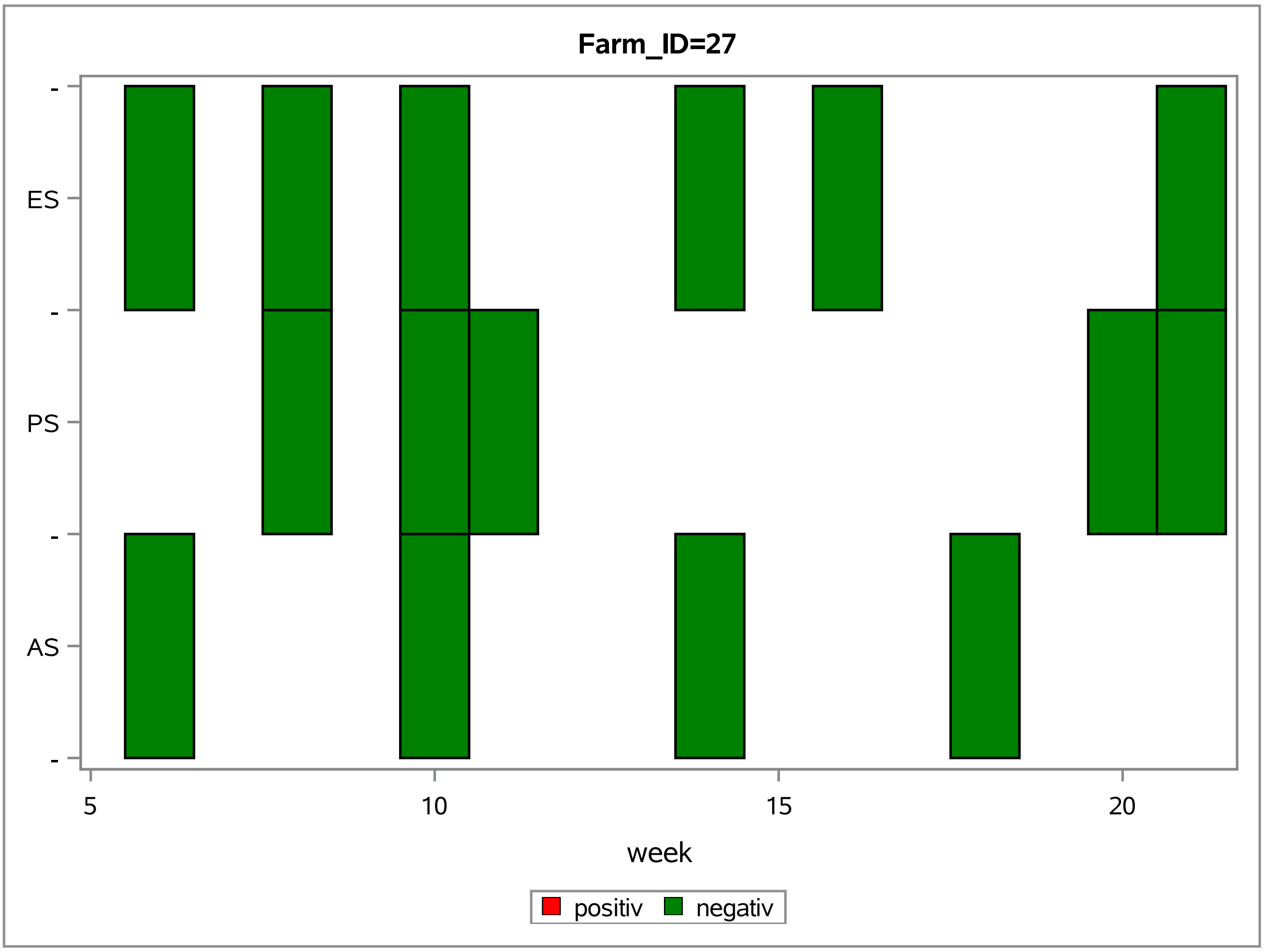
Farm_ID=27
ES
PS
AS
5
10
15
20
week
positiv
negativ

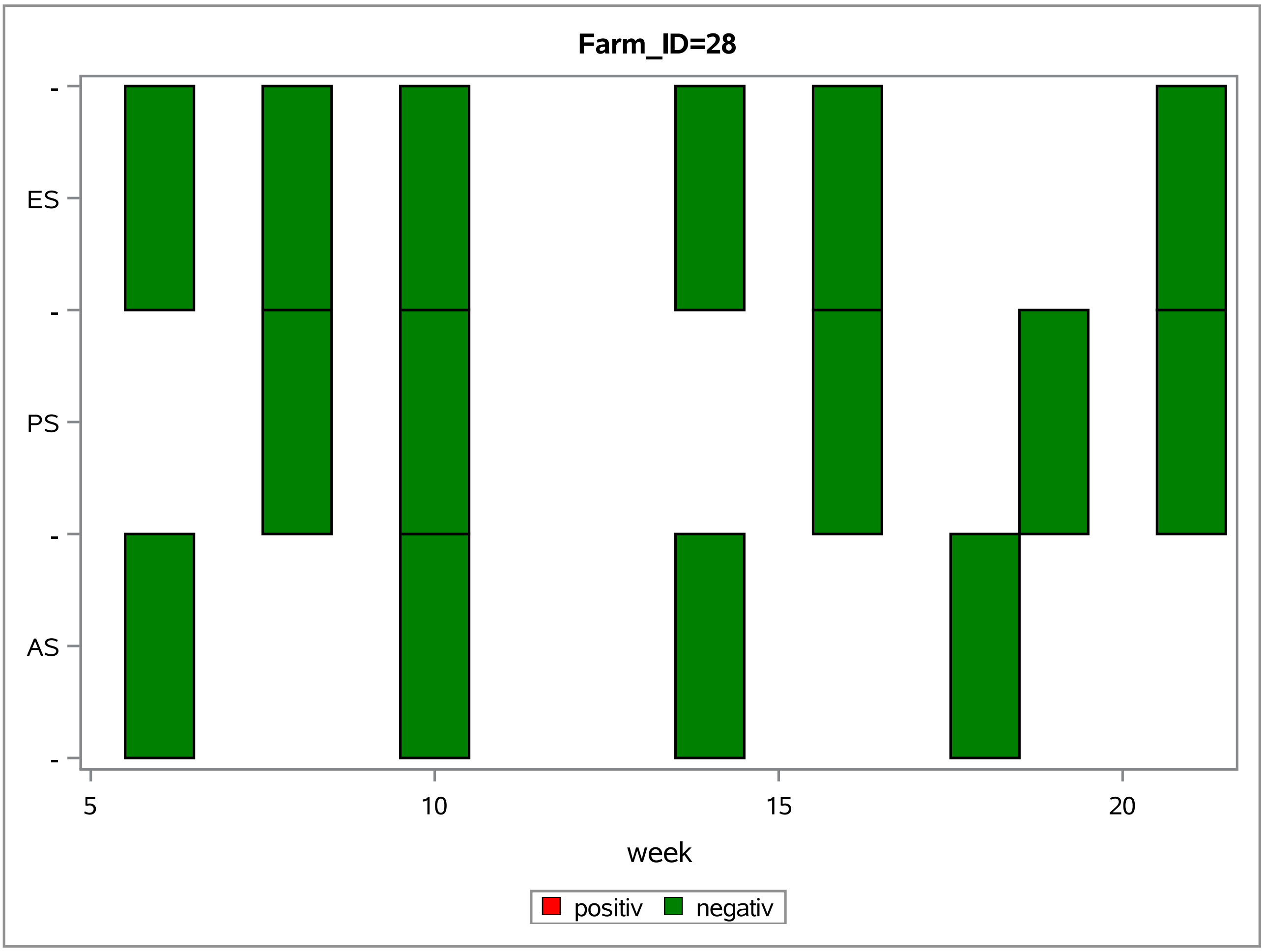
Farm_ID=28
ES
PS
AS
5
10
15
20
week
positiv
negativ

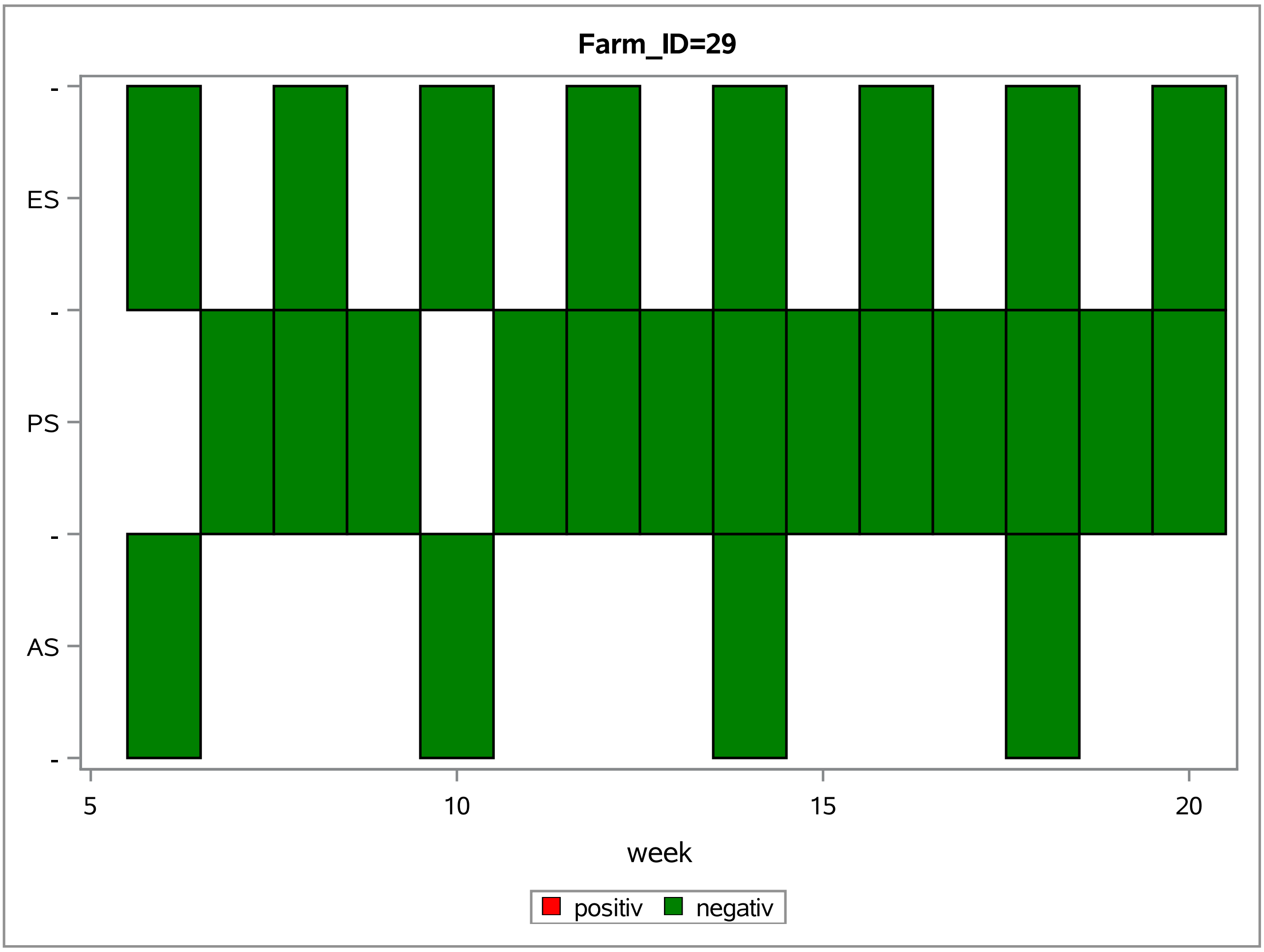

Farm_ID=29
ES
PS
AS
5
10
15
20
week
positiv
negativ

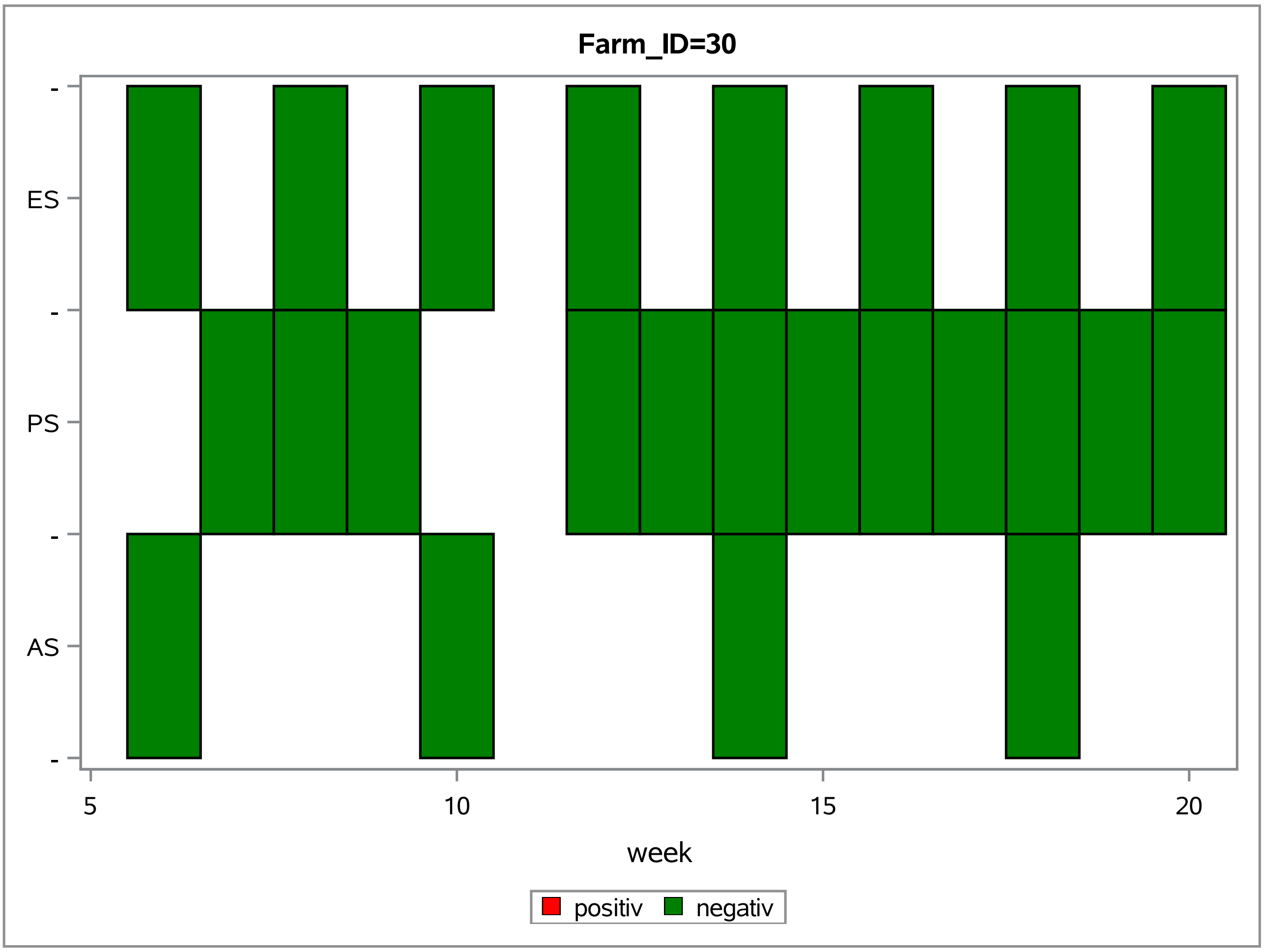
Farm_ID=30
ES
PS
AS
5
10
15
20
week
positiv
negativ

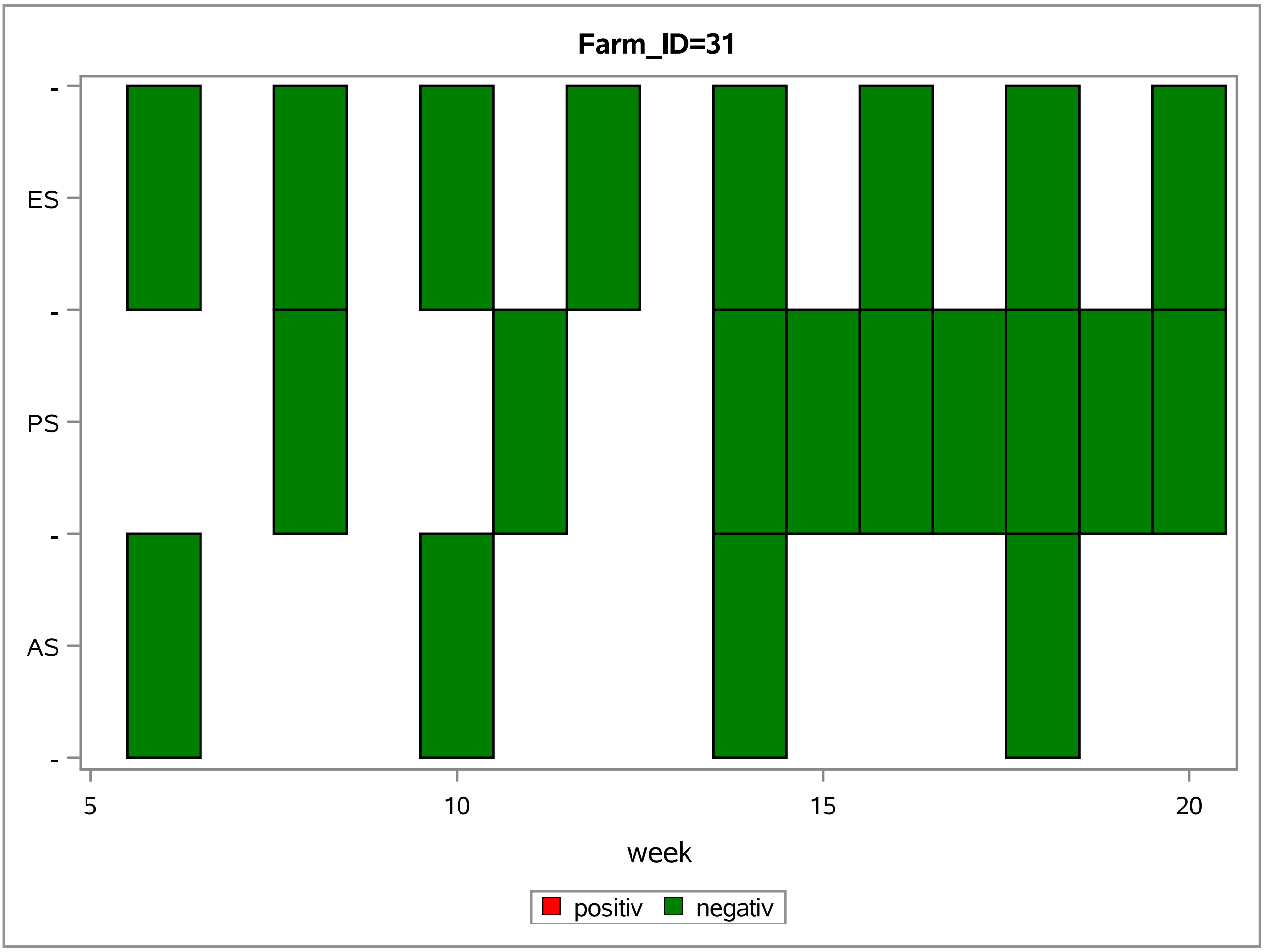
Farm_ID=31
ES
PS
AS
5
10
15
20
week
positiv
negativ

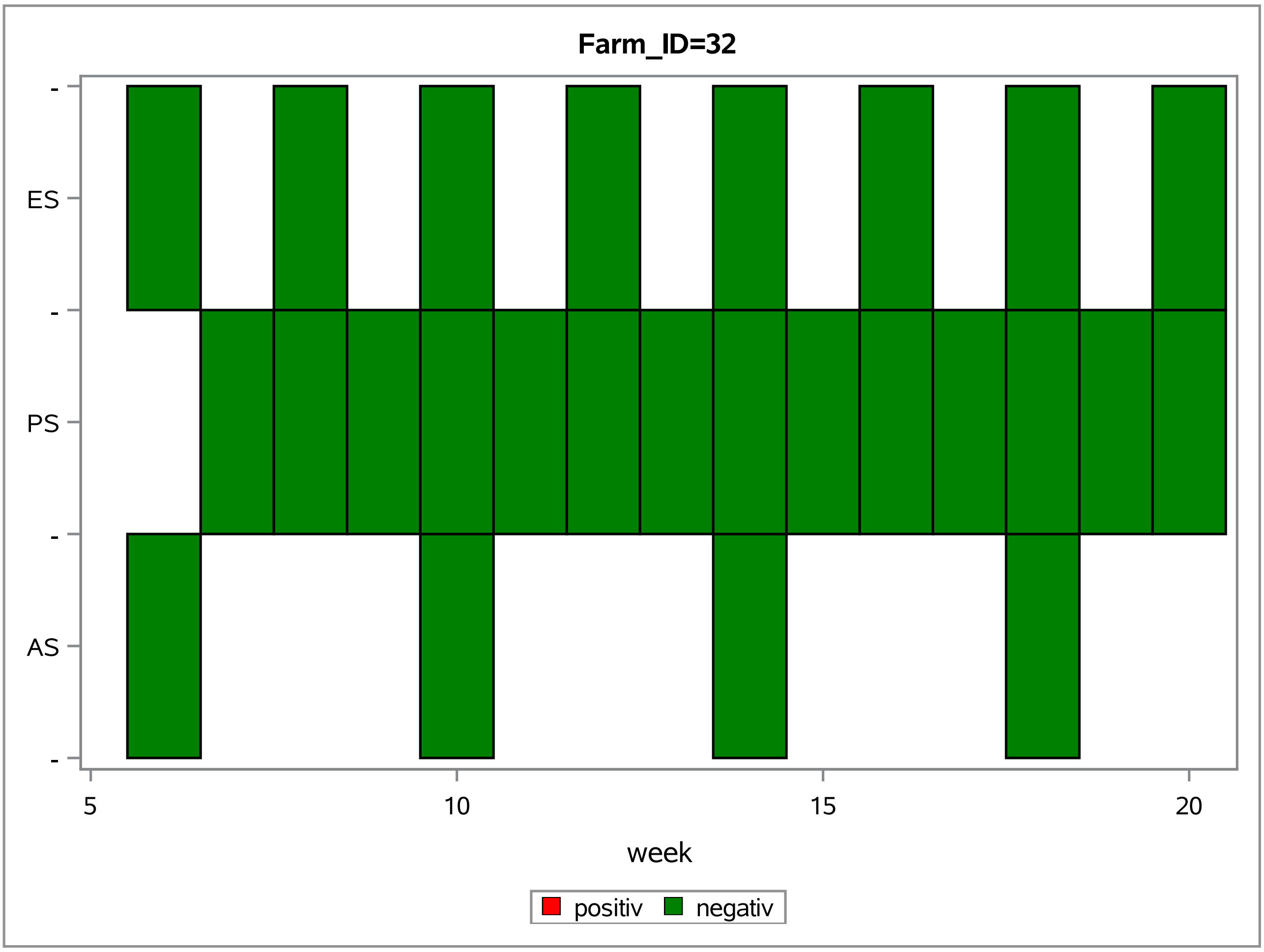
Farm_ID=32
ES
PS
AS
5
10
15
20
week
positiv
negativ

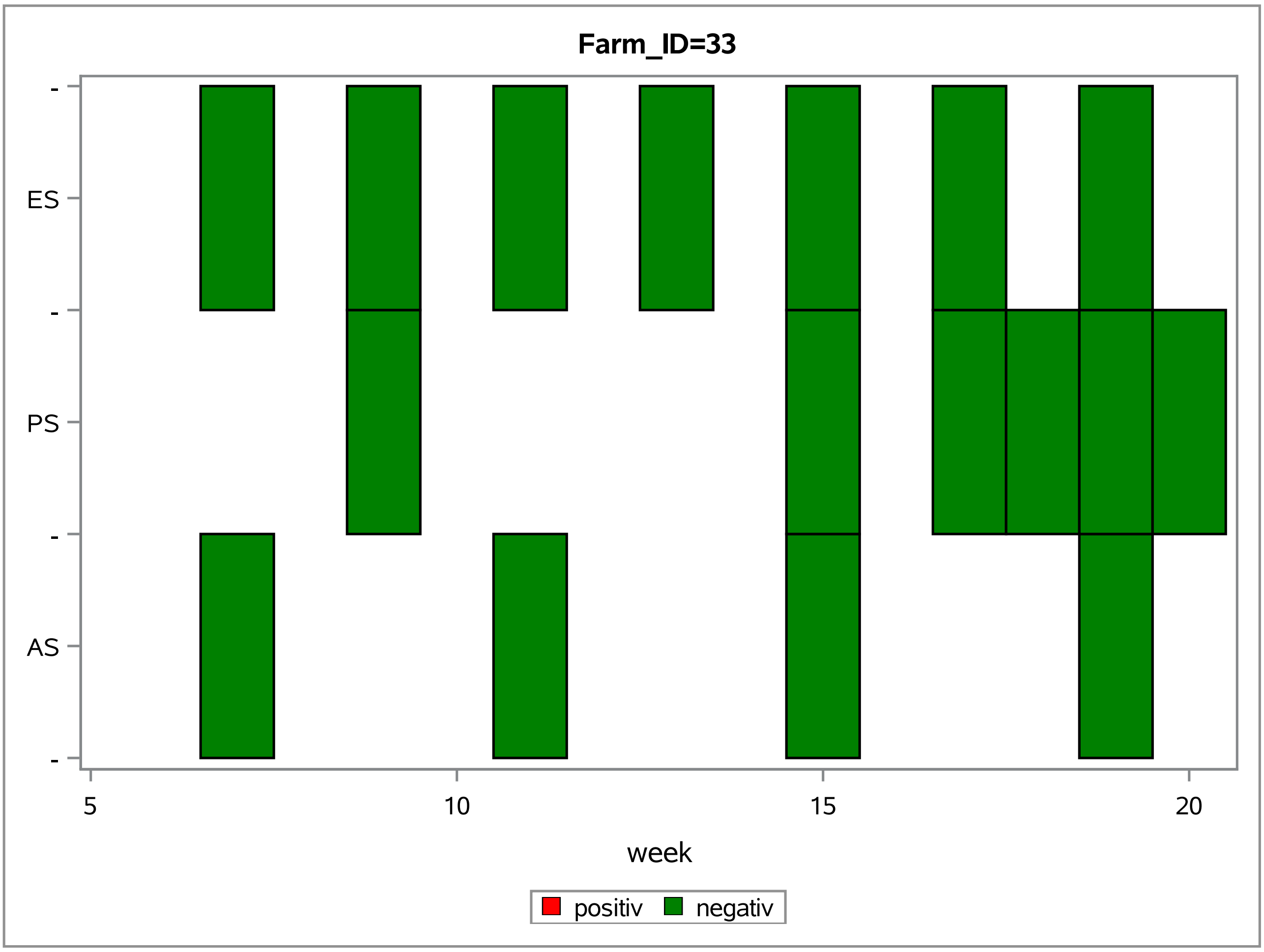
Farm_ID=33
ES
PS
AS
5
10
15
20
week
positiv
negativ

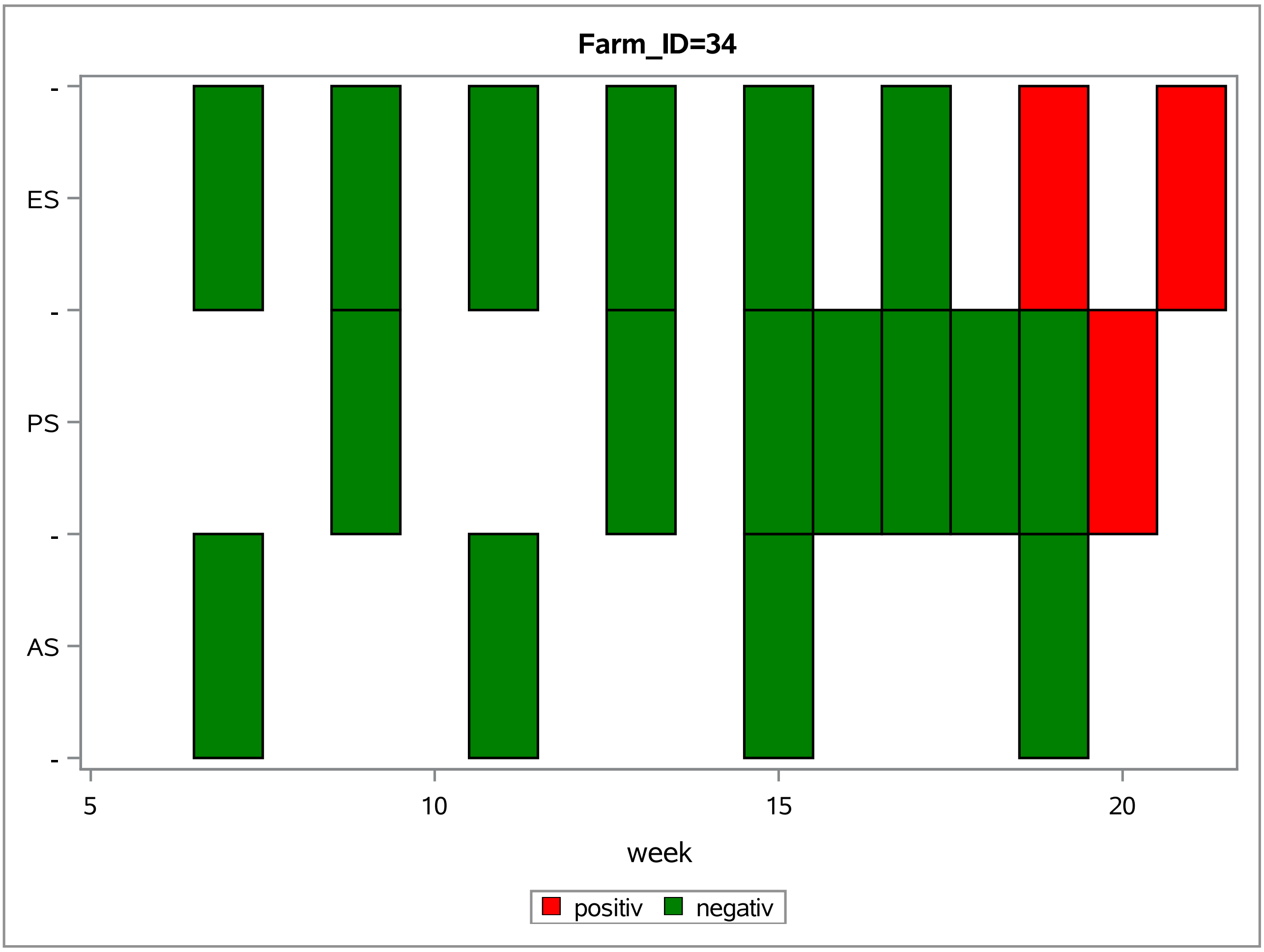
Farm_ID=34
ES
PS
AS
-
-
-
-
5
10
15
20
week
positiv
negativ

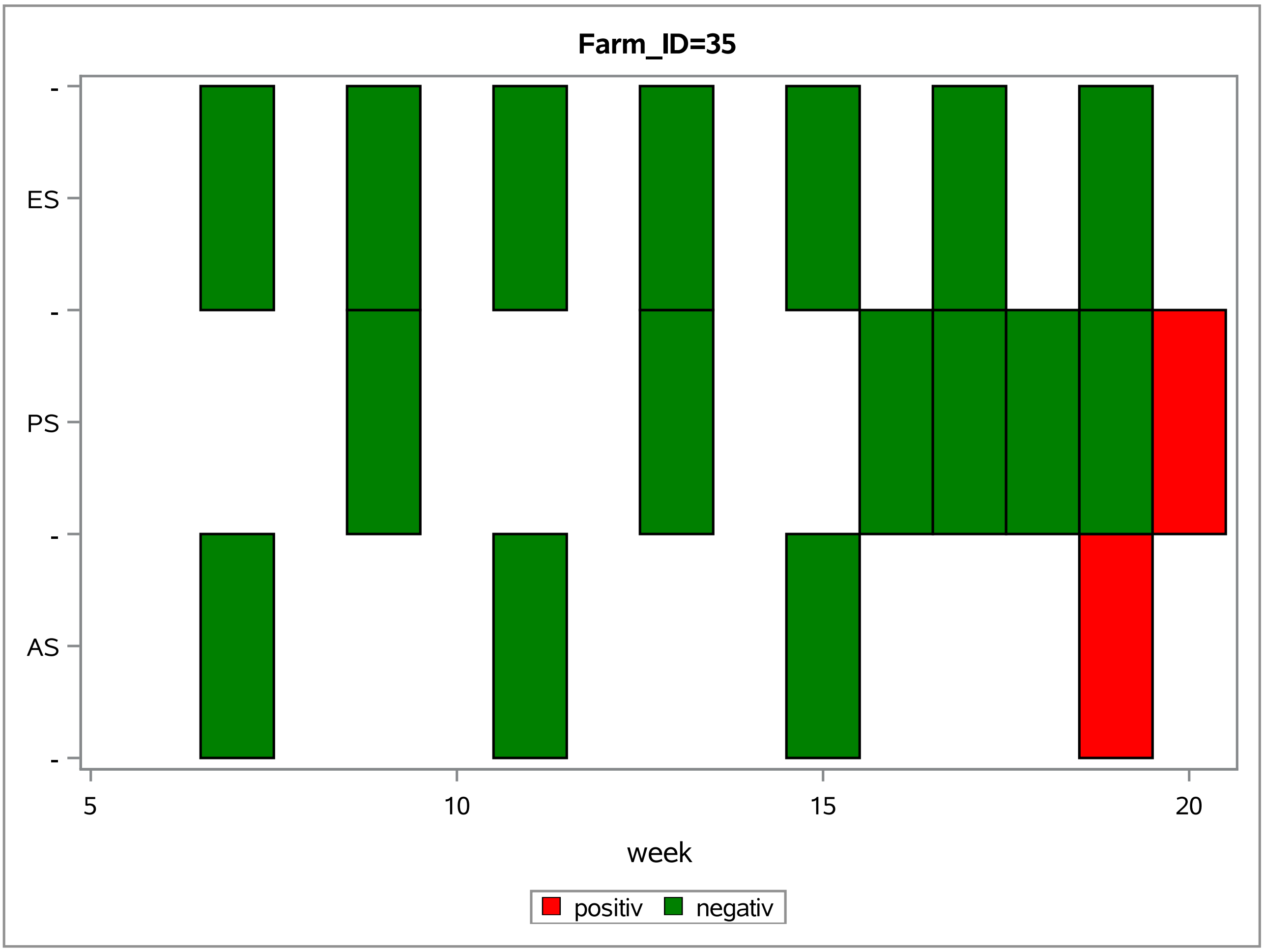
Farm_ID=35
ES
PS
AS
5
10
15
20
week
positiv
negativ

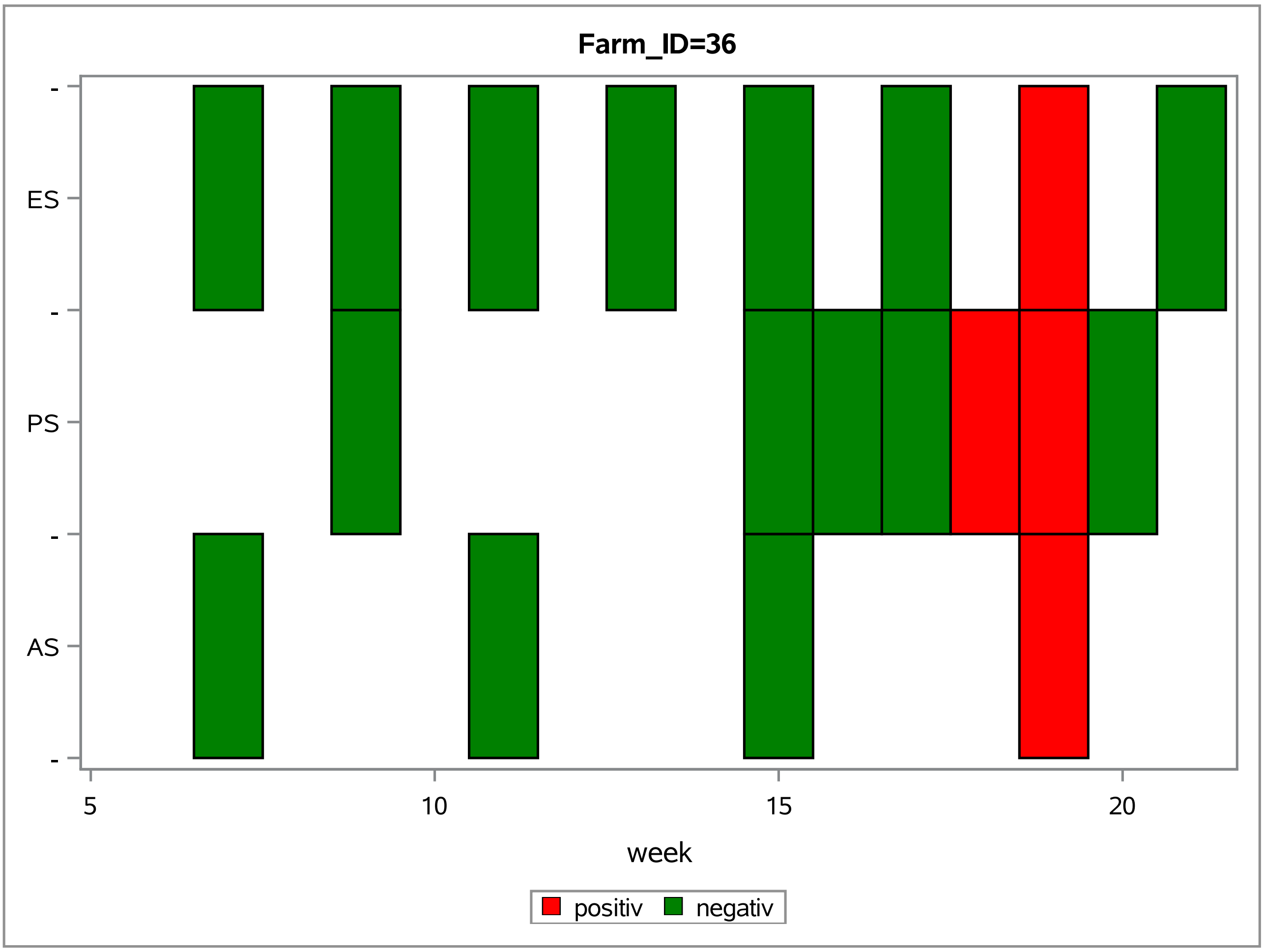
Farm_ID=36
ES
PS
AS
5
10
15
20
week
positiv
negativ

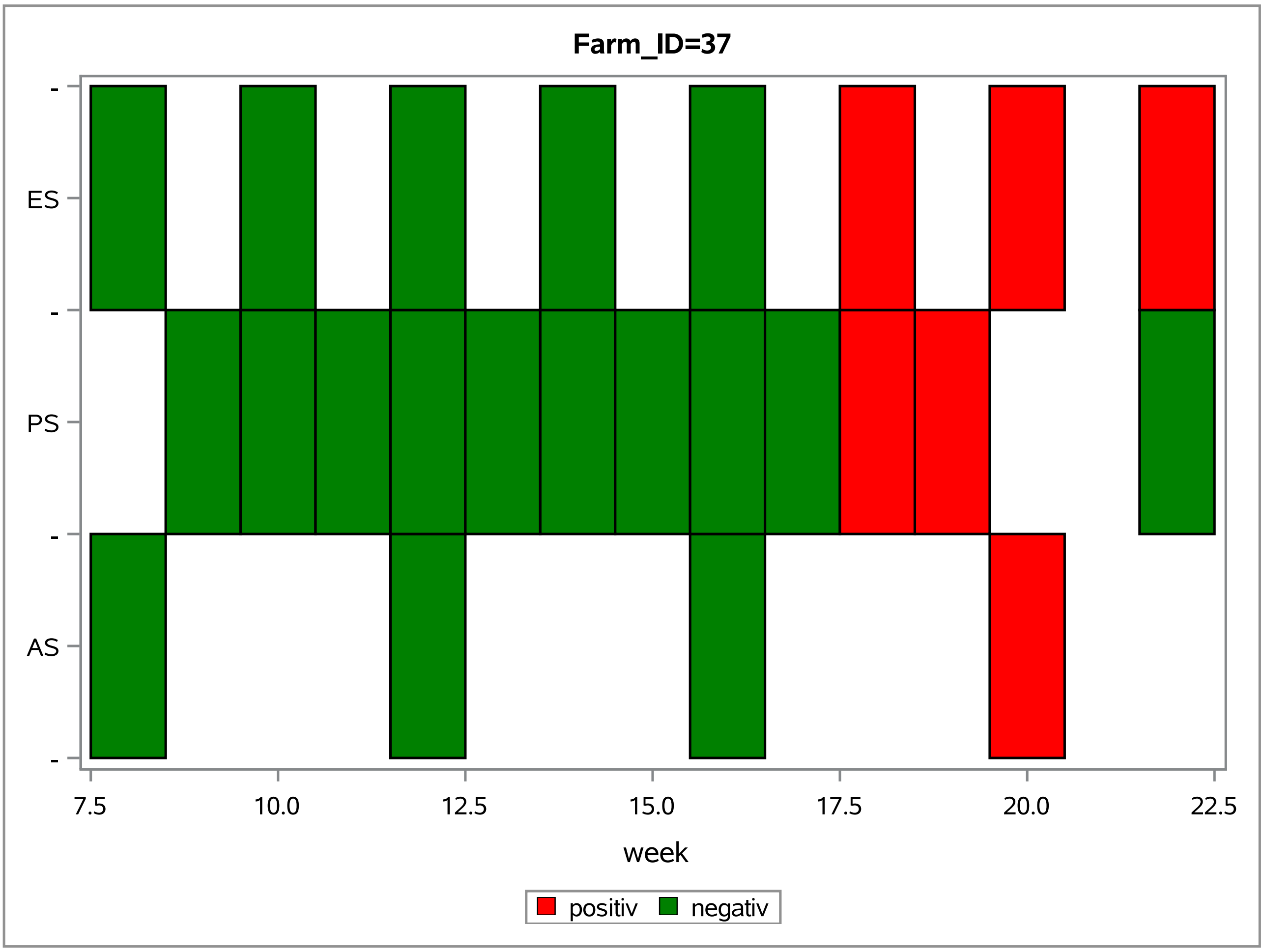
Farm_ID=37
-
ES
-
PS
-
AS
-
7.5
10.0
12.5
15.0
17.5
20.0
22.5
week
positiv
negativ

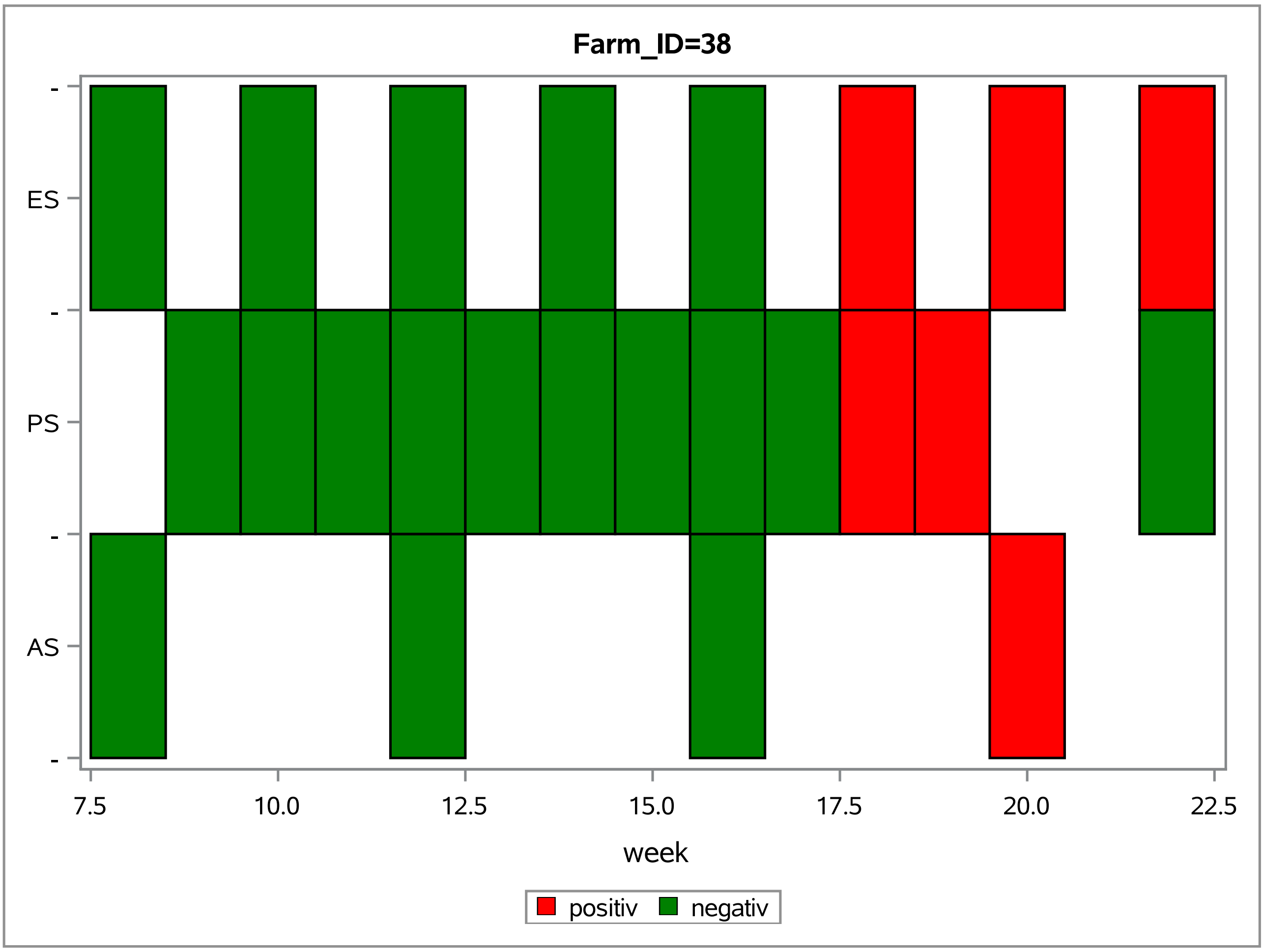
Farm_ID=38
ES
PS
AS
7.5
10.0
12.5
15.0
17.5
20.0
22.5
week
positiv
negativ

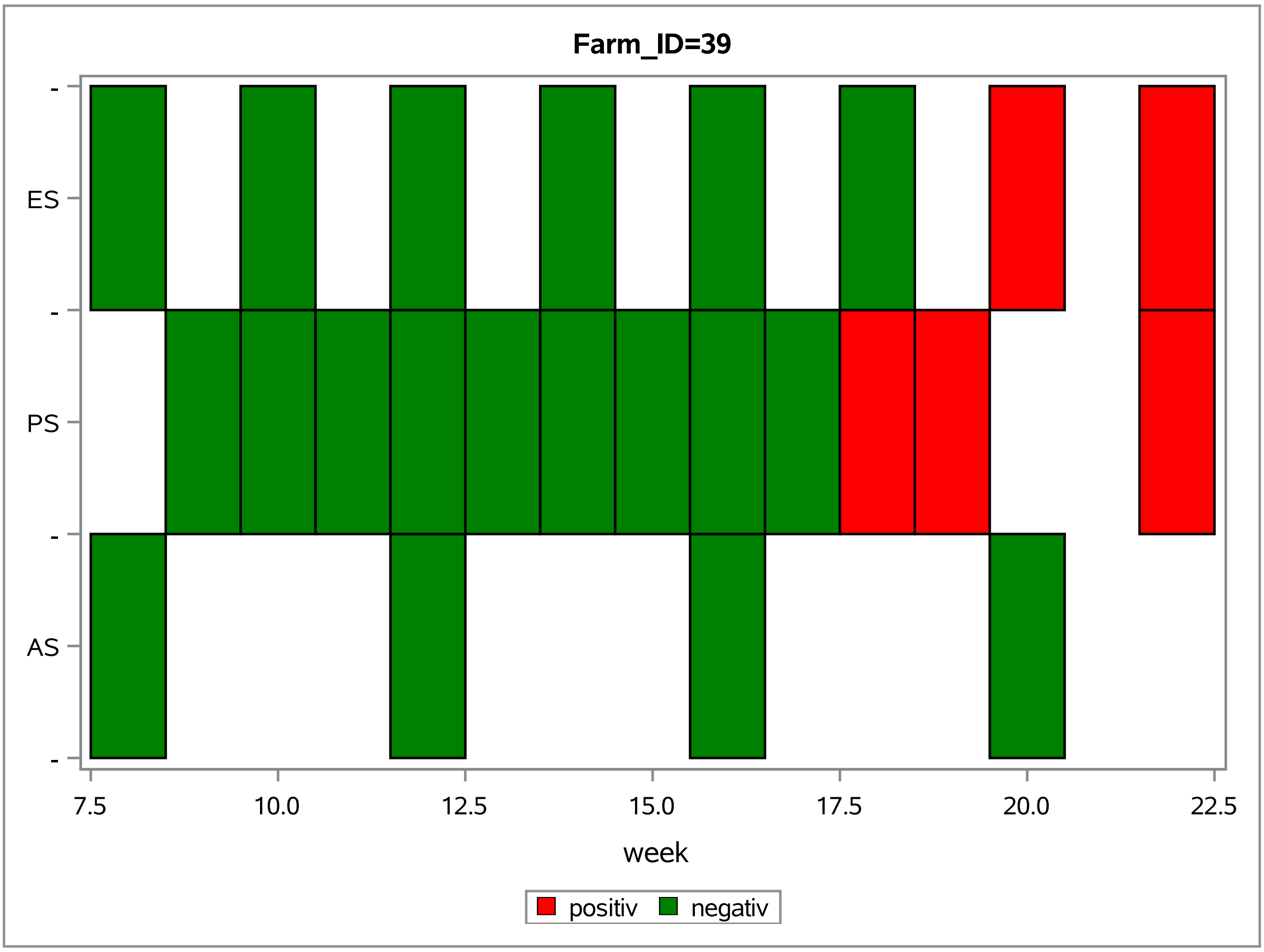
Farm_ID=39
ES
PS
AS
7.5
10.0
12.5
15.0
17.5
20.0
22.5
week
positiv
negativ

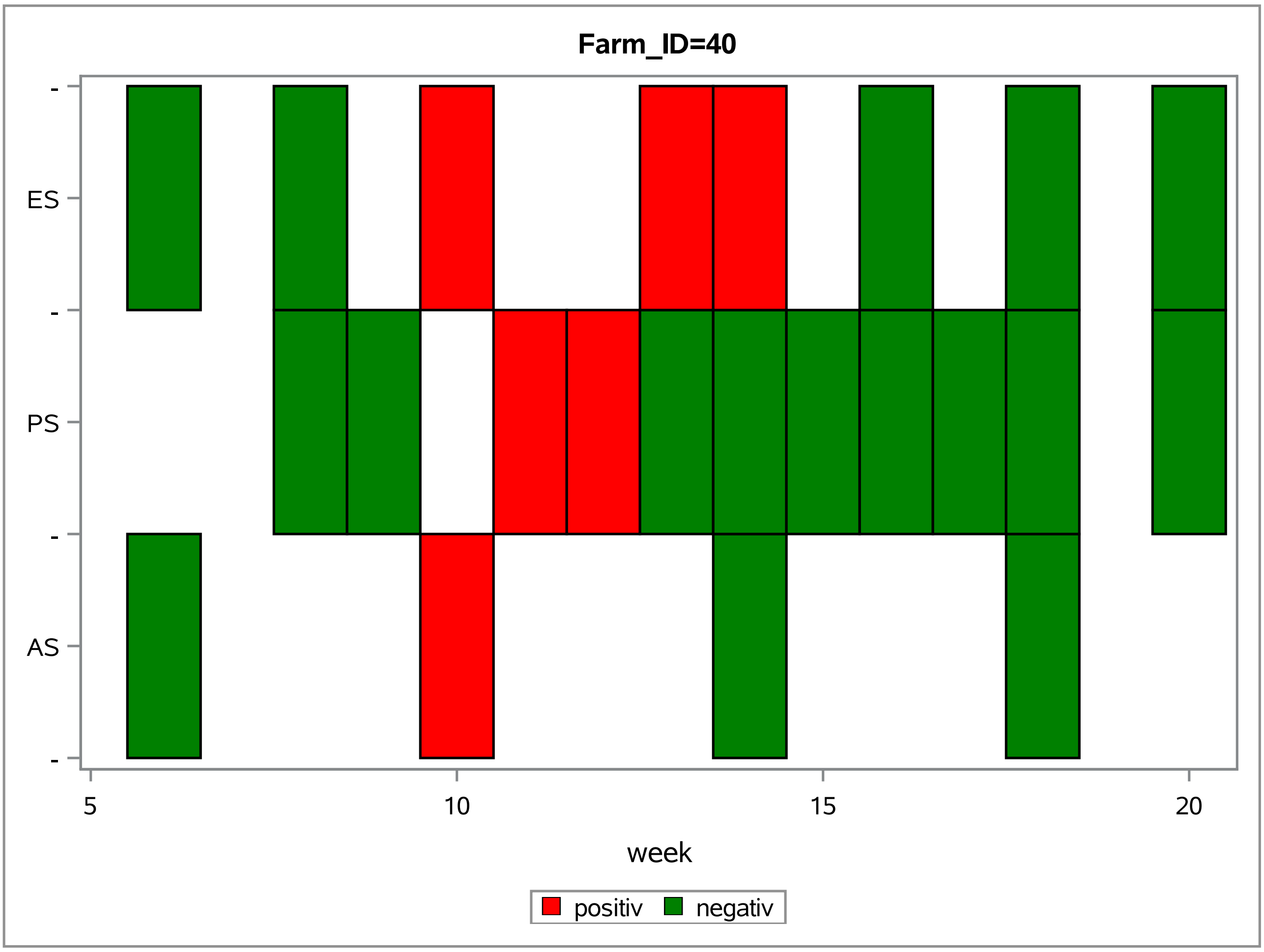
Farm_ID=40
ES
PS
AS
5
10
15
20
week
positiv
negativ

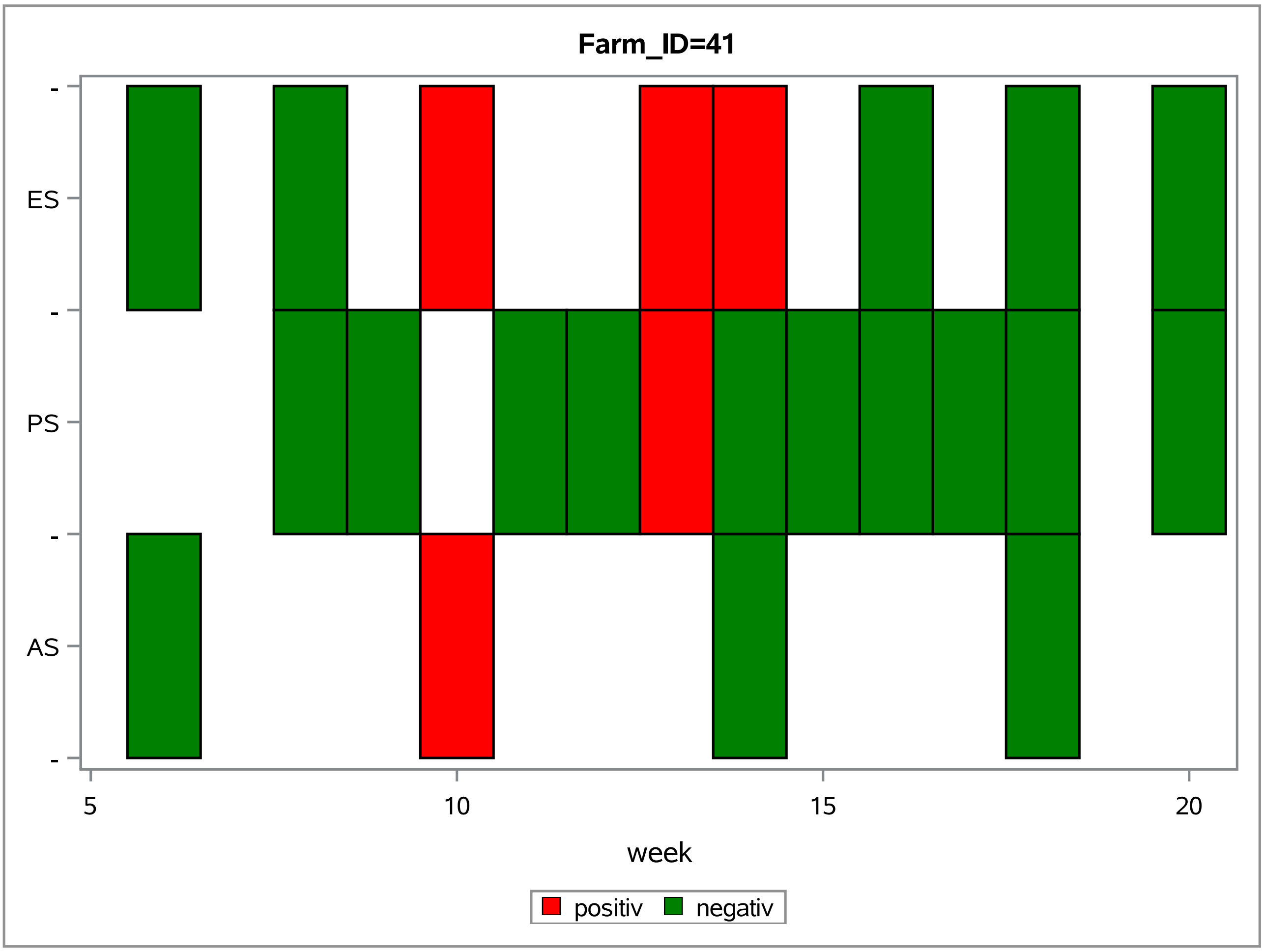
Farm_ID=41
ES
PS
AS
5
10
15
20
week
positiv
negativ

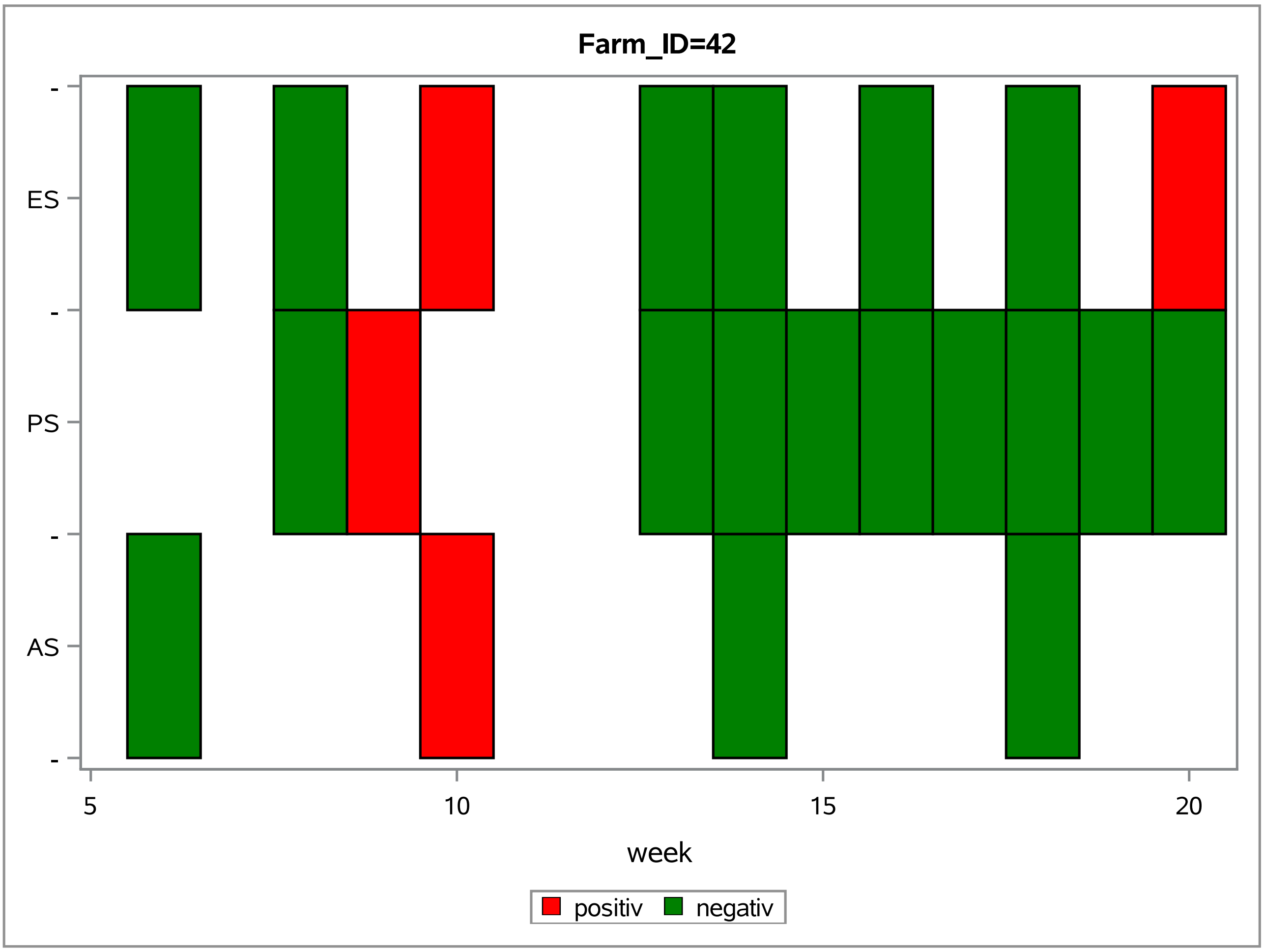
Farm_ID=42
ES
PS
AS
5
10
15
20
week
positiv
negativ

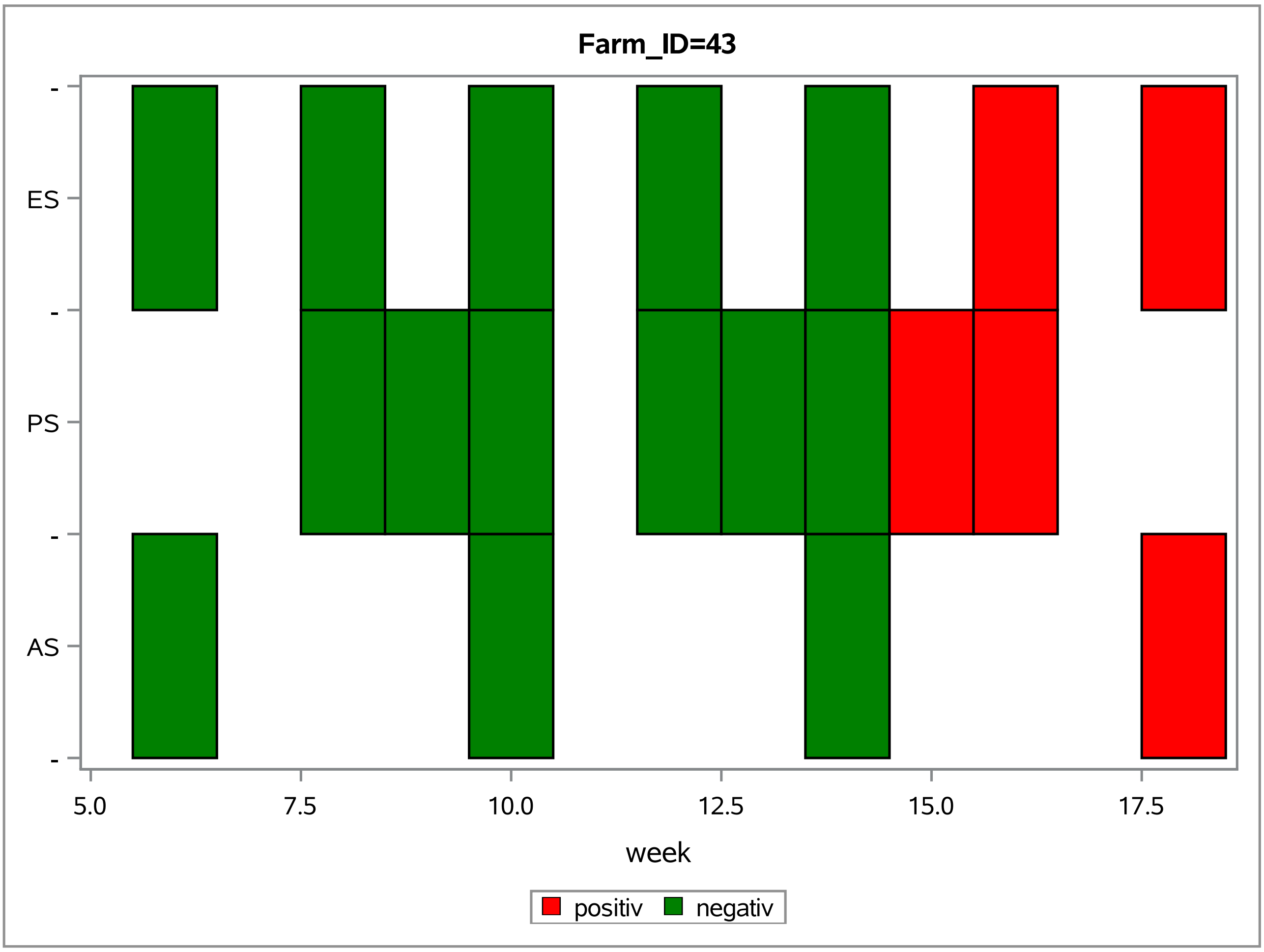
Farm_ID=43
ES
PS
AS
5.0
7.5
10.0
12.5
15.0
17.5
week
positiv
negativ

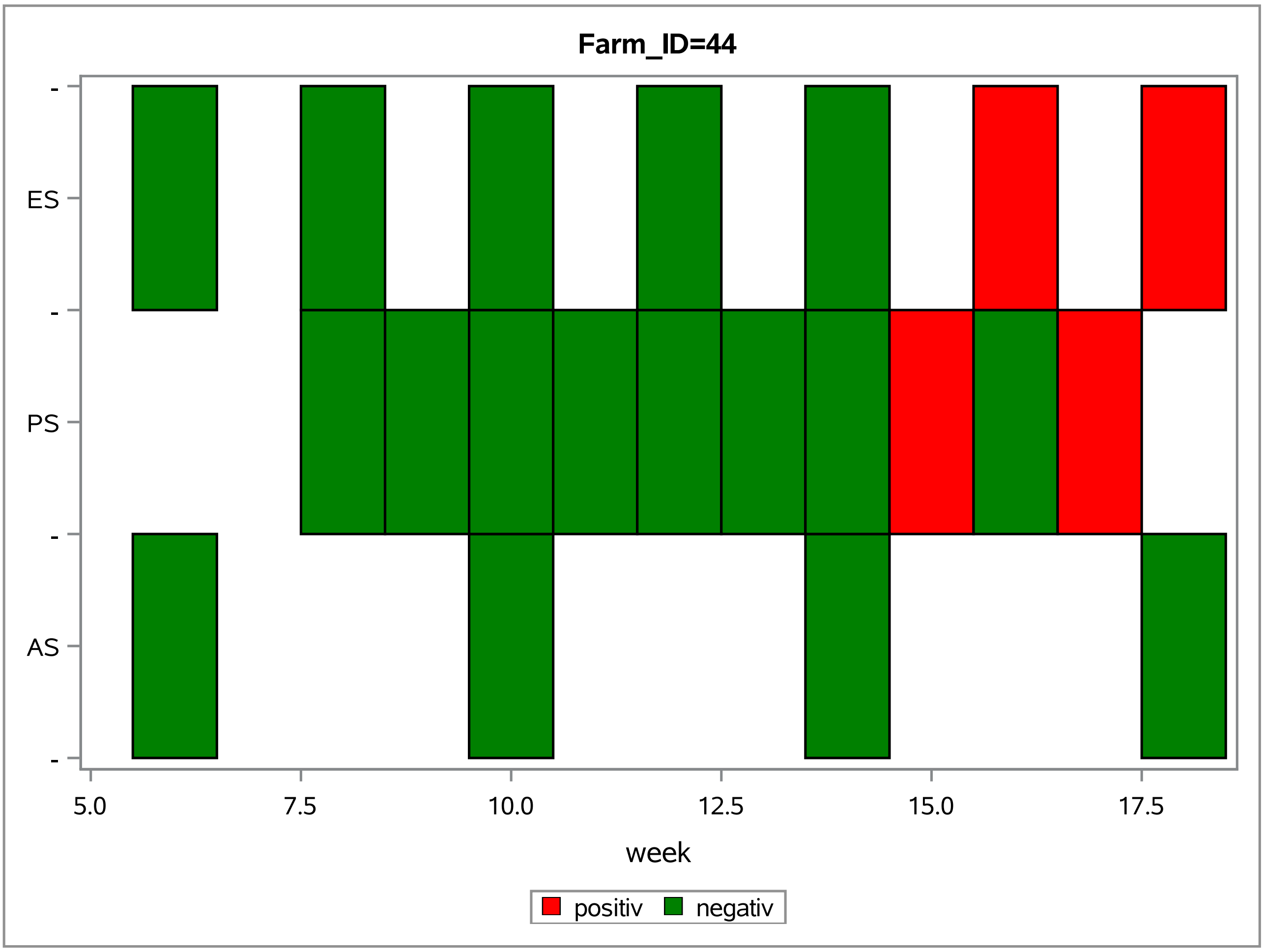
Farm_ID=44
ES
PS
AS
5.0
7.5
10.0
12.5
15.0
17.5
week
positiv
negativ

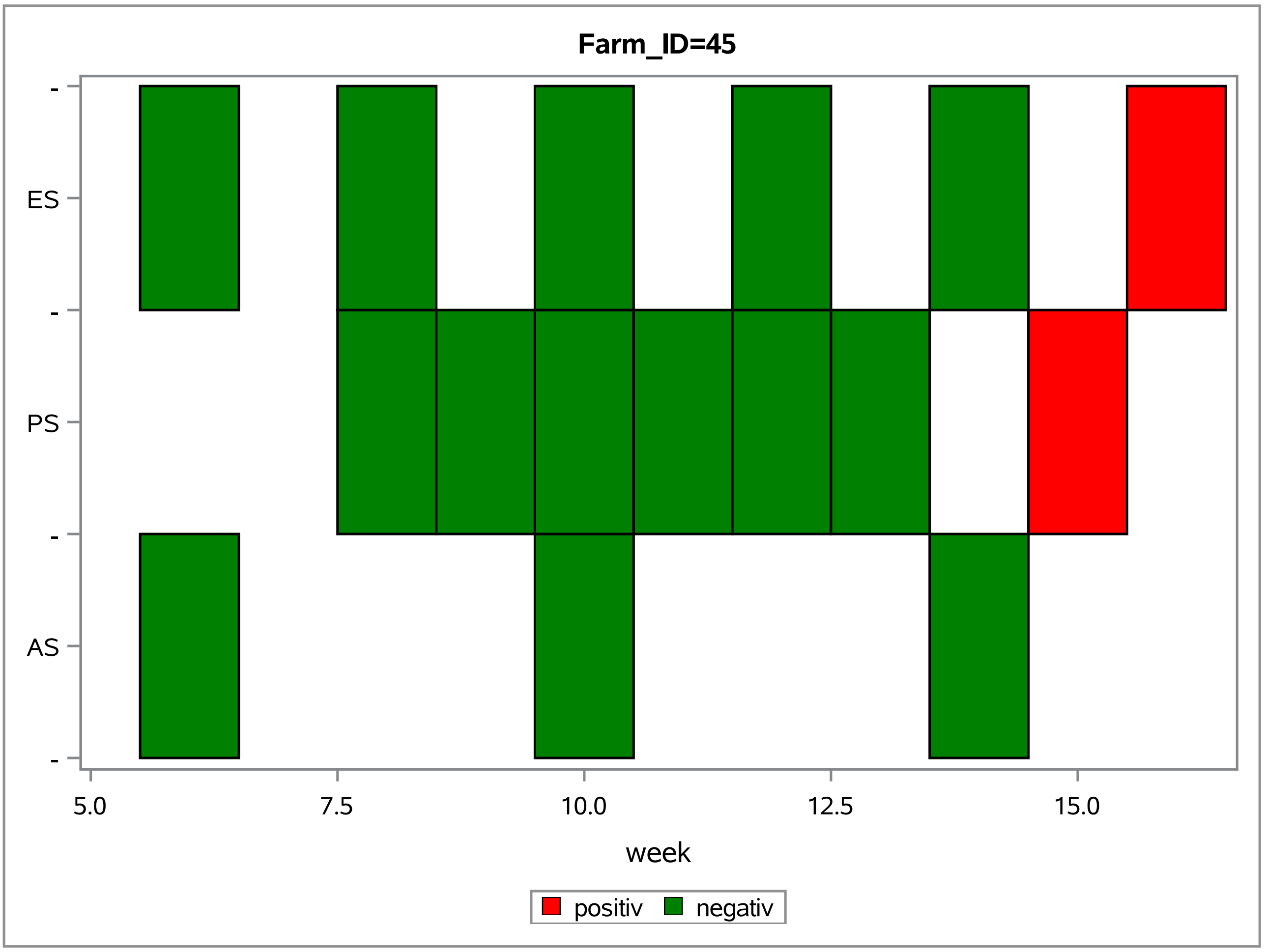
Farm_ID=45
ES
PS
AS
5.0
7.5
10.0
12.5
15.0
week
positiv
negativ

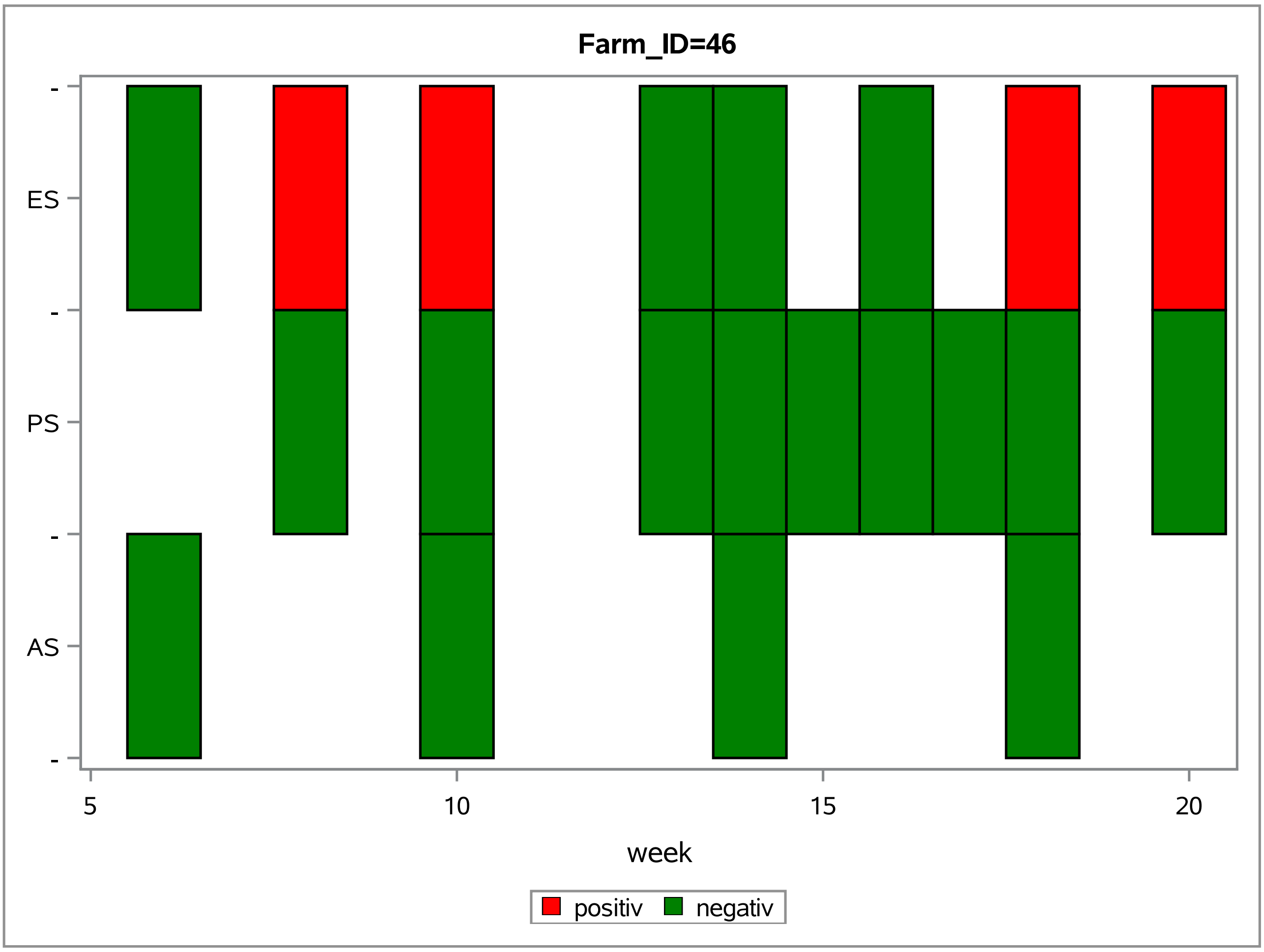
Farm_ID=46
ES
PS
AS
-
5
10
15
20
week
positiv
negativ

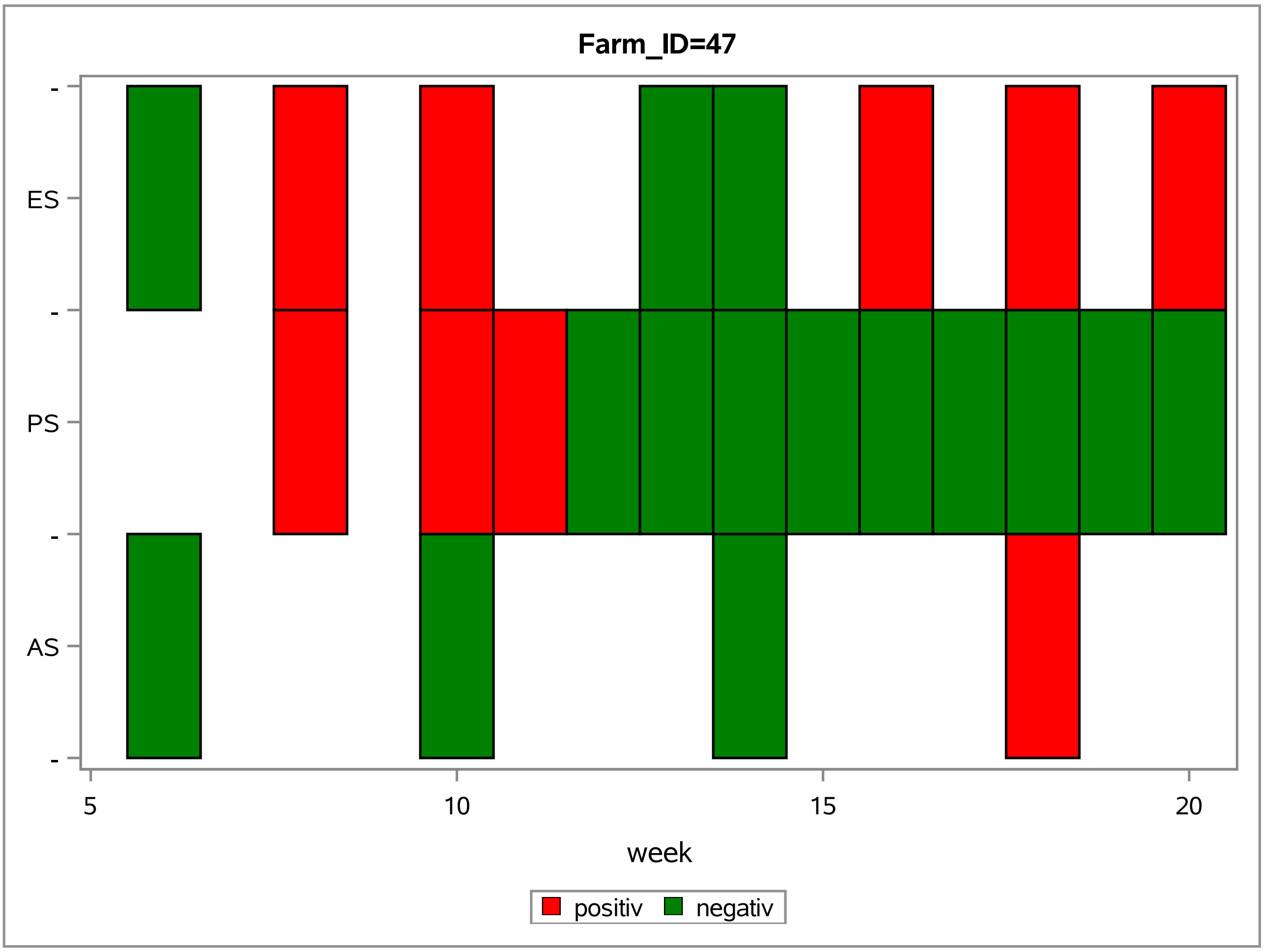

Farm_ID=47
-
ES
-
PS
-
AS
-
5
10
15
20
week
positiv
negativ

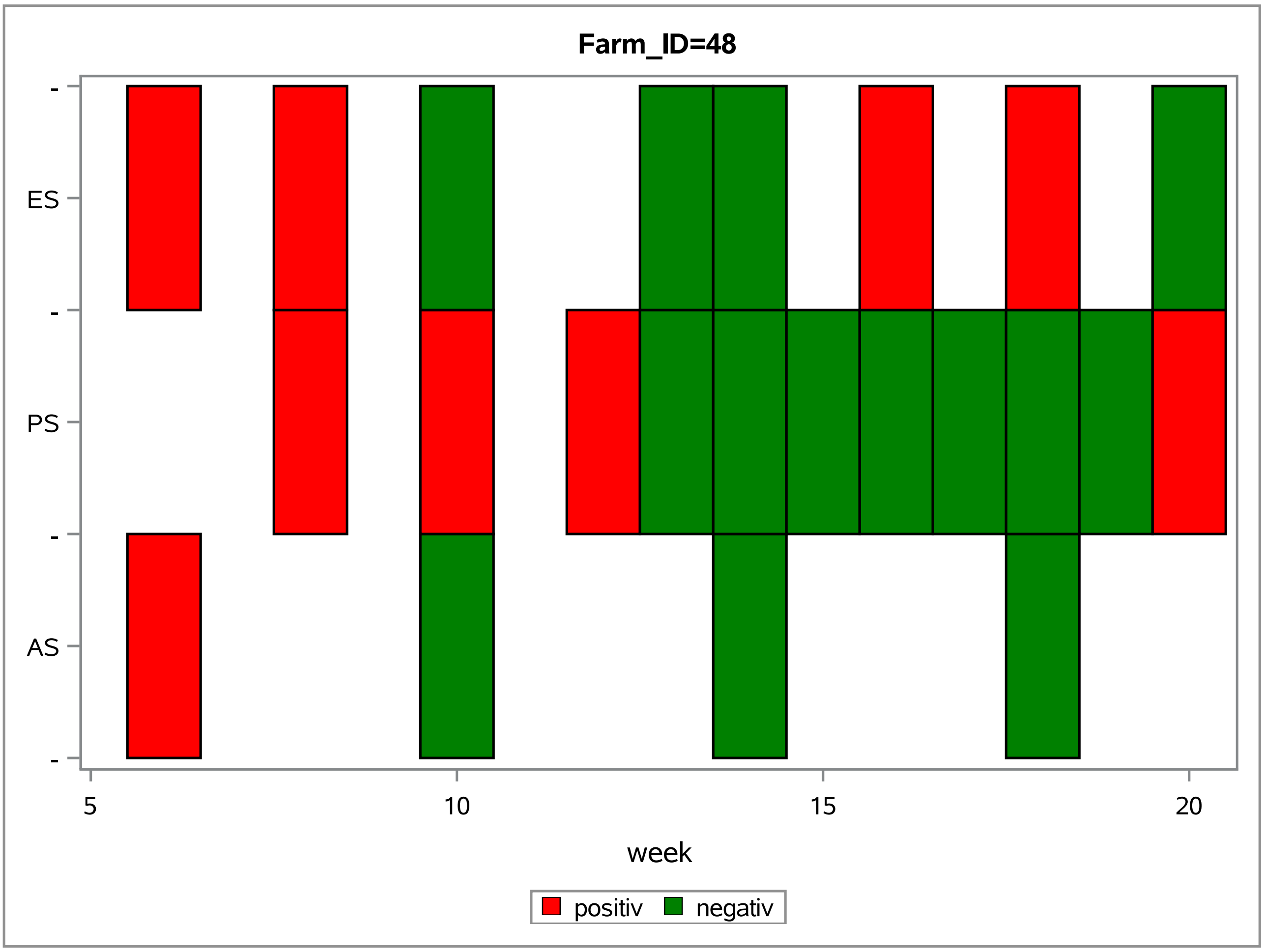
Farm_ID=48
ES
PS
AS
5
10
15
20
week
positiv
negativ

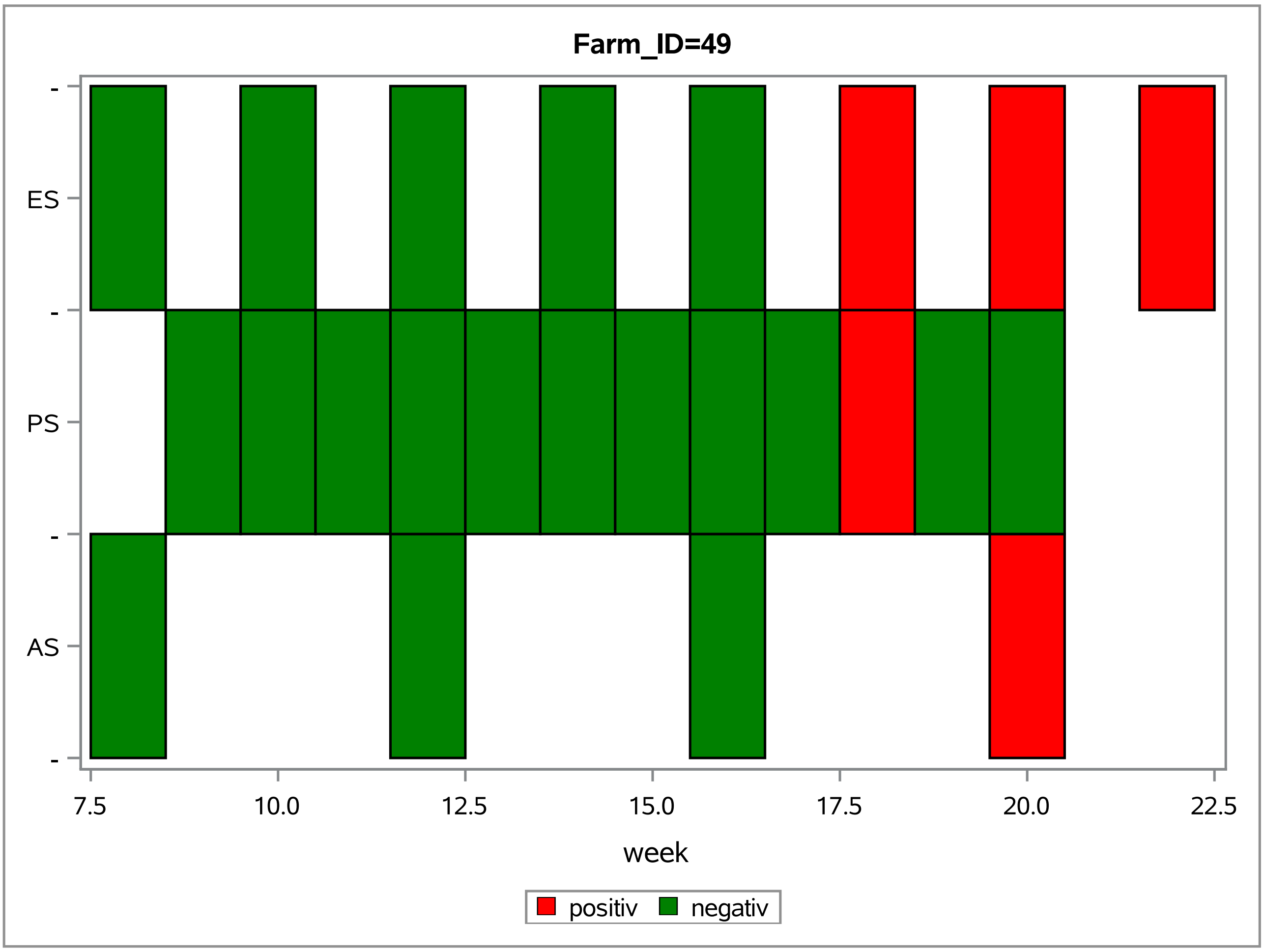
Farm_ID=49
ES
PS
AS
7.5
10.0
12.5
15.0
17.5
20.0
22.5
week
positiv
negativ

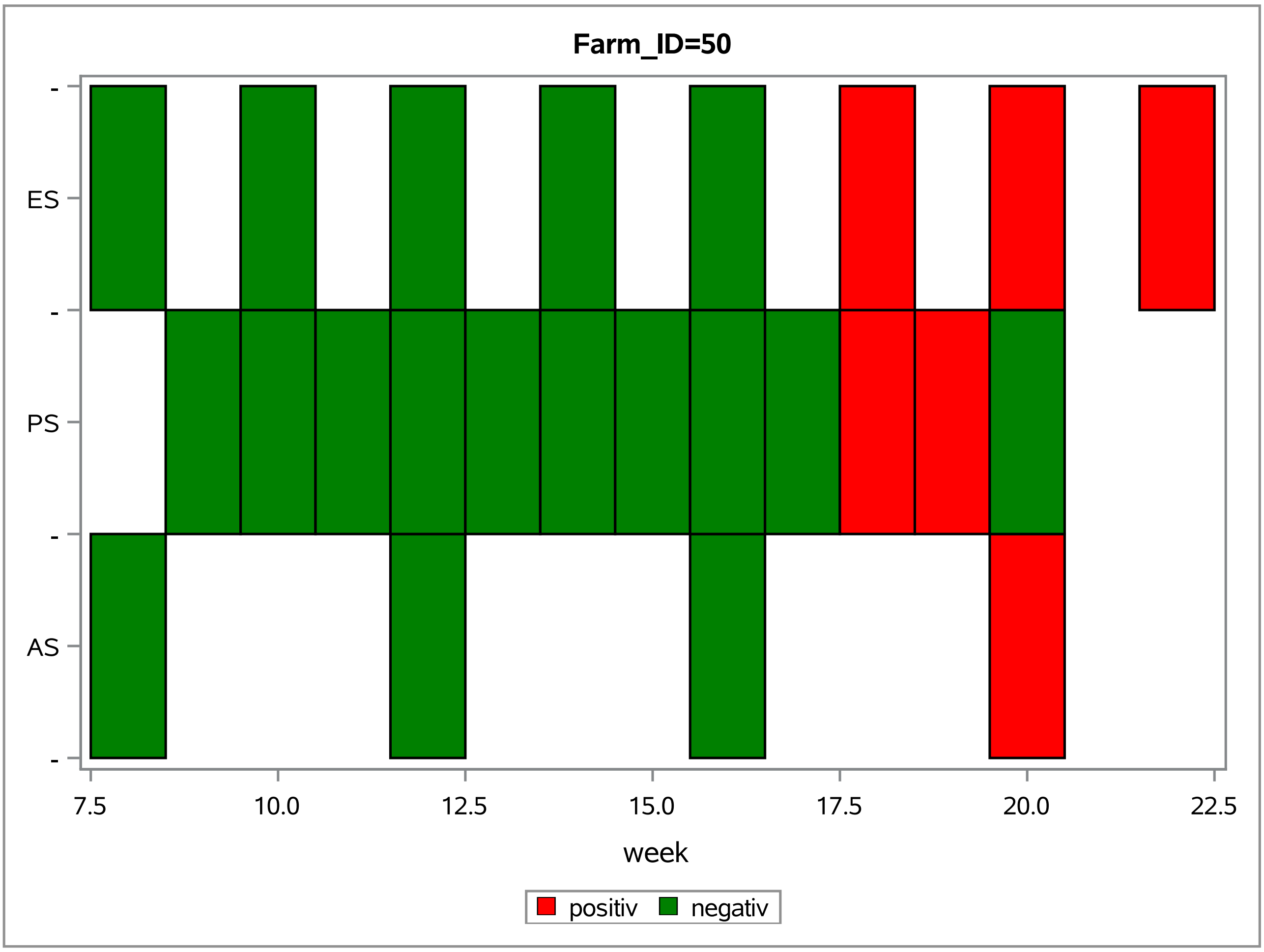

Farm_ID=50
ES
PS
AS
7.5
10.0
12.5
15.0
17.5
20.0
22.5
week
positiv
negativ

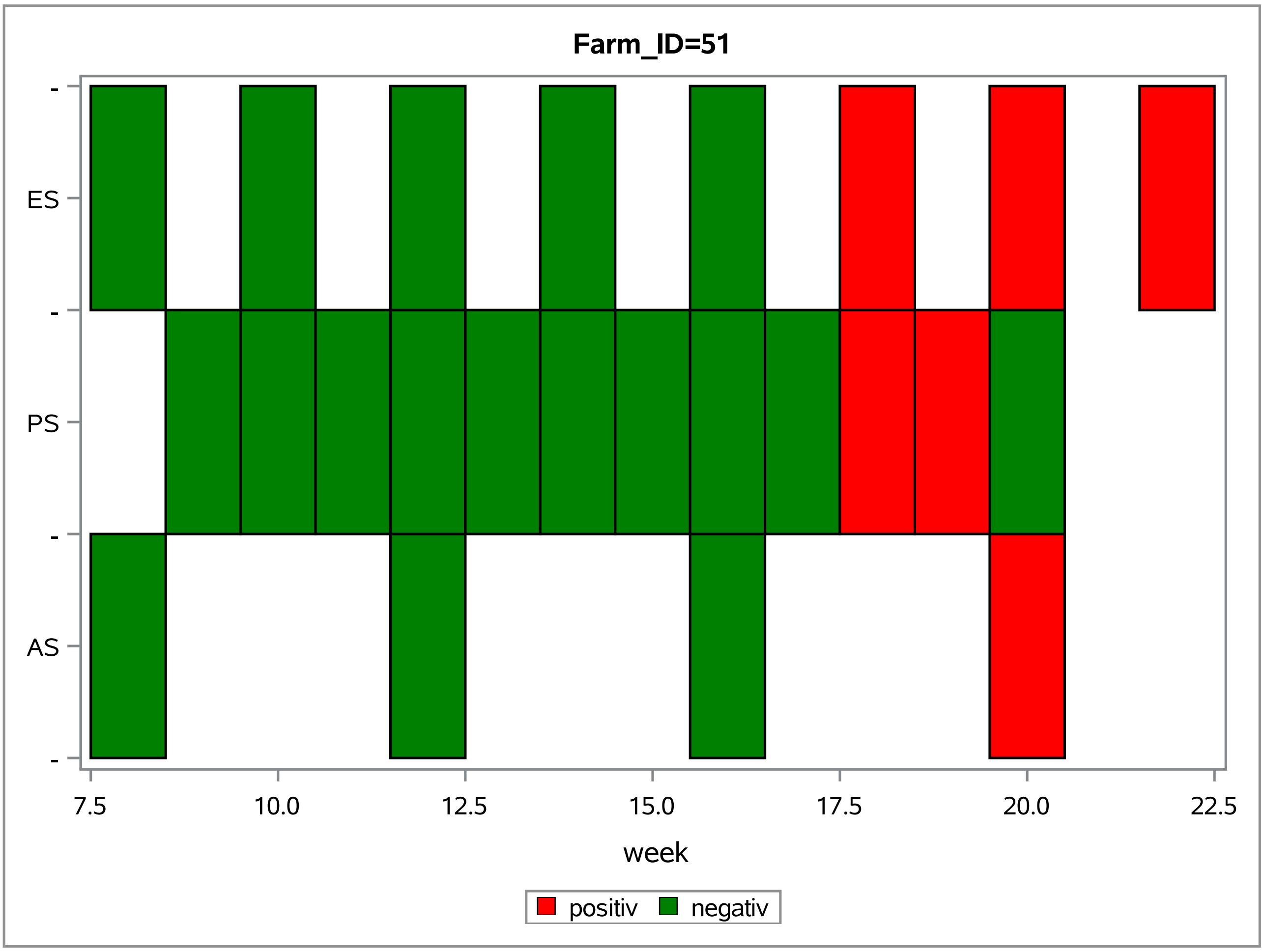
Farm_ID=51
ES
PS
AS
7.5
10.0
12.5
15.0
17.5
20.0
22.5
week
positiv
negativ

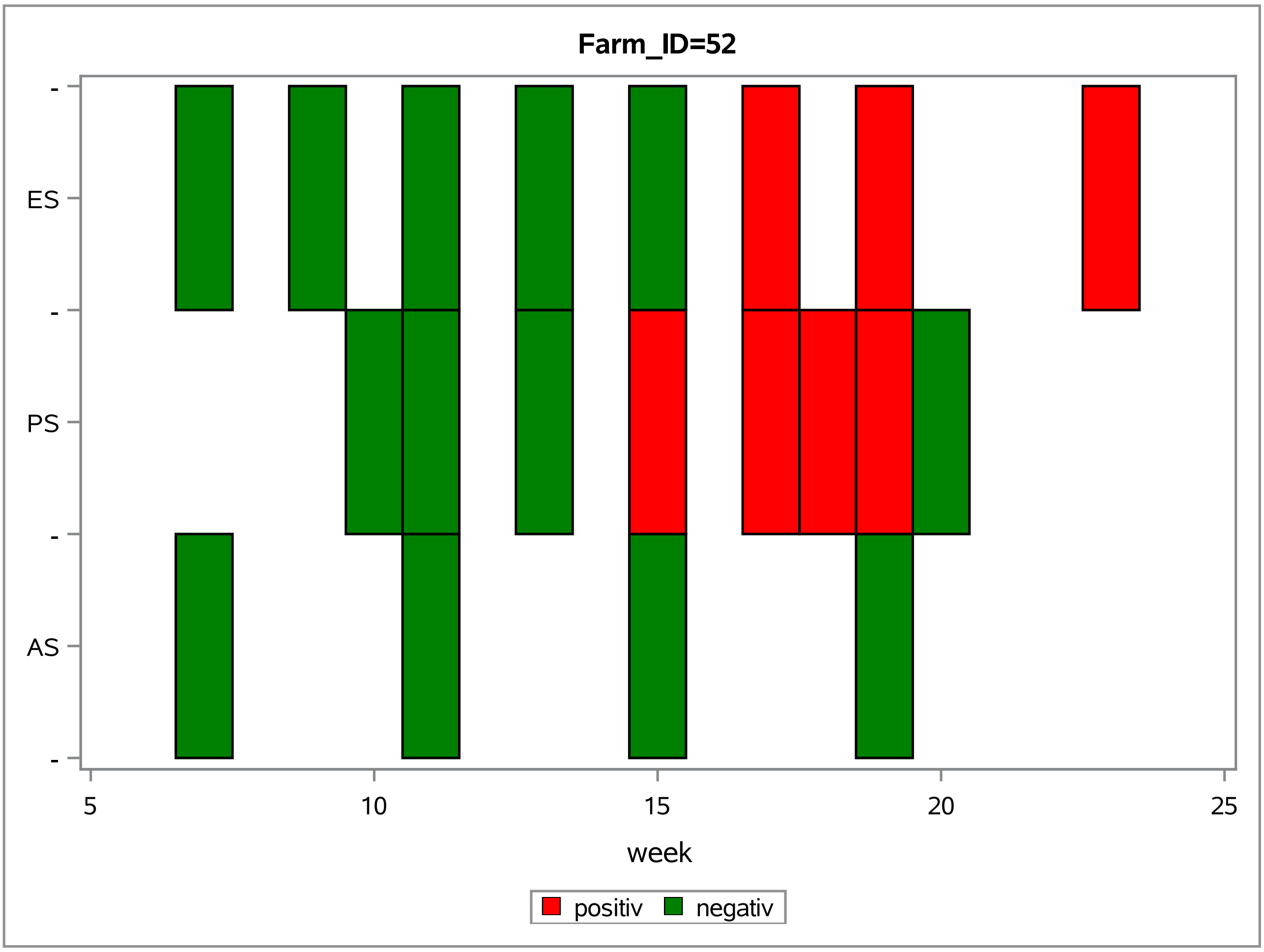
Farm_ID=52
ES
PS
AS
-
5
10
15
20
25
week
positiv
negativ

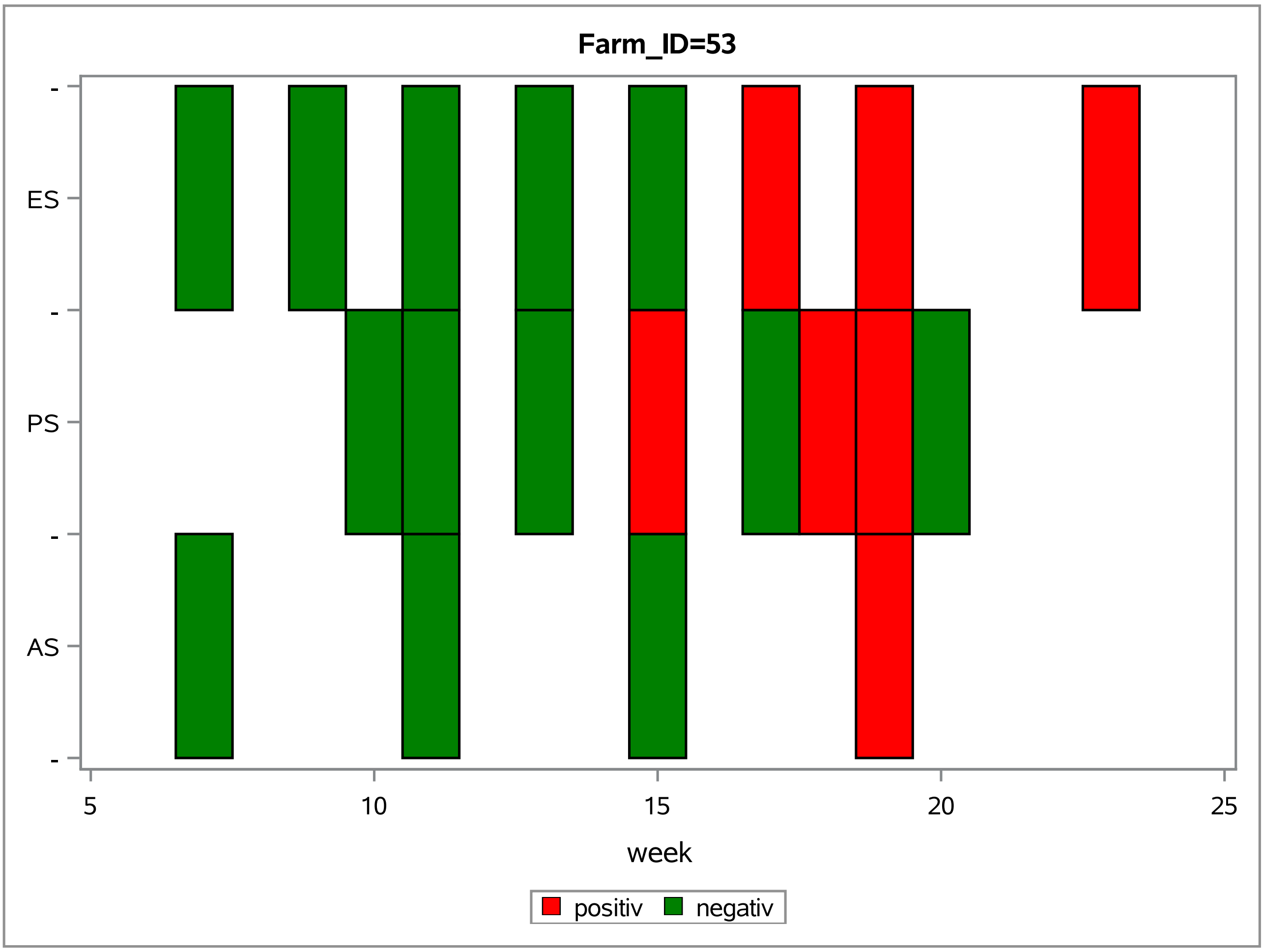
Farm_ID=53
ES
PS
AS
5
10
15
20
25
week
positiv
negativ

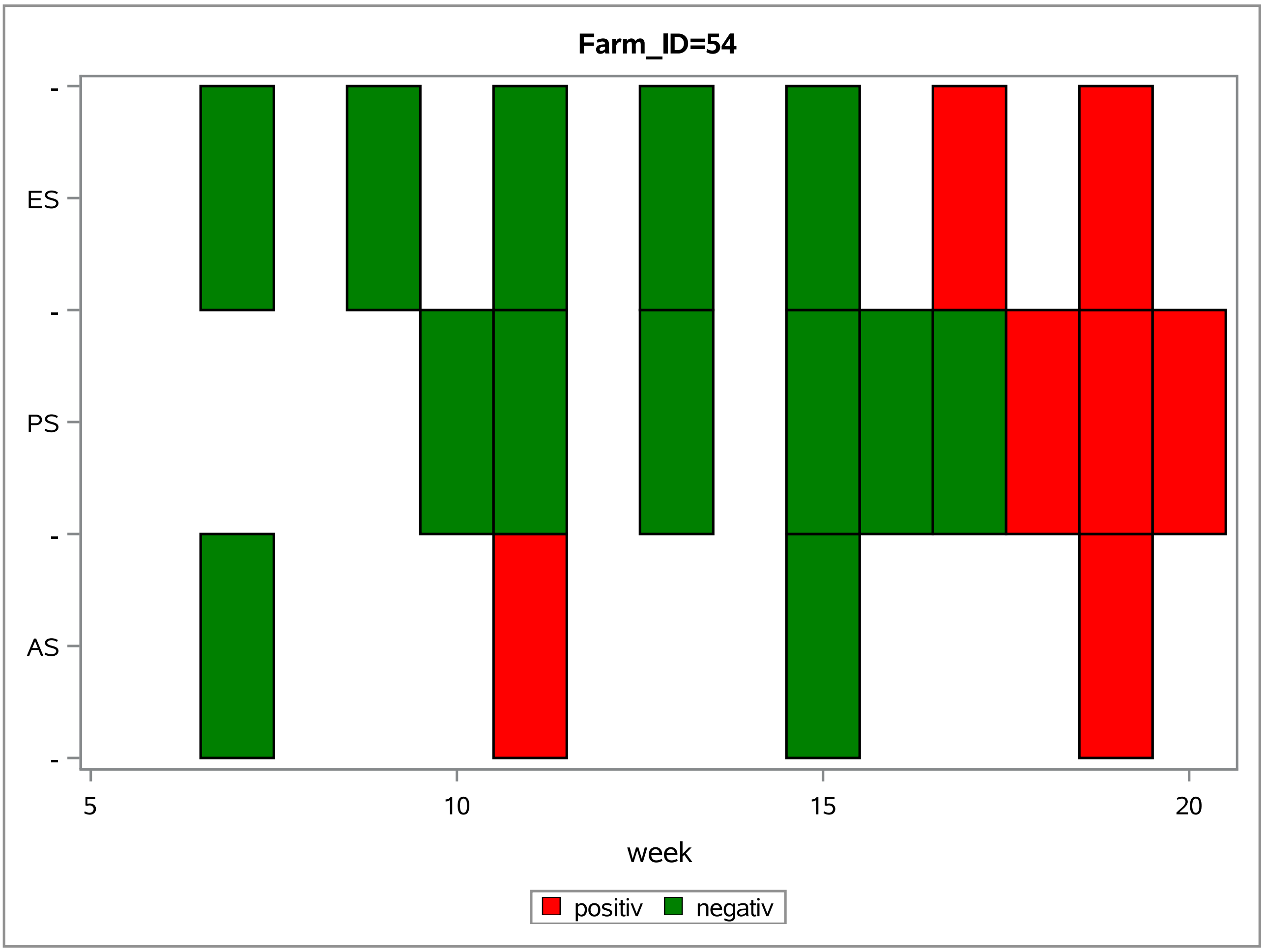
Farm_ID=54
ES
PS
AS
5
10
15
20
week
positiv
negativ

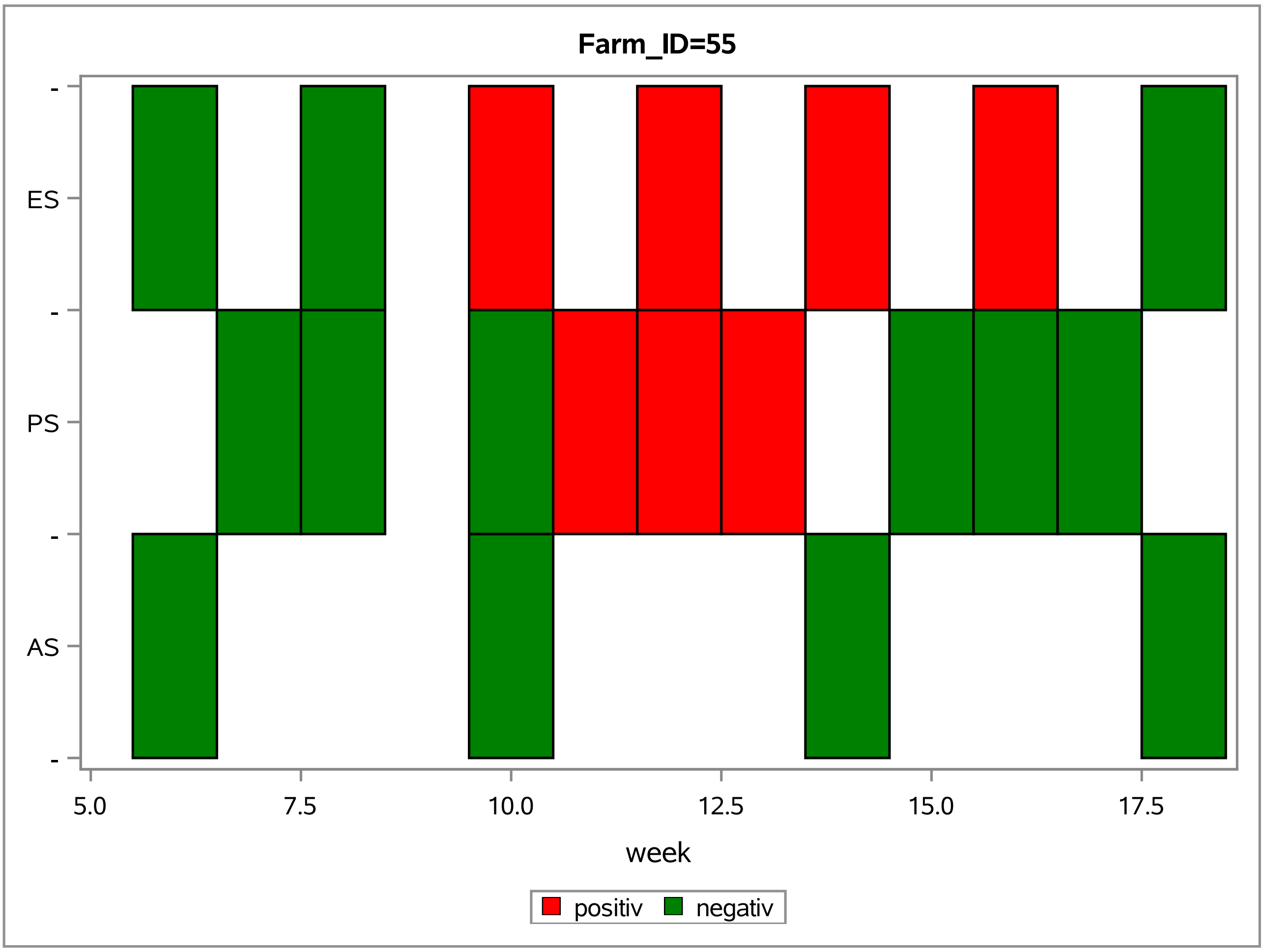
Farm_ID=55
ES
PS
AS
5.0
7.5
10.0
12.5
15.0
17.5
week
positiv
negativ

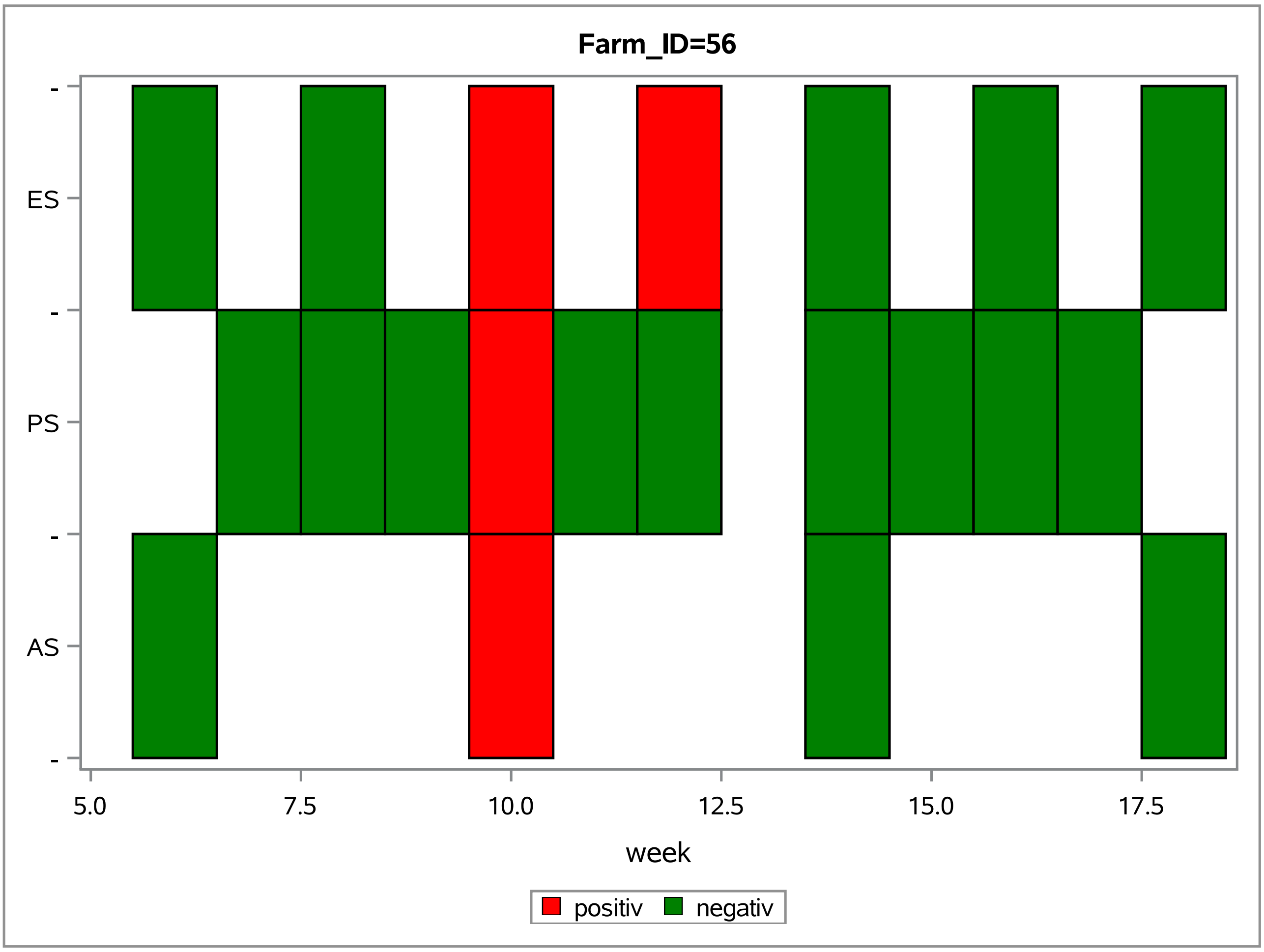
Farm_ID=56
ES
PS
AS
5.0
7.5
10.0
12.5
15.0
17.5
week
positiv
negativ
